# Minimax Estimation for Personalized Federated Learning: An Alternative between FedAvg and Local Training?

**Published:** 2023

**Authors:** Shuxiao Chen, Qinqing Zheng, Qi Long, Weijie J. Su

**Affiliations:** ⋆ University of Pennsylvania; † Meta AI Research

**Keywords:** Empirical Risk Minimization, Federated Learning, Personalization, Data Heterogeneity, Minimax Rates, Algorithmic Stability

## Abstract

A widely recognized difficulty in federated learning arises from the statistical heterogeneity among clients: local datasets often originate from distinct yet not entirely unrelated probability distributions, and personalization is, therefore, necessary to achieve optimal results from each individual’s perspective. In this paper, we show how the excess risks of personalized federated learning using a smooth, strongly convex loss depend on data heterogeneity from a minimax point of view, with a focus on the FedAvg algorithm (McMahan et al., 2017) and pure local training (i.e., clients solve empirical risk minimization problems on their local datasets without any communication). Our main result reveals an *approximate* alternative between these two baseline algorithms for federated learning: the former algorithm is minimax rate optimal over a collection of instances when data heterogeneity is small, whereas the latter is minimax rate optimal when data heterogeneity is large, and the threshold is sharp up to a constant.

As an implication, our results show that from a worst-case point of view, a dichotomous strategy that makes a choice between the two baseline algorithms is rate-optimal. Another implication is that the popular FedAvg following by local fine tuning strategy is also minimax optimal under additional regularity conditions. Our analysis relies on a new notion of algorithmic stability that takes into account the nature of federated learning.

## 1. Introduction

As one of the most important ingredients driving the success of machine learning, data are being generated and subsequently stored in an increasingly decentralized fashion in many real-world applications. For example, mobile devices will in a single day collect an unprecedented amount of data from users. These data commonly contain sensitive information such as web search histories, online shopping records, and health information, and thus are often not available to service providers (Poushter, 2016). This decentralized nature of (sensitive) data poses substantial challenges to many machine learning tasks.

To address this issue, McMahan et al. (2017) proposed a new learning paradigm, which they termed *federated learning*, for collaboratively training machine learning models on data that are locally possessed by multiple clients with the coordination of the central server (e.g., service provider), without having direct access to the local datasets. In its simplest form, federated learning considers a pool of m clients, where the i-th client has a local dataset $S_{i}$ of size $n_{i}$, consisting of i.i.d. samples $\left\{z_{j}^{\left(i\right)}:j\in \left[n_{i}\right]\right\}$ (denote [n]≔{1,2,…,n}) from some unknown distribution ${𝒟}_{i}$. Letting ℓ(w,z) be a loss function, where w denotes the model parameter, the optimal local model for the i-th client is given by

(1) $$w_{⋆}^{\left(i\right)}\in \underset{w}{\text{argmin }}E_{Z_{i}\sim {𝒟}_{i}}\ell \left(w,Z_{i}\right).$$

From the *client-wise* perspective, any data-dependent estimator ${\hat{w}}^{\left(i\right)}(S)$, with $S={\left\{S_{i}\right\}}_{i=1}^{m}$ denoting the collection of all samples, can be evaluated based on its individualized excess risk:

$${\text{IER}}_{i}≔E_{Z_{i}\sim {𝒟}_{i}}\left[\ell \left({\hat{w}}^{\left(i\right)},Z_{i}\right)-\ell \left(w_{⋆}^{\left(i\right)},Z_{i}\right)\right],$$

where the expectation is taken over a fresh sample $Z_{i}\sim {𝒟}_{i}$. At a high level, this learning paradigm of federated learning aims to obtain possibly different trained models for each client such that the individualized excess risks are low (see, e.g., Kairouz et al. 2019).

From a statistical viewpoint, perhaps the most crucial factor in determining the effectiveness of federated learning is *data heterogeneity*. When the data distribution ${𝒟}_{i}$ is (approximately) homogeneous across different clients, presumably a *single* global model would lead to small ${\text{IER}}_{i}$ for all i. In this regime, indeed, McMahan et al. (2017) proposed the *federated averaging* algorithm (FedAvg, see Algorithm 1), which can be regarded as an instance of local stochastic gradient descent (SGD) for solving (Mangasarian and Solodov, 1993; Stich, 2019)

(2) $$\underset{w}{\text{min}}\frac{1}{N}\sum_{i\in \left[m\right]}n_{i}L_{i}\left(w,S_{i}\right),$$

where $L_{i}\left(w,S_{i}\right)≔\sum_{j\in \left[n_{i}\right]}\ell \left(w,z_{j}^{\left(i\right)}\right)/n_{i}$ is the empirical risk minimization (ERM) objective of the i-th client and $N=n_{1}+\cdots +n_{m}$ denotes the total number of training samples. Translating Algorithm 1 into words, FedAvg in effect learns a shared global model using gradients from each client and outputs a single model as an estimate of $w_{⋆}^{\left(i\right)}$ for all clients. When the distributions $\left\{{𝒟}_{i}\right\}$ coincide with each other, FedAvg with a strongly convex loss achieves a weighted average excess risk of 𝒪(1/N), which is minimax optimal up to a constant factor (Shalev-Shwartz et al., 2009; Agarwal et al., 2012), see the formal statement in Theorem 6.

However, it is an entirely different story in the presence of data heterogeneity. FedAvg has been recognized to give inferior performance when there is a significant departure from complete homogeneity (see, e.g., Bonawitz et al. 2019). To better understand this point, consider the extreme case where the data distributions $\left\{{𝒟}_{i}\right\}$ are entirely unrelated. This roughly amounts to saying that the model parameters $\left\{w_{⋆}^{\left(i\right)}\right\}$ can be arbitrarily different from each other. In such a “completely heterogeneous” scenario, the objective function (2) simply has no clear interpretation, and any single global model—for example, the output of FedAvg—would lead to unbounded risks for most, if not all, clients. As a matter of fact, it is not difficult to see that the optimal training strategy for federated learning in this regime is arguably PureLocalTraining, which lets each client separately run SGD to minimize its own local ERM objective

(3) $$\underset{w^{\left(i\right)}}{\text{min }}L_{i}\left(w^{\left(i\right)},S_{i}\right)$$

without any communication. Indeed, PureLocalTraining is minimax rate optimal in the completely heterogeneous regime, just as FedAvg in the completely homogeneous regime (see Theorem 4).


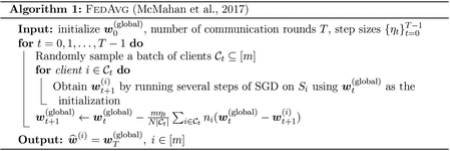


The level of data heterogeneity in practical federated learning problems is apparently neither complete homogeneity nor complete heterogeneity. Thus, the foregoing discussion raises a pressing question of what would happen if we are in the *wide* middle ground of the two extremes. This underlines the essence of *personalized federated learning*, which seeks to develop algorithms that perform well over a wide spectrum of data heterogeneity. Despite a venerable line of work on personalized federated learning (see, e.g., Kulkarni et al. 2020), the literature remains relatively silent on how the *fundamental* limits of personalized federated learning depend on data heterogeneity, as opposed to two extreme cases where both the minimax optimal rates and algorithms are known.

### 1.1 Main Contributions

The present paper takes a step toward understanding the statistical limits of personalized federated learning by establishing the minimax rates of convergence for both individualized excess risks and their weighted average with smooth strongly convex losses. We briefly summarize our main contributions below.

1. We prove that if the client-wise sample sizes are relatively balanced, then there exists a problem instance on which the ${\text{IER}}_{i}$’s of any algorithm are lower bounded by
(4) $$\left\{\begin{array}{ll} \Omega \left(1/N+R^{2}\right) & \text{if }R^{2}=𝒪(m/N)\\ \Omega (m/N) & \text{if }R^{2}=\Omega (m/N), \end{array}\right.$$
  where R is the minimum quantity satisfying ${\text{min}}_{w\in 𝒲}\sum_{i\in [m]}n_{i}{∥w_{⋆}^{\left(i\right)}-w∥}^{2}/N\leq R^{2}$, i.e., it measures the maximum level of heterogeneity among clients (here ‖⋅‖ throughout the paper denotes the Euclidean distance). Meanwhile, we show that the ${\text{IER}}_{i}$’s of FedAvg are upper bounded by $𝒪\left(1/N+R^{2}\right)$, whereas the guarantee for PureLocalTraining is 𝒪(m/N), regardless of the specific value of R. Moreover, we also establish similar upper and lower bounds for a weighted average of the ${\text{IER}}_{i}$’s under a weaker condition.
2. A closer look at the above-mentioned bounds reveals a perhaps surprising phenomenon: for a given collection of problem instances with a specified maximum level of heterogeneity, exactly one of FedAvg or PureLocalTraining is minimax optimal.
3. The established minimax results suggest that the naïve dichotomous strategy of (1) running FedAvg when $R^{2}=𝒪(m/N)$, and (2) running PureLocalTraining when $R^{2}=\Omega (m/N)$, attains the lower bound (4). Moreover, for supervised problems, this dichotomous strategy can be implemented without knowing R by (1) running both FedAvg and PureLocalTraining, (2) evaluating the test errors of the two algorithms in a distributed fashion, and (3) deploying the algorithm with a lower test error. We emphasize that the notion of optimality under our consideration overlooks constant factors. In practice, a better personalization result could be achieved by more sophisticated algorithms.
4. As a side product, we provide a novel analysis of FedProx, a popular algorithm for personalized federated learning that constrains the learned local models to be close via $\ell_{2}$ regularization (Li et al., 2018). In particular, we show that its ${\text{IER}}_{i}$’s are of order $𝒪\left(\frac{1}{N/m}\wedge \frac{R}{\sqrt{N/m}}+\frac{\sqrt{m}}{N}\right)$, and a weighted average of the ${\text{IER}}_{i}$’s satisfies a tighter $𝒪\left(\frac{1}{N/m}\wedge \frac{R}{\sqrt{N/m}}+\frac{1}{N}\right)$ bound, where a∧b=min{a,b} for two real numbers a and b.
5. On the technical side, our upper bound analysis is based on a generalized notion of algorithmic stability (Bousquet and Elisseeff, 2002), which we term *federated stability* and can be of independent interest. Briefly speaking, an algorithm $𝒜(S)=\left\{{\hat{w}}^{\left(i\right)}(S)\right\}$ has federated stability $\left\{\gamma_{i}\right\}$ if for any i∈[m], the loss function evaluated at ${\hat{w}}^{\left(i\right)}(S)$ can only change by an additive term of $𝒪\left(\gamma_{i}\right)$, if we perturb $S_{i}$ a little bit, while keeping the rest of datasets $\left\{S_{i^{\prime }}:i^{\prime }\neq i\right\}$ fixed. Similar ideas have appeared in Maurer (2005) and have been recently applied to multi-task learning (Wang et al., 2018). However, their notion of perturbation is based on the deletion of the whole client-wise dataset, whereas our notion of federated stability operates at the “record-level” and is more fine-grained. On the other hand, our construction of the lower bound is based on a generalization of Assound’s lemma (Assouad, 1983) (see also Yu 1997), which enables us to handle multiple heterogeneous datasets.

### 1.2 Related Work

Ever since the proposal of federated learning by McMahan et al. (2017), recent years have witnessed a rapidly growing line of work that is concerned with various aspects of FedAvg and its variants (see, e.g., Khaled et al. 2019; Haddadpour and Mahdavi 2019; Li et al. 2020b; Bayoumi et al. 2020; Malinovsky et al. 2020; Li and Richtárik 2020; Woodworth et al. 2020; Yuan and Ma 2020; Zheng et al. 2021).

In the context of personalized federated learning, there have been significant algorithmic developments in recent years. While the idea of using $\ell_{2}$ regularization to constrain the learned models to be similar has appeared in early works on multi-task learning (Evgeniou and Pontil, 2004), its applicability to personalized federated learning was only recently demonstrated by Li et al. (2018), where the FedProx algorithm was introduced. Similar regularization-based methods have been proposed and analyzed from the scope of convex optimization in Hanzely and Richtárik (2020); Dinh et al. (2020), and Hanzely et al. (2020). In particular, Hanzely et al. (2020) showed that an accelerated variant of FedProx is optimal in terms of communication complexity and the local oracle complexity. There is also a line of work using model-agnostic meta learning (Finn et al., 2017) to achieve personalization (Jiang et al., 2019; Fallah et al., 2020). Other strategies have been proposed (see, e.g., Arivazhagan et al. 2019; Li and Wang 2019; Mansour et al. 2020; Yu et al. 2020), and we refer readers to Kulkarni et al. (2020) for a comprehensive survey. We briefly remark here that all the papers mentioned above only consider the *optimization properties* of their proposed algorithms, while we focus on statistical properties of personalized federated learning.

Compared to the optimization understanding, our statistical understanding (in terms of sample complexity) of federated learning is still limited. Deng et al. (2020) proposed an algorithm for personalized federated learning with learning-theoretic guarantees. However, it is unclear how their bound scales with the heterogeneity among clients.

More generally, exploiting the information “shared among multiple learners” is a theme that constantly appears in other fields of machine learning such as multi-task learning (Caruana, 1997), meta learning (Baxter, 2000), and transfer learning (Pan and Yang, 2009), from which we borrow a lot of intuitions (see, e.g., Ben-David et al. 2006; Ben-David and Borbely 2008; Ben-David et al. 2010; Maurer et al. 2016; Cai and Wei 2019; Hanneke and Kpotufe 2019, 2020; Du et al. 2020; Tripuraneni et al. 2020a, b; Kalan et al. 2020; Shui et al. 2020; Li et al. 2020a; Zhang et al. 2020; Jose and Simeone 2021).

More related to our work, a series work by Denevi et al. (2018, 2019); Balcan et al. (2019), and Khodak et al. (2019) assumes the optimal local models lie in a small sub-parameter-space, and establishes “heterogeneity-aware” bounds on a weighted average of individualized excess risks. However, we would like to point out that they operate under the online learning setup, where the datasets are assumed to come in streams, and this is in sharp contrast to the federated learning setup, where the datasets are decentralized. Our notion of heterogeneity is also related to the hierarchical Bayesian model considered in Bai et al. (2020); Lucas et al. (2020); Konobeev et al. (2020), and Chen et al. (2020).

### 1.3 Paper Organization

The rest of this paper is organized as follows. In Section 2, we give an exposition of the problem setup and main assumptions. Section 3 presents our main results with proof sketches. We conclude this paper with a discussion of open problems in Section 4. For brevity, detailed proofs are deferred to the appendix.

## 2. Problem Setup

In this section, we detail some preliminaries to prepare the readers for our main results.

### Notation.

We introduce the notation we are going to use throughout this paper. For two real numbers a,b, we let a∨b=max{a,b} and a∧b=min{a,b}. For two non-negative sequences $a_{n},b_{n}$, we denote $a_{n}≲b_{n}$ (resp. $a_{n}≳b_{n}$) if $a_{n}\leq Cb_{n}$ (resp. $a_{n}\geq Cb_{n}$) for some constant C>0 when n is sufficiently large. We use $a_{n}≍b_{n}$ to indicate that $a_{n}≳b_{n},a_{b}≲b_{n}$ hold simultaneously. We also use $a_{n}=𝒪\left(b_{n}\right)$, whose meaning is the same as $a_{n}≲b_{n}$, and $a_{n}=\Omega \left(b_{n}\right)$, whose meaning is the same as $a_{n}≳b_{n}$. For two probability distributions ${𝒟}_{1}$ and ${𝒟}_{2}$, we use ${𝒟}_{1}⊗{𝒟}_{2}$ to denote their joint distribution under independence. We use 𝒲 to denote the parameter space and 𝒵 to denote the sample space. Finally, we let ${𝒫}_{𝒲}(x)≔{\text{argmin }}_{y\in 𝒲}\|x-y\|$ denote the operator that projects x onto 𝒲 in Euclidean distance.

### Evaluation Metrics.

The presentation of our main results relies on how to evaluate the performance of a federated learning algorithm. To this end, we consider the following two evaluation metrics.

**Definition 1 (Individualized excess risk)** *Consider an algorithm* 𝒜 *that outputs* $𝒜(S)={\left\{{\hat{w}}^{\left(i\right)}(S)\right\}}_{i=1}^{m}$*. For the* i-*the client, its individualized excess risk (IER) is defined as*

(5) $${\text{IER}}_{i}(𝒜)≔E_{Z_{i}\sim {𝒟}_{i}}\left[\ell \left({\hat{w}}^{\left(i\right)}(S),Z_{i}\right)-\ell \left(w_{⋆}^{\left(i\right)},Z_{i}\right)\right],$$

*where* $Z_{i}\sim {𝒟}_{i}$ *is a fresh data point independent of S*.

**Definition 2 (p-average excess risk)** *Consider an algorithm 𝒜 that outputs* $𝒜(S)=\left\{{\hat{w}}^{\left(i\right)}(S)\right\}$. *For a vector $p=\left(p_{1},\ldots ,p_{m}\right)$* lying in the m-dimensional probability simplex (i.e., all $p_{i}$’s *are non-negative and they sum to one), we define the p*-*average excess risk (*${\text{AER}}_{p}$*) of* 𝒜 *to be*

(6) $${\text{AER}}_{p}\left(𝒜\right)≔\underset{i\in \left[m\right]}{\sum }p_{i}\cdot {\text{IER}}_{i}\left(𝒜\right).$$

In words, IER measures the performance of the algorithm from the *client-wise* perspective, whereas AER evaluates the performance of the algorithm from the *system-wide* perspective.

Intuitively speaking, the weight vector p in (6) can be regarded as the importance weight on each client and controls “how many resources are allocated to each client”. For example, setting $p_{i}=1/m$ enforces “fair allocation”, so that each client is treated uniformly, regardless of sample sizes. As another example, setting $p_{i}=n_{i}/N$ (recall that $N=\sum_{i\in [m]}n_{i}$ is the total sample size) means that the central server pays more attention to clients with larger sample sizes, which, to a certain extend, incentivize the clients to contribute more data.

Notably, while a uniform upper bound on all ${\text{IER}}_{i}$’s can be carried over to the same bound on ${\text{AER}}_{p}$, a bound on the ${\text{AER}}_{p}$ alone in general does not imply a tight bound on each ${\text{IER}}_{i}$, other than the trivial bound ${\text{IER}}_{i}\leq {\text{AER}}_{p}/p_{i}$. Such a subtlety is a distinguishing feature of personalized federated learning in the following sense: under homogeneity, it suffices to estimate a single shared global model, and thus ${\text{AER}}_{p}$ and all of ${\text{IER}}_{i}\text{s}$ are mathematically equivalent.

### Regularity Conditions.

In this paper, we restrict ourselves to bounded, smooth, and strongly convex loss functions. Such assumptions are common in the federated learning literature (see, e.g., Li et al. 2020b; Hanzely et al. 2020) and cover many unsupervised learning problems such as mean estimation in exponential families and supervised learning problems such as generalized linear models.

**Assumption A (Regularity conditions)** *Suppose the following conditions hold*:

- Compact and convex domain. *The parameter space* 𝒲 *is a compact convex subset of* $R^{d}$ *with diameter* $D≔{\text{sup}}_{w,w^{\prime }\in 𝒟}∥w-w^{\prime }∥<\infty$;
- Smoothness and strong convexity. *For any* i∈[m], *the loss function* ℓ(⋅,z) *is* β-*smooth for almost every* z *in the support of* ${𝒟}_{i}$, *and the* i-*th ERM objective $L_{i}(\cdot ,S)$ is almost surely* μ-*strongly convex on the convex domain* $𝒲\subseteq R^{d}$. *We also assume that there exists a universal constant $\|\ell {\|}_{\infty }$ such that* $0\leq \ell (\cdot ,z)\leq \|\ell {\|}_{\infty }$ *for almost every* z *in the support of* ${𝒟}_{i}$;
- Bounded gradient variance at optimum. *There exists a positive constant* σ *such that for any* i∈[m], *we have* $E_{Z_{i}\sim {𝒟}_{i}}{∥\nabla \ell \left(w_{⋆}^{\left(i\right)},Z_{i}\right)∥}^{2}\leq \sigma^{2}$.

### Heterogeneity Conditions.

To quantify the level of heterogeneity among clients, we start by introducing the notion of an *average global model*. Assuming a strongly convex loss, the optimal local models (1) are uniquely defined. Thus, we can define the average global model as

(7) $$w_{p}^{\left(\text{global}\right)}=\underset{i\in \left[m\right]}{\sum }p_{i}w_{⋆}^{\left(i\right)}.$$

We remark that the average global model defined in (7) should not be interpreted as the “optimal global model”. Rather, it is more suitable to think of $w_{p}^{\text{(global) }}$ as a point in the parameter space, from which every local model is close to. Indeed, one can readily check that the average global model is the minimizer of $\sum_{i\in [m]}p_{i}{∥w_{⋆}^{\left(i\right)}-w∥}^{2}$ over $w\in R^{d}$.

We are now ready to quantify the level of client-wise heterogeneity as follows.

**Assumption B (Level of heterogeneity)** *There exists a positive constant* R *such that*

- *either* $\sum_{i\in [m]}p_{i}{∥w_{⋆}^{\left(i\right)}-w_{p}^{\left(\text{global}\right)}∥}^{2}\leq R^{2},$
- *or* ${∥w_{⋆}^{\left(i\right)}-w_{p}^{\left(\text{global}\right)}∥}^{2}\leq R^{2}\forall i\in [m]$.

Our study of the ${\text{AER}}_{p}$ and ${\text{IER}}_{i}\text{s}$ will be based on Part (a) and (b) of Assumption B, respectively. Intuitively, the quantity R encodes one’s belief on “how heterogeneous” the clients can be.

## 3. Main Results

### 3.1 Analyses of Two Baseline Algorithms

In this subsection, we characterize the performance of PureLocalTraining and FedAvg under the heterogeneity conditions imposed by Assumption B.

#### 3.1.1 Warm Up: Uniform Stability and Analysis of PureLocalTraining

The analysis of PureLocalTraining is based on the classical notion of uniform stability, proposed by Bousquet and Elisseeff (2002).

**Definition 3 (Uniform stability)** *Consider an algorithm* 𝒜 *that takes a single dataset* $S={\left\{z_{j}\right\}}_{j=1}^{n}$ *of size* n *as input and outputs a single model:* $𝒜(S)=\overset{ˆ}{w}(S)$. *We say* 𝒜 *is* γ-*uniformly stable if for any dataset* S*, any* j∈[n], *and any $z_{j}^{\prime }\in 𝒵$*, *we have*

$${∥\ell (\overset{ˆ}{w}(S),\cdot )-\ell \left(\overset{ˆ}{w}\left(S^{\setminus j}\right),\cdot \right)∥}_{\infty }\leq \gamma ,$$

*where* $S^{\setminus j}$ *is the dataset formed by replacing $z_{j}$ with* $z_{j}^{\prime }$:

$$S^{\setminus j}=\left\{z_{1},\ldots ,z_{j-1},z_{j}^{\prime },z_{j},\ldots ,z_{n}\right\}.$$

The main implication of uniformly stable algorithms is that “stable algorithms do not overfit”: if 𝒜 is γ-uniformly stable, then its *generalization error* is upper bounded by a constant multiple of γ. Thus, one can dissect the analysis of 𝒜 into two separate parts: (1) bounding its optimization error; (2) bounding its stability term.

Under our working assumptions, SGD with properly chosen step sizes is guaranteed to converge to the global minimum of (3) (see, e.g., Rakhlin et al. (2011)). Note that the bounds for the approximate minimizers only involve an extra additive term representing the optimization error, and this term will be negligible if we run SGD until convergence since our focus is sample complexity. Thus, we conduct the analysis for the global minimizer of (3). The performance of PureLocalTraining is given by the following theorem.

**Theorem 4 (Performance of PureLocalTraining)** *Let* Assumption A(b) *hold and assume* $n_{i}\geq 4\beta /\mu \forall i\in [m]$. *Then the algorithm* ${𝒜}_{\text{PLT }}$ *which outputs the minimizer of* (3) *satisfies*

$$E_{S}\left[{\text{IER}}_{i}\left({𝒜}_{\text{PLT}}\right)\right]≲\frac{\beta \|\ell {\|}_{\infty }}{\mu n_{i}}$$

*for all* i=1,…,m.

**Proof** The proof is a direct consequence of standard results on uniform stability of strongly convex ERM (see, e.g., Section 5 of Shalev-Shwartz et al. (2009) and Section 13 of Shalev-Shwartz and Ben-David (2014)), which assert that under the current assumptions, the minimizer of (3) is $𝒪\left(\frac{\beta \|\ell {\|}_{\infty }}{\mu n_{i}}\right)$-uniformly stable. We omit the details. ■

By definition, for any weight vector p, ${\text{AER}}_{p}$ of PureLocalTraining also admits the same upper bound as (4).

#### 3.1.2 Federated Stability and Analysis of FedAvg

We consider the following weighted version of (2):

(8) $$\underset{w\in 𝒲}{\text{min}}\underset{i\in \left[m\right]}{\sum }p_{i}L_{i}\left(w,S_{i}\right).$$

The FedAvg algorithm (Algorithm 1) also seamlessly generalizes. The above optimization formulation is in fact covered by the general theory of Li et al. (2020b), where they showed that FedAvg is guaranteed to converge to the global optimum under a suitable hyperparameter choice, even in the presence of heterogeneity (but the convergence is slower). Thus, in the following discussion, we again consider the global minimizer of (8).

It turns out that a tight analysis of FedAvg requires a more fine-grained notion of uniform stability, which we present below.

**Definition 5 (Federated stability)** *An algorithm* 𝒜 *that outputs* $𝒜(S)=\left\{{\hat{w}}^{\left(i\right)}(S)\right\}$ *has federated stability* ${\left\{\gamma_{i}\right\}}_{i=1}^{m}$ *if for every* $S\sim {⨂}_{i}{𝒟}_{i}^{⊗n_{i}}$ *and for any* $i\in [m],j_{i}\in \left[n_{i}\right],z_{i,j_{i}}^{\prime }\in 𝒵$, *we have*

$${∥\ell \left({\hat{w}}^{\left(i\right)}(S),\cdot \right)-\ell \left({\hat{w}}^{\left(i\right)}\left(S^{\setminus \left(i,j_{i}\right)}\right),\cdot \right)∥}_{\infty }\leq \gamma_{i}.$$

*Above*, $S^{\setminus \left(i,j_{i}\right)}$ *is the dataset formed by replacing $z_{j_{i}}^{\left(i\right)}$ in the* i-*the dataset with* $z_{i,j_{i}}^{\prime }$:

$$S^{\setminus \left(i,j_{i}\right)}=\left\{S_{1},\ldots ,S_{i-1},S_{i}^{\setminus j_{i}},S_{i+1},\ldots ,S_{m}\right\},$$

$$S_{i}^{\setminus j_{i}}=\left\{z_{1}^{\left(i\right)},\ldots ,z_{j_{i}-1}^{\left(i\right)},z_{i,j_{i}}^{\prime },z_{j_{i}+1}^{\left(i\right)},\ldots ,z_{n_{i}}^{\left(i\right)}\right\}.$$

Compared to the conventional uniform stability in Definition 3, federated stability provides a finer control by allowing distinct stability measures $\left\{\gamma_{i}\right\}$ for different clients. Moreover, the classical statement that “stable algorithms do not overfit” still holds, in the sense that the average (resp. individualized) generalization error can be upper bounded by $𝒪\left(\sum_{i\in [m]}n_{i}\gamma_{i}/N\right)$ (resp. $𝒪\left(\gamma_{i}\right)$), plus a term scaling with the level of heterogeneity R. And this again enables us to separate the analysis of 𝒜 into two parts (namely bounding the optimization error and bounding the stability), as is the case with the conventional uniform stability.

The notion of federated stability has other implications when restricted to the FedProx algorithm, and we refer the readers to Section 3.4 for details.

We are now ready to state the theorem that characterizes the performance of FedAvg.

**Theorem 6 (Performance of FedAvg)** *Let* Assumption A(b, c) *hold and assume* $n_{i}\geq 4\beta p_{i}/\mu \forall i\in [m]$. *Suppose the* FedAvg *algorithm ${𝒜}_{\text{FA}}$ outputs the minimizer of* (8). *Then under* Assumption B(a), *we have*

(9) $$E_{S}\left[{\text{AER}}_{p}\left({𝒜}_{\text{FA}}\right)\right]≲\frac{\beta \|\ell {\|}_{\infty }}{\mu }\underset{i\in \left[m\right]}{\sum }\frac{p_{i}^{2}}{n_{i}}+\beta R^{2},$$

*and under* Assumption B(b), *we have*

(10) $$E_{S}\left[{\text{IER}}_{i}\left({𝒜}_{\text{FA}}\right)\right]≲\frac{\beta \sigma^{2}}{\mu^{2}}\sum_{i^{\prime }\in \left[m\right]}\frac{p_{i^{\prime }}^{2}}{n_{i^{\prime }}}+\frac{\beta^{3}}{\mu^{2}}R^{2}.$$

**Proof** The proof of (9) is, roughly speaking, based on the fact that the global minimizer of (8) has federated stability $\gamma_{i}≲\frac{\beta \|\ell {\|}_{\infty }p_{i}}{\mu n_{i}}$, and thus the first term in the right-hand side of (9) corresponds to the average federated stability $\sum_{i\in [m]}p_{i}\gamma_{i}$. The second term $\beta R^{2}$ in the right-hand side of (9) reflects the presence of heterogeneity. For Equation (10), we were not able to obtain a federated stability based proof, and our current proof is based on an adaptation of the arguments in Theorem 7 of Foster et al. (2019), which explains why the dependence on (σ,β,μ) are different (and slightly worse) compared to Equation (9). In particular, the bound (10) has inverse quadratic dependence on μ, wheres the bound (9) only has 1/μ dependence. The 1/μ dependence comes from the fact that the federated stability term has such dependence, and the $1/\mu^{2}$ dependence comes from the fact that the $\ell_{2}$ estimation error has such dependence. We refer the readers to Appendix C.1 for details. ■

Note that both bounds in the above theorem are minimized by choosing $p_{i}=n_{i}/N$. With this choice of p, the two bounds read

(11) $$E_{S}\left[{\text{AER}}_{p}\left({𝒜}_{\text{FA}}\right)\right]≲\frac{\beta \|\ell {\|}_{\infty }}{\mu N}+\beta R^{2},\;E_{S}\left[{\text{IER}}_{i}\left({𝒜}_{\text{FA}}\right)\right]≲\frac{\beta \sigma^{2}}{\mu^{2}N}+\frac{\beta^{3}R^{2}}{\mu^{2}}.$$

This makes sense, since this choice of weight corresponds to the ERM objective under complete homogeneity. This observation also suggests that ensuring “fair resource allocation” (i.e., setting $p_{i}=1/m$) can lead to statistical inefficiency, especially when the sample sizes are imbalanced.

We conclude this subsection by noting that though the compactness assumption (Assumption A(a)) is not needed in Theorem 6, it is usually needed in the analysis of the optimization error of FedAvg and PureLocalTraining(see, e.g., Rakhlin et al. (2011); Li et al. (2020b)).

### 3.2 Lower Bounds

In this subsection, we present our construction of lower bounds, which characterize the information-theoretic limit of personalized federated learning. Throughout this section, we restrict out attention to the case where $p_{i}=n_{i}/N$ for any i∈[m].

Our construction starts by considering a special class of problem instances: logistic regression. In logistic regression, given the collection of regression coefficients $\left\{w_{⋆}^{\left(i\right)}\right\}\subseteq 𝒲$ where 𝒲 has a diameter D, the data distributions ${𝒟}_{i}$’s are supported on $R^{d}\times \{\pm 1\}$ and specified by a two-step procedure as follows:

1. Generate a feature vector x, whose coordinates are i.i.d. copies from some distribution $P_{X}$ on R, which is assumed to have mean zero and is almost surely bounded by some absolute constant $c_{X}$;
2. Generate the binary label y∈{±1}, which is a biased Rademacher random variable with head probability ${\left(1+\text{exp}\left\{-x^{⊤}w_{⋆}^{\left(i\right)}\right\}\right)}^{-1}$.

The loss function is naturally chosen to be the negative log-likelihood function, which takes the following form:

$$\ell (w,z)=\ell (w,x,y)=\text{log}\left(1+e^{-yx^{⊤}w}\right).$$

The following lemma says that Assumption A holds for the aforementioned logistic regression models.

**Lemma 7 (Logistic regressions are valid problem instances)** *The logistic regression problem described above is a class of problem instances that satisfies* Assumption A *with* $\|\ell {\|}_{\infty }=c_{X}D\sqrt{d}$ *and* $\sigma^{2}=\beta =c_{X}^{2}d/4$*. Moreover, if* $m≲(N/m{)}^{c}$ *for some* c≥0 *and* N/m≥Cd *for some* C>1, *then there exists some event ⋿ which only depends on the features* $\left\{x_{j}^{\left(i\right)}:i\in [m],j\in \left[n_{i}\right]\right\}$ *and happens with probability at least $1-e^{-𝒪(\sqrt{N/m})}$*, *such that on this event, the strongly convex constant in* Assumption A *satisfies*

(12) $$\mu ≍\mu_{0}={\left(\text{exp}\left\{c_{X}D\sqrt{d}/2\right\}+\text{exp}\left\{-c_{X}D\sqrt{d}\right\}\right)}^{-2}.$$

**Proof** The compactness of the domain and the boundedness of the loss function hold by construction. To verify the rest parts of Assumption A, with some algebra one finds that

(13) $$\nabla^{2}\ell \left(w,x,y\right)=\frac{xx^{⊤}\text{exp}\left\{yx^{⊤}w\right\}}{{\left(1+\text{exp}\left\{yx^{⊤}w\right\}\right)}^{2}}⪯\frac{1}{4}xx^{⊤},$$

where ⪯ is the Loewner order and the inequality holds because $x/(1+x{)}^{2}=1/\left(x^{-1/2}+\right.{\left.x^{1/2}\right)}^{2}\leq 1/4$ for x>0. Since the population gradient has mean zero at optimum, the gradient variance at optimum can be upper bounded by the trace of the expected Hessian matrix, which, by the above display, is further upper bounded by $c_{X}^{2}d/4$. Thus, we can take $\sigma^{2}=c_{X}^{2}d/4$ in Part (c). Another message of the above display is that we can set the smoothness constant in Part (b) to be $\beta =c_{X}^{2}d/4$.

The only subtlety that remains is to ensure each local loss function is μ-strongly convex. Note that since $x/(1+x{)}^{2}$ is decreasing from (0, 1) and is increasing from (1,∞), the right-hand side of (13) dominates $\mu_{0}xx^{⊤}$ in Loewner order, where $\mu_{0}$ is the right-hand side of (12). Thus, the local population losses $E_{(x,y)\sim {𝒟}_{i}}[\ell (\cdot ,x,y)]$ are all $\mu_{0}$-strongly convex.

Now, note that

$$\nabla^{2}L_{i}\left(w^{\left(i\right)},S_{i}\right)=\frac{1}{n_{i}}\sum_{j\in \left[n_{i}\right]}\frac{x_{j}^{\left(i\right)}{\left(x_{j}^{\left(i\right)}\right)}^{⊤}\text{exp}\left\{y_{j}^{\left(i\right)}\left\langle x_{j}^{\left(i\right)},w^{\left(i\right)}\right\rangle \right\}}{{\left(1+\text{exp}\left\{y_{j}^{\left(i\right)}\left\langle x_{j}^{\left(i\right)},w^{\left(i\right)}\right\rangle \right\}\right)}^{2}}⪰\mu_{0}\cdot \frac{1}{n_{i}}\sum_{j\in \left[n_{i}\right]}x_{j}^{\left(i\right)}{\left(x_{j}^{\left(i\right)}\right)}^{⊤}.$$

Invoking Theorem 5.39 of Vershynin (2010) along with a union bound over all clients, we conclude that for any i∈[m], the minimum eigenvalue of $\sum_{j\in \left[n_{i}\right]}x_{j}^{\left(i\right)}{\left(x_{j}^{\left(i\right)}\right)}^{⊤}$ is lower bounded by a constant multiple of $n_{i}-p≳n_{i}$ (this is the definition of the event ⋿) with probability at least $1-me^{-𝒪\left(n_{i}\right)}\geq 1-e^{-𝒪(\sqrt{N/m})}$, and the proof is concluded. ■

Note that in the proof of the above lemma, we have established the $\mu_{0}≍\mu$-strong convexity of the client-wise population losses. Hence, lower bounding the excess risks reduces to lower bounding the $\ell_{2}$ *estimation errors ${∥{\hat{w}}^{\left(i\right)}-w_{⋆}^{\left(i\right)}∥}^{2}$* of the estimators ${\hat{w}}^{\left(i\right)}$ for $w_{⋆}^{\left(i\right)}$. Such a reduction allows us to use powerful tools from information theory.

To this end, we introduce two parameter spaces, corresponding to Part (a) and (b) of Assumption B. Recalling $w_{p}^{\left(\text{global}\right)}=\sum_{i\in [m]}p_{i}w_{⋆}^{\left(i\right)}$, we define

$${𝒫}_{1}≔\left\{{\left\{w_{⋆}^{\left(i\right)}\right\}}_{i=1}^{m}\subseteq 𝒲:\sum_{i\in [m]}p_{i}{∥w_{⋆}^{\left(i\right)}-w_{p}^{\left(\text{global }\right)}∥}^{2}\leq R^{2}\right\},$$

$${𝒫}_{2}≔\left\{{\left\{w_{⋆}^{\left(i\right)}\right\}}_{i=1}^{m}\subseteq 𝒲:{∥w_{⋆}^{\left(i\right)}-w_{p}^{\left(\text{global }\right)}∥}^{2}\leq R^{2}\forall i\in [m]\right\}.$$

Note that ${𝒫}_{1}$ and ${𝒫}_{2}$ index all possible values of $\left\{w_{⋆}^{\left(i\right)}\right\}$ that can arise in the logistic regression models under Assumption B (a) and (b), respectively.

With the notations introduced so far, we are ready to state the main result of this subsection.

**Theorem 8 (Minimax lower bounds for estimation errors)** *Consider the logistic regression model described above. Suppose* $n_{i}≍n_{i^{\prime }}$ *for any* $i\neq i^{\prime }\in [m]$ *and assume* $p_{i}=n_{i}/N$ *for any* i∈[m]. *Then we have*

(14) $$\;\underset{\left\{{\hat{w}}^{\left(i\right)}\right\}}{\text{inf}}\underset{\left\{w_{⋆}^{\left(i\right)}\right\}\in {𝒫}_{1}}{\text{sup}}\frac{1}{N}\sum_{i\in [m]}n_{i}E_{S}{∥{\hat{w}}^{\left(i\right)}-w_{⋆}^{\left(i\right)}∥}^{2}≳\frac{d}{N/m}\wedge R^{2}+\frac{d}{N},$$

(15) $$\underset{{\hat{w}}^{\left(i\right)}}{\text{inf}}\underset{\left\{w_{⋆}^{\left(i\right)}\right\}\in {𝒫}_{2}}{\text{sup}}E_{S}{∥{\hat{w}}^{\left(i\right)}-w_{⋆}^{\left(i\right)}∥}^{2}≳\frac{d}{n_{i}}\wedge R^{2}+\frac{d}{N}$$

*for all* i∈[m], *where the infimum is taken over all possible ${\hat{w}}^{\left(i\right)}s$ that are measurable functions of the data* S.

**Proof** See Appendix A. ■

Note that both lower bounds in Theorem 8 are a superposition of two terms, and they correspond to two distinct steps in the proof.

The first step in our proof is to argue that the lower bound under complete homogeneity is in fact a valid lower bound under our working assumptions, which gives the Ω(d/N) term. This is reasonable, since estimation under complete homogeneity is, in many senses, an “easier” problem. The proof of the Ω(d/N) term is based on the classical Assouad’s method (Assouad, 1983).

The second step is to use a generalized version of Assouad’s method that allows us to deal with multiple heterogeneous datasets. In particular, we need to carefully choose the prior distributions over the parameter space based on the level of heterogeneity, which ultimately leads to the $\Omega \left(\frac{d}{N/m}\wedge R^{2}\right)$ term. Recall that in the vanilla version of Assouad’s method where there is only one parameter, say $w_{⋆}$, one can lower bounds the minimax risk by the Bayes risk, and the prior distribution is usually chosen to be $w_{⋆}=\delta v$, where v follows a uniform distribution over all d-dimensional binary vectors and δ is chosen so that the resulting hypothesis testing problem has large type-I plus type-II error. In our case where there are m parameters $\left\{w_{⋆}^{\left(i\right)}\right\}$, we need to consider a different prior of the following form:

$$w_{⋆}^{\left(i\right)}=\delta_{i}v^{\left(i\right)},$$

where $v^{\left(i\right)}$ are i.i.d. samples from the uniform distribution over all d-dimensional binary vectors, and $\delta_{i}$’s are scalers that need to be carefully chosen to make the resulting hypothesis testing problem hard.

The following result is an immediate corollary of Theorem 8.

**Corollary 9 (Minimax lower bounds for excess errors)** *Assume there exist constants* $C,C^{\prime }>0,c\geq 0$ *such that* $n_{i}\geq C\beta \forall i\in [m]$ *and* $m\leq C^{\prime }(N/m{)}^{c}$. *Moreover, assume* $n_{i}≍n_{i^{\prime }}$ *for any* $i\neq i^{\prime }\in [m]$ *and* $p_{i}=n_{i}/N$ *for any* i∈[m]. *Then there exists an absolute constant* $c^{\prime }$ *such that the following two statements hold*:

*1*. *There exists a problem instance such that* Assumptions A *and* B(a) *are satisfied with probability at least $1-e^{-c^{\prime }\sqrt{N/m}}$*. *Call this high probability event* ⋿*. On this problem instance, any randomized algorithm* 𝒜 *must suffer*

(16) $$E_{𝒜,S}\left[{\text{AER}}_{p}(𝒜)\cdot 1_{\text{E}}\right]≳\mu \cdot \left(\frac{\beta }{N/m}\wedge R^{2}+\frac{\beta }{N}\right);$$

*2*. *For any* i∈[m]*, there exists a problem instance such that* Assumptions A *and* B(b) *are satisfied with probability at least $1-e^{-c^{\prime }\sqrt{N/m}}$*. *Call this high probability event ${\text{E}}_{i}$. On this problem instance, any randomized algorithm* 𝒜 *must suffer*

(17) $$E_{𝒜,S}\left[{\text{IER}}_{i}(𝒜)\cdot 1_{{\text{E}}_{i}}\right]≳\mu \cdot \left(\frac{\beta }{n_{i}}\wedge R^{2}+\frac{\beta }{N}\right).$$

*In the two displays above, the expectation is taken over the randomness in both the algorithm* 𝒜 *and the sample* S.

**Proof** Along with Lemma 7 and Theorem 8, this corollary follows by the fact that the smoothness constant β is of the same order as d and the population losses are all $\mu_{0}≍\mu$-strongly convex. ■

### 3.3 Implications of the Main Results

The upper bounds in Section 3.1 and the lower bounds in Section 3.2 together reveal several intriguing phenomena regarding personalized FL, which we detail in this subsection.

Focusing on the dependence on the sample sizes and assuming the client-wise samples sizes are balanced (i.e., $n_{i}≍N/m$), the heterogeneity measure R enters the lower and upper bounds in a dichotomous fashion:

- If $R^{2}≲m/N$, then both lower bounds become $\Omega \left(R^{2}+1/N\right)$, and this lower bound can be attained by FedAvg up to factors that do not depend on the sample sizes;
- If $R^{2}≳m/N$, then both lower bounds become Ω(m/N). They agree with the minimax rate as if we were under complete heterogeneity and can be achieved by PureLocalTraining.

Now, let us consider the following naïve dichotomous strategy: if output $R^{2}\leq \frac{\|\ell {\|}_{\infty }}{\mu }.\;m/N$, then output $𝒜={𝒜}_{\text{FA}}$; otherwise, output $𝒜={𝒜}_{\text{PLT}}$. That is, we switch between the two baseline algorithms at the threshold of $R^{2}≍m/N$. Then under the assumptions in Theorems 4 and 6, one can readily check that this dichotomous strategy satisfies the following AER guarantee:

(18) $$E_{S}\left[{\text{AER}}_{p}(𝒜)\right]≲\beta \left(\frac{\|\ell {\|}_{\infty }}{\mu N/m}\wedge R^{2}\right)+\frac{\beta \|\ell {\|}_{\infty }}{\mu }\sum_{i\in [m]}\frac{p_{i}^{2}}{n_{i}}.$$

If in addition, $n_{i}≍n_{i^{\prime }}$ for any $i\neq i^{\prime }\in [m]$, then it also satisfies the following IER guarantee:

(19) $$E_{S}\left[{\text{IER}}_{i}(𝒜)\right]≲\frac{\beta^{3}}{\mu^{2}}\left(\frac{\|\ell {\|}_{\infty }}{\mu n_{i}}\wedge R^{2}\right)+\frac{\beta \sigma^{2}}{\mu^{2}}\sum_{i^{\prime }\in \left[m\right]}\frac{p_{i^{\prime }}^{2}}{n_{i^{\prime }}}.$$

When $p_{i}=n_{i}/N$, the two displays above simplify to

$$E_{S}[\text{AER}(𝒜)]≲\beta \left(\frac{\|\ell {\|}_{\infty }}{\mu N/m}\wedge R^{2}\right)+\frac{\beta \|\ell {\|}_{\infty }}{\mu N},\;E_{S}\left[{\text{IER}}_{i}(𝒜)\right]≲\frac{\beta^{3}}{\mu^{2}}\left(\frac{\|\ell {\|}_{\infty }}{\mu n_{i}}\wedge R^{2}\right)+\frac{\beta \sigma^{2}}{\mu^{2}N},$$

which matches the lower bound in Corollary 9 up to constant factors, provided $\left(\beta ,\mu ,\|\ell {\|}_{\infty },\sigma \right)$ are all of constant order. In other words, switching between the two algorithms at the threshold of $R^{2}≍m/N$ gives an oracle algorithm that is minimax rate optimal.

Thus, we have shown an interesting property for personalized FL on the choice of the two baseline algorithms. In particular, consider a collection of problem instances indexed by (R,β,μ,‖ℓ‖,σ) using Assumptions A and B and assume $\left(\beta ,\mu ,\|\ell {\|}_{\infty },\sigma \right)$ are all of constant order. Now, for a fixed value of R, *exactly one of these two algorithms is minimax optimal*, where the optimality is defined over the specified collection of problem instances and with respect to both AER and IER. Moreover, the oracle dichotomous strategy that switches between the two baseline algorithms at the threshold of $R^{2}≍m/N$ is minimax optimal.

More implications of the theoretical results are described below.

#### Optimality of a dichotomous strategy.

From the practical side, for supervised learning problems, such a dichotomous strategy can be implemented without prior knowledge of R if test errors can be evaluated in a distributed fashion. Indeed, we can first run both FedAvg and PureLocalTraining separately, evaluate their test errors (in a distributed fashion), and deploy the one with a lower test error. Due to the upper and lower bounds proved in Sections 3.1 and 3.2, such a strategy is guaranteed to be minimax rate optimal. As a caveat, however, one should refrain from interpreting our results as saying either of the two baseline algorithms is sufficient for practical problems. From a practical viewpoint, constants that are omitted in the minimax analysis are crucial. Even for supervised problems, a better personalization result could be achieved by more sophisticated algorithms in practice. Nevertheless, our results suggest that the two baseline algorithms can at least serve as a good starting point in the search for efficient personalized algorithms.

For unsupervised problems where the quality of a model is hard to evaluate, implementing the dichotomous strategy requires estimating an upper bound R of the level of heterogeneity. This is an important open problem, which we leave for future work.

#### Optimality of FedAvg followed by local fine tuning.

Another popular baseline algorithm for personalized FL is to first run FedAvg until convergence, and then let each client run PureLocalTraining to fine tune the model. In strongly convex problems, global optima can be reached by gradient descent regardless the initialization with a suitable choice of the learning rate (see, e.g., Theorem 2.1.15 of Nesterov 2018). Thus, if each client run PureLocalTraining for long enough, the global optima for its local loss function will finally be reached. This fact tells that along the whole fine tuning trajectory, there is a point at which the model gives the worst-case optimal AER and IER, and for a fixed level of heterogeneity, this point is either at the very beginning (which is FedAvg), or at the very end (which is PureLocalTraining). Although this conclusion is almost trivial from a technical point of view given our minimax results, it provides a reassuring theoretical property (of being minimax optimal) for a popular method used by practitioners.

#### Illustrating the minimaxity in a simulated example.

**Figure 1:**
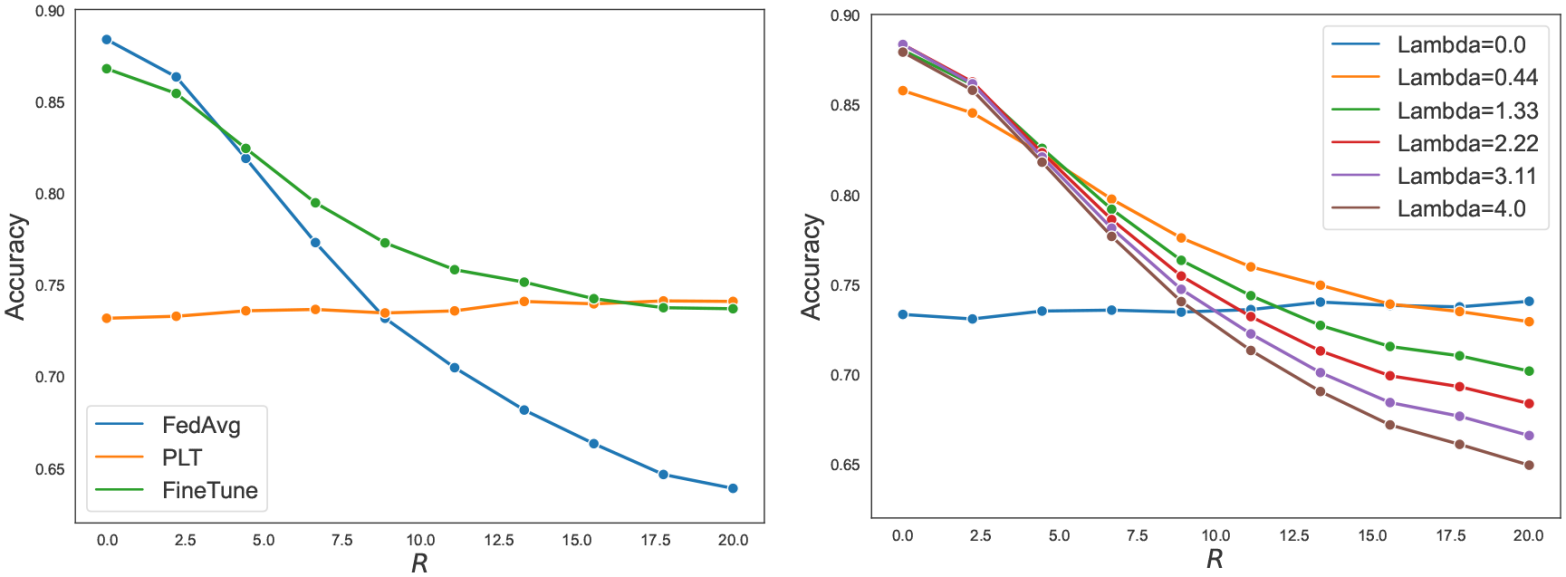
Average classification accuracy of FedAvg, PureLocalTraining and FedAvg followed by fine tuning (left panel) as well as FedProx with different choice of λ (right panel).

We conduct a simulation on federated logistic regression to corroborate our theoretical results and the optimality of the FedAvg following by local fine tuning strategy. In the simulation, we set $m=5,n_{i}=100,\forall i\in [m]$ and we vary R from 0 to 20 (see Appendix D for details). In the left panel of Figure 1, we plot the test accuracy (averaged over 100 rounds of simulations) of those three methods against the value of R. One can see that the accuracy of the fine tuning strategy roughly follows the maximum of the accuracies achieved by FedAvg and PureLocalTraining, confirming our theoretical prediction that the fine tuning strategy can indeed perform as well as the best between FedAvg and PureLocalTraining.

#### Beyond the current heterogeneity assumption.

Our minimax results are established under Assumption B, which states that all optimal local models are close to a certain “centroid” (i.e., the average global model defined in (7)). If we draw a graph of clients and connect two clients if their optimal local models are similar, then the current heterogeneity assumption gives rise to a complete graph (or a star-shaped graph if we introduce another node to represent the average global model). While such an idealized graph structure enables a clean theoretical analysis, the real world proximity patterns among clients are clearly far more sophisticated. In fact, counterexamples exist under which the minimax results does not hold in a “global” sense.

Suppose all m clients exhibit a clustering structure as follows. We have $\sqrt{m}$ clients whose optimal global models serve as cluster centroids, and those centroids are very far apart. In the neighborhood of each centroid, there are $\sqrt{m}$ clients whose optimal local models are $R_{c}$ away from the centroid in the sense of Assumption B. Additionally, assume each client has equal sample sizes, so that $n_{i}=n$ for some n. Under this setting, the “global” heterogeneity parameter R in Assumption B is very large, so our theory would suggest choosing PureLocalTraining, which gives a rate of 𝒪(1/n). However, this rate is clearly suboptimal. If one can successfully cluster the m clients into $\sqrt{m}$ clusters (which is hopeful as the centroids are assumed to be far apart), then one can apply our theory to each cluster (i.e., run the dichotomous strategy for each cluster) and conclude that the rate for each cluster is $𝒪\left(\frac{1}{n\sqrt{m}}+\frac{1}{n}\wedge R_{c}^{2}\right)\ll \frac{1}{n}$ if m diverges to infinity and $R_{c}\ll 1/\sqrt{n}$. The foregoing discussion reveals in such a clustered setting, our theoretical results only make sense at the cluster level, but not at the global level.

The behaviors of the minimax rate for more general client proximity graphs can be even more complicated, which we leave for future work.

### 3.4 More Implications of Federated Stability and Analysis of FedProx

In this subsection, we are concerned with the performance guarantees for FedProx. As with our earlier analysis of FedAvg, we consider a p-weighted version of FedProx, whose optimization formulation is given below:

(20) $$\underset{\begin{array}{c} w^{\left(\text{global}\right)}\in 𝒲\\ {\left\{w^{\left(i\right)}\right\}}_{i=1}^{m}\subseteq 𝒲 \end{array}}{\text{min}}\sum_{i\in [m]}p_{i}\left(L_{i}\left(w^{\left(i\right)},S_{i}\right)+\frac{\lambda }{2}{∥w^{\left(\text{global}\right)}-w^{\left(i\right)}∥}^{2}\right),$$

where we recall that $L_{i}\left(w,S_{i}\right)≔\sum_{j\in \left[n_{i}\right]}\ell \left(w,z_{j}^{\left(i\right)}\right)/n_{i}$ is the ERM objective for the i-th client. In this subsection, we let $\left({\tilde{w}}^{\text{(global)}},\left\{{\tilde{w}}^{\left(i\right)}\right\}\right)$ be the global minimizer of the above problem. Compared to (8), which imposes a “hard” constraint $w^{\left(i\right)}=w^{\left(\text{global}\right)}$, and compared to (3), where there is no constraint at all, the above formulation imposes a “soft” constraint that the norm of $\left(w^{\text{(global)}}-w^{\left(i\right)}\right)$ should be small, with a hyperparameter λ controlling the strength of this constraint.

The rationale behind the optimization formulation (20) of FedProx is clear: by setting λ=0, the optimization formulation of PureLocalTraining (3) is recovered, and as λ→∞, the optimization formulation of FedAvg (8) is recovered. The hope is that by varying λ∈(0,∞), one can interpolate between the two extremes.

Applying the idea of local SGD to (20), one obtains the FedProx algorithm^1^, which we detail in Algorithm 2. We separate the whole algorithm into two stages as they has distinct interpretations: in Stage I, the central server aims to learn a good global model with the help of local clients, whereas in Stage II, each local client takes advantage of the global model to personalize. Alternatively, one can also interpret FedProx as an instance of the general framework of *model-agnostic meta learning* (Finn et al., 2017), where Stage I learns a good initialization, and Stage II trains the local models starting from this initialization.

In contrast to our analyses for FedAvg and PureLocalTraining in Section 3.1, where we largely focused on global minimizers, the analysis for FedProx will be carried out for the approximate minimizer output by Algorithm 2. The reason for this is rooted in the tradeoff between the optimization error and the generalization error. Note that given the results derived in Section 3.1, the analysis for the global minimizer $\left({\tilde{w}}^{\left(\text{global}\right)},\left\{{\tilde{w}}^{\left(i\right)}\right\}\right)$ becomes trivial: by setting λ=0, we reduce the task to analyzing PureLocalTraining; by sending λ→∞, we reduce the task to analyzing FedAvg. Based on Theorems 4 and 6, one immediately concludes that there exists a choice of λ, such that the AER and IER of $\left\{{\tilde{w}}^{\left(i\right)}\right\}$ satisfy the bounds in (18) and (19), respectively. However, the foregoing discussion is purely restricted to generalization error. When we set λ=0 or send λ→∞, it is not known a priori whether FedProx algorithm will converge to the global minima. Worse still, the optimization error may depends on λ in a particular way so that it becomes unbounded when λ approaches zero or infinity. To the best of our knowledge, prior work only proved the optimization convergence of FedProx for the global model with a fixed value of λ, namely the convergence of $w_{T}^{\text{(global)}}$ to ${\tilde{w}}^{\text{(global)}}$ as the number of global communication rounds T tends to infinity (Li et al., 2018; Dinh et al., 2020). To have a theoretical understanding of the performance of FedProx, it is crucial to (1) establish the optimization convergence for both global and local models; (2) bound the generalization error; and (3) balance the optimization error and the generalization error, both of which are functions of λ. In the following, we execute the those steps with the aid of federated stability.


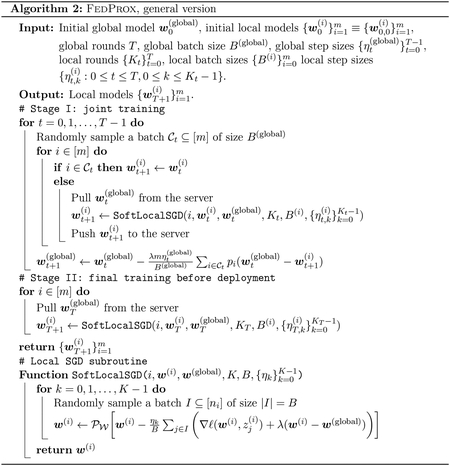


#### Implications of federated stability for FedProx.

We have briefly mentioned the main implications of federated stability in Section 3.1.2: for an algorithm $𝒜=\left\{{\hat{w}}^{\left(i\right)}\right\}$ with federated stability $\left\{\gamma_{i}\right\}$, its average generalization error (resp. individualized generalization error) can be upper bounded by $𝒪\left(\sum_{i\in [m]}p_{i}\gamma_{i}\right)$ (resp. $𝒪\left(\gamma_{i}\right)$), plus a term scaling with the level of heterogeneity R. We make such a statement precise here. Let us first define the optimization error of a generic algorithm $𝒜=\left({\hat{w}}^{\left(\text{global}\right)},\left\{{\hat{w}}^{\left(i\right)}\right\}\right)$ (which tries to solve (20)) as

$${ℰ}_{\text{OPT}}≔\sum_{i\in [m]}p_{i}\left(L_{i}\left({\hat{w}}^{\left(i\right)},S_{i}\right)+\frac{\lambda }{2}{∥{\hat{w}}^{\left(\text{global }\right)}-{\hat{w}}^{\left(i\right)}∥}^{2}\right)-\sum_{i\in [m]}p_{i}\left(L_{i}\left({\tilde{w}}^{\left(i\right)},S_{i}\right)+\frac{\lambda }{2}{∥{\tilde{w}}^{\left(\text{global }\right)}-{\tilde{w}}^{\left(i\right)}∥}^{2}\right).$$

The main implications of federated stability, when applied to the specifics of FedProx, can then be summarized in the following proposition.

**Proposition 10 (Implications of federated stability restricted to FedProx)** *Consider an algorithm* $𝒜=\left({\hat{w}}^{\text{(global)}},\left\{{\hat{w}}^{\left(i\right)}\right\}\right)$ *with federated uniform stability ${\left\{\gamma_{i}\right\}}_{1}^{m}$*. *Then we have*

(21) $$E_{𝒜,S}\left[{\text{AER}}_{p}(𝒜)\right]\;\leq E_{𝒜,S}\left[{ℰ}_{\text{OPT}}\right]+2\sum_{i\in [m]}p_{i}E_{𝒜,S}\left[\gamma_{i}\right]+\frac{\lambda }{2}\sum_{i\in [m]}p_{i}{∥w_{\text{avg}}^{\left(\text{global}\right)}-w_{⋆}^{\left(i\right)}∥}^{2},$$

(22) $$E_{𝒜,S}\left[{\text{IER}}_{i}(𝒜)\right]\leq \frac{E_{𝒜,S}\left[{ℰ}_{\text{OPT}}\right]}{p_{i}}+2E_{𝒜,S}\left[\gamma_{i}\right]+\frac{\lambda }{2}E_{𝒜,S}{∥{\hat{w}}^{\left(\text{global}\right)}-w_{⋆}^{\left(i\right)}∥}^{2}\;\forall i\in [m].$$

**Proof** The proof of (21) is based on the following basic inequality for the AER:

(23) $$\sum_{i\in [m]}p_{i}\left(L_{i}\left({\tilde{w}}^{\left(i\right)},S_{i}\right)+\frac{\lambda }{2}{∥{\tilde{w}}^{\left(\text{global}\right)}-{\tilde{w}}^{\left(i\right)}∥}^{2}\right)\leq \sum_{i\in [m]}p_{i}\left(L_{i}\left(w_{⋆}^{\left(i\right)},S_{i}\right)+\frac{\lambda }{2}{∥w_{\text{avg}}^{\left(\text{global}\right)}-w_{⋆}^{\left(i\right)}∥}^{2}\right),$$

whereas the proof of (22) is based on the following basic inequality for the IER: for any s∈[m], we have

(24) $$\sum_{i\in [m]}p_{i}\left(L_{i}\left({\tilde{w}}^{\left(i\right)},S_{i}\right)+\frac{\lambda }{2}{∥{\tilde{w}}^{\left(\text{global}\right)}-{\tilde{w}}^{\left(i\right)}∥}^{2}\right)\leq p_{s}L_{s}\left(w_{⋆}^{\left(s\right)},S_{s}\right)+\frac{\lambda }{2}{∥{\hat{w}}^{\left(\text{global}\right)}-w_{⋆}^{\left(s\right)}∥}^{2}+\sum_{i\neq s}p_{i}\left(L_{i}\left({\hat{w}}^{\left(i\right)},S_{i}\right)+\frac{\lambda }{2}{∥{\hat{w}}^{\left(\text{global}\right)}-{\hat{w}}^{\left(i\right)}∥}^{2}\right).$$

We refer the readers to Appendix C.2 for details. ■

Note that both bounds in Proposition 10 involve a term that scales linearly with both λ and the heterogeneity measure. In general, we expect the stability measures to scale inversely with λ, and thus opening the possibility of carefully choosing λ to balance the stability term and the heterogeneity term.

Let us observe that the heterogeneity term of (22) is slightly different than that of (21), in that it involves the estimated global model ${\hat{w}}^{\text{(global)}}$. This suggests that achieving the IER guarantees might be intrinsically more difficult than achieving the AER guarantees.

In view of Proposition 10, we are left to bound the optimization error and the federated stability of FedProx. As discussed above, achieving the AER and IER guarantees requires somewhat different assumptions, as the latter involves characterizing the performance of the global model. So we split our discussion into two parts below.

#### Bounding the average excess error.

The following theorem characterize the performance of FedProx in terms of the AER.

**Theorem 11 (AER guarantees for FedProx)** Let Assumptions A *and* B(a) *hold, and assume $n_{i}\geq 4\beta /\mu$ for all* i∈[m]. *Choose the weight vector p such that*

(25) $$\frac{p_{\text{max}}\sum_{i\in [m]}p_{i}/n_{i}}{\sum_{i\in [m]}p_{i}^{2}/n_{i}}\leq C_{p}$$

*for some constant $C_{p}$*, *where* $p_{\text{max}}={\text{max}}_{i}\;p_{i}$. *Consider the* FedProx *algorithm*, ${𝒜}_{\text{FP}}$, *with the following hyperparameter configuration*:

1. *In the joint training stage (i.e*., 0≤t≤T-1), *set*
(26) $$\eta_{t,k}^{\left(i\right)}=\frac{1}{\left(\mu +\lambda )(k+1\right)},\;\eta_{t}^{\left(\text{global}\right)}=\frac{2(\mu +\lambda )}{\lambda \mu (t+1)},\;K_{t}+1\geq C_{1}\left(\lambda^{2}\vee 1\right)t,\;T\geq C_{2}\lambda (\lambda \vee 1)m\|p{\|}^{2}\cdot \left({\left[\sum_{i\in [m]}p_{i}/n_{i}\right]}^{-1}\vee \left[\lambda (\lambda \vee 1)n_{\text{max}}^{2}\right]\right);$$
2. *In the final training stage (i.e*., t=T), *set*
(27) $$\eta_{T,k}^{\left(i\right)}=\frac{1}{\left(\mu +\lambda )(k+1\right)},\;K_{T}\geq C_{3}(\lambda +1{)}^{2}\cdot \left({\left[\sum_{i\in \left[m\right]}\frac{p_{i}}{n_{i}}\right]}^{-1}\vee \left[\lambda^{2}\underset{i\in \left[m\right]}{\text{max}}{\left(p_{i}n_{i}\right)}^{2}\right]\right),$$

*where* $C_{1},C_{2},C_{3}$ *are constants depending only on* $\left(\mu ,\beta ,\|\ell {\|}_{\infty },D\right)$. *Then, there exists a choice of* λ *such that*

(28) $$E_{{𝒜}_{\text{FP}},S}\left[{\text{AER}}_{p}\left({𝒜}_{\text{FP}}\right)\right]≲\left(\frac{\mu }{1\wedge C_{p}}+\frac{\left(1\vee C_{p}\right)\beta \|\ell {\|}_{\infty }}{\mu }\right)\left[\left(R\sqrt{\sum_{i\in \left[m\right]}\frac{p_{i}}{n_{i}}}\right)\wedge \left(\sum_{i\in \left[m\right]}\frac{p_{i}}{n_{i}}\right)+\sum_{i\in \left[m\right]}\frac{p_{i}^{2}}{n_{i}}\right].$$

**Proof** See Appendix C.3. ■

A few remarks are in order. First, (25) essentially says that the weight p cannot be too imbalanced, and too much imbalance in p can hurt the performance in view of the multiplicative factor of $C_{p}$ in our bound (28). If we set $p_{i}=1/m$, then $C_{p}$ is naturally of constant order; whereas if we set $p_{i}=n_{i}/N$, we have $C_{p}≍mn_{\text{max}}/N$, where $n_{\text{max}}={\text{max}}_{i}n_{i}$, which calls for relative balance of the sample sizes.

We then briefly comment on the hyperparameter choice in the above theorem. The step sizes are of the form 1/(strongly convex constant × iteration counter), and such a choice is common in strongly convex stochastic optimization problems (see, e.g., Rakhlin et al. 2011; Shamir and Zhang 2013). Such a choice, along with the smoothness of the problem, is also the key for us to by-pass the need of doing any time-averaging operation, as is done in, for example, Dinh et al. (2020).

In Theorem 11, the choice of the communication rounds T and the final local training round $K_{T}$ both scale polynomially with λ, which means that the optimization convergence of FedProx is slower when the data are less heterogeneous. This phenomenon happens more generally. For example, in Hanzely and Richtárik (2020), they proposed a variant of SGD that optimizes (20) with $p_{i}=1/m$ in $𝒪\left(\frac{L+\lambda }{\mu }\text{log}1/\varepsilon \right)$-many iterations, where L is the Lipschitz constant of the loss function and ε is the desired accuracy level.

The constants $C_{1},C_{2},C_{3}$ in the statement of Theorem 11 can be explicitly traced in our proof. We remark that the dependence on problem-specific constants $\left(\mu ,\beta ,\|\ell {\|}_{\infty },D\right)$ in our hyperparameter choice and on λ may not be tight. A tight analysis of the optimization error is interesting, but less relevant for our purpose of understanding the sample complexity. So we defer such an analysis to future work^2^.

#### Bounding individualized excess errors.

The following theorem gives the IER guarantees for FedProx.

**Theorem 12 (IER guarantees for FedProx)** *Let* Assumptions A *and* B(b) *hold. Moreover, assume that* $n_{i}≍n_{i^{\prime }}$ *for any* $i\neq i^{\prime }\in [m]$ *and* $n_{i}\geq 4\beta /\mu \forall i\in [m]$. *Let the weight vector be chosen as $p_{i}≍1/m\forall i\in [m]$*. *Consider the* FedProx *algorithm, ${𝒜}_{\text{FP}}$*, *with the following hyperparameter configuration*:

1. *In the joint training stage (i.e*., 0≤t≤T-1), *set* $\eta_{t,k}^{\left(i\right)},\eta_{t}^{\text{(global)}},K_{t}$ *as in* (26), *and set*
$$T\geq C_{2}^{\prime }\lambda (\lambda \vee 1)\underset{i\in [m]}{\text{max }}n_{i}\cdot \left(p_{i}^{-1}\vee \left[\lambda (\lambda \vee 1)n_{i}\right]\right);$$
2. *In the final training stage (i.e*., t=T), *set* $\eta_{T,k}^{\left(i\right)}$ *as in* (27), *and set*
$$K_{T}\geq C_{3}^{\prime }(\lambda +1{)}^{2}\underset{i\in [m]}{\text{max }}n_{i}\left(p_{i}^{-1}\vee \lambda^{2}p_{i}^{2}n_{i}\right),$$

*where* $C_{2}^{\prime },C_{3}^{\prime }$ *are constants only depending on $\left(\mu ,\beta ,\|\ell {\|}_{\infty },D\right)$. Then, there exists a choice of* λ *such that for any* i∈[m], *we have*

(29) $$E_{{𝒜}_{\text{FP}},S}\left[{\text{IER}}_{i}\left({𝒜}_{\text{FP}}\right)\right]≲\left[\left(\mu +\mu^{-1}\right)\left(\beta \|\ell {\|}_{\infty }+\frac{\sigma^{2}\beta^{2}+\beta^{2}+\sigma^{2}}{\mu^{2}}\right)+\mu D^{2}\right]\cdot \left(\frac{R}{\sqrt{n_{i}}}\wedge \frac{1}{n_{i}}+\frac{\sqrt{m}}{N}\right).$$

**Proof** See Appendix C.4. ■

Compared to Theorem 11, the above theorem imposes extra assumptions that the sample sizes are relative balanced and that $p_{i}≍1/m$, both of which are due to the fact that we need to additionally take care of the estimation error of the global model. The hyperparameter choice slightly differs from that in Theorem 11 for the same reason. In practice, when one is to use FedProx to optimize highly non-convex functions like the loss function of deep neural networks, instead of sticking to the choices made in Theorems 11 and 12, the hyperparameters are usually tuned by trial-and-error for best test performance.

#### Comparison with the lower bounds.

In order to comment about the optimality/suboptimality of FedProx, let us restrict to the case when $p_{i}=n_{i}/N$. In this case, the bound in Theorem 11 becomes

(30) $$E_{{𝒜}_{\text{FP}},S}\left[{\text{AER}}_{p}\left({𝒜}_{\text{FP}}\right)\right]≲\left(\mu +\frac{\beta \|\ell {\|}_{\infty }}{\mu }\right)\cdot \left(\frac{1}{N/m}\wedge \frac{R}{\sqrt{N/m}}+\frac{1}{N}\right).$$

Recall the lower bound in (16). Focusing on the dependence of sample sizes and heterogeneity measure, we have the following three cases. If $R^{2}≳m/N$, then (30) becomes 𝒪(m/N), which matches the lower bound. Meanwhile, if $1/mN≲R^{2}≲m/N$, then (30) becomes 𝒪(m/N), whereas the lower bound reads $\Omega \left(R^{2}+1/N\right)$, and thus (30) is suboptimal unless $R^{2}≍m/N$. Moreover, if $R^{2}≲1/mN$, then (30) becomes 𝒪(1/N), and is minimax optimal again.

A similar trilogy holds for IER of FedProx. Comparing the upper bound in (29) and the lower bound in (17), we still have three cases as follows. If $R^{2}≳m/N$, then (29) is $𝒪\left(1/n_{i}\right)$, which agrees with the lower bound. Meanwhile, if $1/N≲R^{2}≲m/N$, then (29) is $𝒪\left(R/\sqrt{n_{i}}\right)$, and is suboptimal compared to the $\Omega \left(R^{2}+1/N\right)$ lower bound unless $R^{2}≍m/N$. Moreover, if $R^{2}≲1/N$, then (29) is $𝒪(\sqrt{m}/N)$, and is off by a factor of order $\sqrt{m}$ compared to the Ω(1/N) lower bound.

While the bounds in Theorems 11 and 12 in general do not attain the lower bounds in Corollary 9, they are still non-trivial in the sense that they scale with the heterogeneity measure R. While there are some recent works establishing the AER guarantees for an objective similar to (20) under the online learning setup (see, e.g., Denevi et al. 2019; Balcan et al. 2019; Khodak et al. 2019), to the best of our knowledge, Theorems 11 and 12 are the first to establish *both* the AER and IER guarantees for (20) under the federated learning setup.

Curious readers may wonder if the suboptimality of the theoretical guarantees for FedProx (with non-zero λ) is a characteristic of this algorithm or if it is due to the artifact of our technical proof. To answer this question, we conduct a simulation where we apply FedProx with different λs on datasets generated by federated logistic regression (see Appendix D for details). The accuracies versus different values of R is shown in the right panel of Figure 1. As expected by our theory, the performance of FedProx with λ=0 mimics that of PureLocalTraining, whereas the performance with λ=4 resembles that of FedAvg. Interestingly, FedProx with λ=0.44 bears a similar performance with the FedAvg followed by fine tuning strategy, which we know is minimax optimal. This observation supports the conjecture that optimally tuned FedProx is indeed minimax optimal, and the suboptimality of bounds from Theorems 11 and 12 are likely to be a consequence of the artifact of our theoretical analysis.

## 4. Discussion

This paper studies the statistical properties of personalized federated learning. Focusing on strongly-convex, smooth, and bounded empirical risk minimization problems, we have uncovered an intriguing phenomenon that given a specific level of heterogeneity, exactly one of FedAvg or PureLocalTraining is minimax optimal. In the course of proving this result, we obtained a novel analysis of FedProx and introduced a new notion of algorithmic stability termed federated stability, which is possibly of independent interest for analyzing generalization properties in the context of federated learning.

We close this paper by mentioning several open problems.

- *Dependence on problem-specific parameters*. This paper focuses on the dependence on the sample sizes, and in our bounds, the dependence on problem-specific parameters (e.g., the smoothness and strong convexity constants) may not be optimal. This can be problematic if those parameters are not of constant order, and it will be interesting to give a refined analysis that gives optimal dependence on those parameters.
- *A refined analysis of* FedProx. The upper bounds we develop for FedProx, as we have mentioned, do not match our minimax lower bounds. According to a simulated example, we suspect that this is an artifact of our analysis and a refined analysis of FedProx would be a welcome advance.
- *Estimation of the level of heterogeneity and development of adaptive algorithms*. For unsupervised problems where evaluation of a model is difficult, implementation of the oracle dichotomous strategy described in Section 3.3 would require estimating the level of heterogeneity R. Even for supervised problems, estimation of R would be interesting, as it allows one to decide which algorithm to choose without model training. More generally, developing adaptive algorithms that attains the lower bound without prior information of R is an important open problem.
- *Beyond the current heterogeneity assumption*. As discussed in Section 3.3, our theoretical results may not hold globally when one moves from Assumption B to more general heterogeneity assumptions. Establishing the minimax rates and designing provably optimal algorithms under those assumptions are of both theoretical and practical interest.
- *Beyond convexity*. Our analysis is heavily contingent upon the strong convexity of the loss function, which, to the best of our knowledge, is not easily generalizable to the non-convex case. Meanwhile, our notion of heterogeneity, which is based on the distance of optimal local models to the convex combination of them, may not be natural for non-convex problems. It is of interest, albeit difficult, to have a theoretical investigation of personalized federated learning for non-convex problems.

## Footnotes

1. In fact, Algorithm 2 is not exactly the same as the original FedProx algorithm introduced in Li et al. (2018). But since both algorithms share the idea of imposing regularization, we still call Algorithm 2 FedProx for conceptual simplicity.

2. The theories developed by Hanzely et al. (2020) can be useful for such an analysis.

## A. Proof of Theorem 8: Lower Bounds

We start by presenting a lower bound when all $w_{⋆}^{\left(i\right)}$′s are the same.

**Lemma 13 (Lower bound under homogeneity)** *Consider the logistic regression model with* $w_{⋆}^{\left(i\right)}=w_{p}^{\left(\text{global}\right)}$ *for any* i∈[m]. *Then*

$$\underset{{\hat{w}}^{\text{(global) }}}{\text{inf}}\underset{w_{p}^{\left(\text{global }\right)}}{\text{sup}}E_{S}{∥{\hat{w}}^{\left(\text{global }\right)}-w_{p}^{\left(\text{global }\right)}∥}^{2}≳\frac{d}{N}.$$

**Proof** This is a classical result. See, e.g., Example 8.4 of Duchi (2019). ■

**Proof** [Proof of (14)] We first give a lower bound based on the observation that the homogeneous case is in fact included in the parameter space ${𝒫}_{1}$. More explicitly, let us define ${𝒫}_{0}=\left\{\left\{w_{⋆}^{\left(i\right)}\right\}\in {𝒫}_{1}:w_{⋆}^{\left(i\right)}=w_{p}^{\text{(global)}}\forall i\in [m]\right\}$. By Lemma 13, we have

(31)
$$\underset{\left\{{\hat{w}}^{\left(i\right)}\right\}}{\text{inf}}\underset{\left\{w_{⋆}^{\left(i\right)}\right\}\in {𝒫}_{1}}{\text{sup}}\sum_{i\in [m]}p_{i}E_{S}{∥{\hat{w}}^{\left(i\right)}-w_{⋆}^{\left(i\right)}∥}^{2}\geq \underset{\left\{{\hat{w}}^{\left(i\right)}\right\}}{\text{inf}}\underset{\left\{w_{⋆}^{\left(i\right)}\right\}\in {𝒫}_{0}}{\text{sup}}\sum_{i\in \left[m\right]}p_{i}E_{S}{∥{\hat{w}}^{\left(i\right)}-w_{⋆}^{\left(i\right)}∥}^{2}\;=\underset{{\hat{w}}^{\left(\text{global}\right)}}{\text{inf}}\underset{w_{p}^{\left(\text{global}\right)}}{\text{sup}}E_{S}{∥{\hat{w}}^{\left(\text{global}\right)}-w_{p}^{\left(\text{global}\right)}∥}^{2}\;≳\frac{d}{N}.$$

We now use a variant of Assouad’s method (Assouad, 1983) that allows us to tackle multiple datasets. Consider the following data generating process: nature generates $V=\left\{v^{\left(i\right)}:i\in [m]\right\}$ i.i.d. from the uniform distribution on $𝒱=\{\pm 1{\}}^{d}$ and sets $w_{⋆}^{\left(i\right)}=\delta_{i}v^{\left(i\right)}$ for some $\delta_{i}$ such that the following constraint is satisfied:

(32)
$$\sum_{i\in [m]}p_{i}{∥w_{⋆}^{\left(i\right)}-w_{p}^{\left(\text{global}\right)}∥}^{2}=\sum_{i\in [m]}p_{i}{∥\delta_{i}v^{\left(i\right)}-\sum_{s\in [m]}p_{s}\delta_{s}v^{\left(s\right)}∥}^{2}\leq R^{2}.$$

We will specify the choice of $\delta_{i}$’s later. Denoting $E_{X}$ as the marginal expectation operator with respect to all the features $\left\{x_{j}^{\left(i\right)}\right\}$ and $E_{Y|X}$ as the conditional expectation operator with respect to $\left\{y_{j}^{\left(i\right)}\right\}|\left\{x_{j}^{\left(i\right)}\right\}$, we can lower bound the minimax risk by the Bayes risk as follows:

$$\underset{\left\{{\hat{w}}^{\left(i\right)}\right\}}{\text{inf}}\underset{\left\{w_{⋆}^{\left(i\right)}\right\}\in 𝒫}{\text{sup}}\sum_{i\in [m]}p_{i}E_{S}{\left\|{\hat{w}}^{\left(i\right)}-w_{⋆}^{\left(i\right)}\right\|}^{2}\;\geq \underset{\left\{{\hat{w}}^{\left(i\right)}\right\}}{\text{inf}}E_{\left\{v^{\left(i\right)}\right\}}\sum_{i\in [m]}p_{i}E_{S}{\left\|{\hat{w}}^{\left(i\right)}-\delta_{i}v^{\left(i\right)}\right\|}^{2}\;=\underset{\left\{{\hat{v}}^{\left(i\right)}\right\}\subseteq 𝒱}{\text{inf}}\sum_{i\in [m]}p_{i}E_{V,S}{\left\|\delta_{i}{\hat{v}}^{\left(i\right)}-\delta_{i}v^{\left(i\right)}\right\|}^{2}\;\geq E_{X}\sum_{i\in [m]}p_{i}\delta_{i}^{2}\underset{{\hat{v}}^{\left(i\right)}\in 𝒱}{\text{inf}}E_{V,Y|X}{\left\|{\hat{v}}^{\left(i\right)}-v^{\left(i\right)}\right\|}^{2}\;\geq E_{X}\sum_{i\in [m]}p_{i}\delta_{i}^{2}\sum_{k\in [d]}\underset{{\hat{v}}_{k}^{\left(i\right)}\in \{\pm 1\}}{\text{inf}}E_{V,Y|X}{\left({\hat{v}}_{k}^{\left(i\right)}-v_{k}^{\left(i\right)}\right)}^{2}\;≳E_{X}\sum_{i\in [m]}p_{i}\delta_{i}^{2}\sum_{k\in [d]}\underset{{\hat{v}}_{k}^{\left(i\right)}\in \{\pm 1\}}{\text{inf}}{\mathbb{P}}_{V,Y|X}\left({\hat{v}}_{k}^{\left(i\right)}\neq v_{k}^{\left(i\right)}\right)\;=\frac{1}{2}E_{X}\sum_{i\in [m]}p_{i}\delta_{i}^{2}\sum_{k\in [d]}\underset{{\hat{v}}_{k}^{\left(i\right)}\in \{\pm 1\}}{\text{inf}}\left({\mathbb{P}}_{i,+k}\left({\hat{v}}_{k}^{\left(i\right)}=-1\right)+{\mathbb{P}}_{i,-k}\left({\hat{v}}_{k}^{\left(i\right)}=+1\right)\right)$$

where in the last line, we have let $P_{i,\pm k}(\cdot )=P_{V,Y|X}\left(\cdot |v_{k}^{\left(i\right)}=\pm 1\right)$ to denote the probability measure with respect to the randomness in (V,S) conditional on the features $\left\{x_{j}^{\left(i\right)}\right\}$ as well as the realization of $v_{k}^{\left(i\right)}=\pm 1$. More explicitly, we can write

$$P_{i,\pm k}=\left(\underset{s\neq i}{⨂}P_{v^{\left(s\right)}}⊗P_{{\left\{y^{\left(s\right)}\right\}}_{j=1}^{n_{s}}|v^{\left(s\right)},{\left\{x_{j}^{\left(s\right)}\right\}}_{j=1}^{n_{s}}}\right)⊗\left(P_{v^{\left(i\right)}|v_{k}^{\left(i\right)}=\pm 1}⊗P_{{\left\{y_{j}^{\left(i\right)}\right\}}_{j=1}^{n_{i}}|v^{\left(i\right)},v_{k}^{\left(i\right)}=\pm 1,{\left\{x_{j}^{\left(i\right)}\right\}}_{j=1}^{n_{i}}}\right)\;=\frac{1}{2^{(m-1)d+d-1}}\sum_{V\setminus \left\{v_{k}^{\left(i\right)}\right\}}P_{V,i,\pm k},$$

where the ⊗ symbol stands for taking the product of two measures and $P_{V,i,\pm k}$ corresponds to the law of all the labels Y conditional on a specific realization of $\left\{V:v_{k}^{\left(i\right)}=\pm 1\right\}$ and the features X. With the current notations and letting $\|P-Q{\|}_{\text{TV}}$ be the total variation distance between two probability measures P and Q, we can invoke Neyman-Pearson lemma to get

(33)
$$\underset{\left\{{\hat{w}}^{\left(i\right)}\right\}}{\text{inf}}\underset{\left\{w_{⋆}^{\left(i\right)}\right\}\in 𝒫}{\text{sup}}\sum_{i\in [m]}p_{i}E_{S}{∥{\hat{w}}^{\left(i\right)}-w_{⋆}^{\left(i\right)}∥}^{2}\;≳E_{X}\sum_{i\in [m]}p_{i}\delta_{i}^{2}\sum_{k\in [d]}\left(1-{∥P_{i,+k}-P_{i,-k}∥}_{\text{TV}}\right)\;=d\sum_{i\in [m]}p_{i}\delta_{i}^{2}-E_{X}\sum_{i\in [m]}p_{i}\delta_{i}^{2}\sum_{k\in [d]}{∥P_{i,+k}-P_{i,-k}∥}_{\text{TV}}.$$

We then proceed by

$$\sum_{i\in [m]}p_{i}\delta_{i}^{2}\sum_{k\in [d]}{∥P_{i,+k}-P_{i,-k}∥}_{\text{TV}}\;\leq \sum_{i\in [m]}p_{i}\delta_{i}^{2}\sqrt{d}{\left(\sum_{k\in [d]}{∥P_{i,+k}-P_{i,-k}∥}_{\text{TV}}^{2}\right)}^{1/2}\;=\sum_{i\in [m]}p_{i}\delta_{i}^{2}\sqrt{d}{\left(\sum_{k\in [d]}{∥\frac{1}{2^{(m-1)d+d-1}}\sum_{V\setminus \left\{v_{k}^{\left(i\right)}\right\}}P_{V,i,+k}-P_{V,i,-k}∥}_{\text{TV}}^{2}\right)}^{1/2}\;=\sum_{i\in [m]}p_{i}\delta_{i}^{2}\sqrt{d}{\left(\sum_{k\in [d]}\frac{1}{2^{(m-1)d+d-1}}\sum_{V\setminus \left\{v_{k}^{\left(i\right)}\right\}}{∥P_{V,i,+k}-P_{V,i,-k}∥}_{\text{TV}}^{2}\right)}^{1/2},$$

where the last inequality is by convexity of the total variation distance. Note that $P_{V,i,\pm k}$ is the product of biased Rademacher random variables: if we let Rad(p) be the ±1-valued random variable with positive probability p, we can write

$$P_{V,i,\pm k}=\underset{s\in [m]}{⨂}\underset{j\in \left[n_{s}\right]}{⨂}\text{Rad}\left(\frac{1}{1+\text{exp}\left\{-\delta_{s}\left\langle v^{\left(s\right)},x_{j}^{\left(s\right)}\right\rangle \right\}}\right),\;v_{k}^{\left(i\right)}=\pm 1.$$

Thus, by Pinsker’s inequality, we have

$${∥P_{V,i,+k}-P_{V,i,-k}∥}_{\text{TV}}^{2}\;\leq \frac{1}{2}D_{\text{JS}}\left(P_{V,i,+k}\|P_{V,i,-k}\right)\;=\frac{1}{2}\sum_{s\neq i}\sum_{j\in \left[n_{s}\right]}0+\frac{1}{2}\sum_{j\in \left[n_{i}\right]}D_{\text{JS}}\left[\text{Rad}\left(\frac{1}{1+\text{exp}\left\{-\delta_{i}\left\langle v^{\left(i\right)},x_{j}^{\left(i\right)}\right\}\right.}\right)\|\text{Rad}\left(\frac{1}{1+\text{exp}\left\{-\delta_{i}\left\langle {\tilde{v}}^{\left(i\right)},x_{j}^{\left(i\right)}\right\}\right.}\right)\right],$$

where $D_{\text{JS}}(P\|Q)=\frac{D_{\text{KL}}(P\|Q)+D_{\text{KL}}(Q\|P)}{2}$ is the Jensen-Shannon divergence between P and Q, and $v^{\left(s\right)},{\tilde{v}}^{\left(s\right)}$ are two 𝒱-valued vectors that only differs in the k-th coordinate. By a standard calculation, one finds that

$$D_{\text{JS}}\left[\text{Rad}\left(\frac{1}{1+\text{exp}\left\{-\delta_{i}\left\langle v^{\left(i\right)},x_{j}^{\left(i\right)}\right\}\right.}\right)\|\text{Rad}\left(\frac{1}{1+\text{exp}\left\{-\delta_{i}\left\langle {\tilde{v}}^{\left(i\right)},x_{j}^{\left(i\right)}\right\}\right.}\right)\right]\leq \delta_{i}^{2}{\left(v_{k}^{\left(i\right)}-{\tilde{v}}_{k}^{\left(i\right)}\right)}^{2}{\left(x_{j,k}^{\left(i\right)}\right)}^{2}\;=4\delta_{i}^{2}{\left(x_{j,k}^{\left(i\right)}\right)}^{2}.$$

This gives

$${∥P_{V,i,+k}-P_{V,i,-k}∥}_{\text{TV}}^{2}\leq 2\delta_{i}^{2}\sum_{j\in \left[n_{i}\right]}{\left(x_{j,k}^{\left(i\right)}\right)}^{2}\leq 2\delta_{i}^{2}c_{X}^{2}n_{i}.$$

and hence

$$\sum_{i\in [m]}p_{i}\delta_{i}^{2}\sum_{k\in [d]}{∥P_{i,+k}-P_{i,-k}∥}_{\text{TV}}\leq \sqrt{2}c_{X}\sum_{i\in [m]}p_{i}\delta_{i}^{3}dn_{i}^{1/2}.$$

Plugging the above display to (33) gives

(34)
$$\underset{\left\{{\hat{w}}^{\left(i\right)}\right\}}{\text{inf}}\underset{\left\{w_{⋆}^{\left(i\right)}\right\}\in 𝒫}{\text{sup}}\sum_{i\in [m]}p_{i}E_{S}{∥{\hat{w}}^{\left(i\right)}-w_{⋆}^{\left(i\right)}∥}^{2}≳d\left(\sum_{i\in [m]}p_{i}\delta_{i}^{2}-\sqrt{2}c_{X}\sum_{i\in [m]}p_{i}\delta_{i}^{3}\sqrt{n_{i}}\right).$$

To this end, all that is left is to choose $\delta_{i}$ approriately so that (1) the above display is as tight as possible; (2) (32) is satisfied. We consider the following two cases:

1. Assume $R^{2}\geq d\sum_{i\in [m]}p_{i}/n_{i}=dm/N$. Note that we can re-write the requirement (32) to be
  $$d\sum_{i\in [m]}p_{i}\delta_{i}^{2}-{∥\sum_{i\in \left[m\right]}p_{i}\delta_{i}v^{\left(i\right)}∥}^{2}\leq R^{2}.$$
  Under the current assumption, this requirement will be satisfied if we choose $\delta_{i}=\;c/\sqrt{n_{i}}$ for any c≤1. Under such a choice, the right-hand side of (34) becomes $\frac{c^{2}dm}{N}\left(c-\sqrt{2}c_{X}\right)$. Thus, by setting $c=2\sqrt{2}c_{X}$, we get the following lower bound:
  $$\underset{\left\{{\hat{w}}^{\left(i\right)}\right\}}{\text{inf}}\underset{\left\{w_{⋆}^{\left(i\right)}\right\}\in 𝒫}{\text{sup}}\sum_{i\in [m]}p_{i}E_{S}{∥{\hat{w}}^{\left(i\right)}-w_{⋆}^{\left(i\right)}∥}^{2}≳\frac{d}{N/m}.$$
2. Assume $R^{2}\leq d\sum_{i\in [m]}p_{i}/n_{i}=dm/N$. Note that if we set $\delta_{i}\equiv \delta =cR/\sqrt{d}$ where c≤1, (32) reads
  $$c^{2}R^{2}-{∥\sum_{i\in \left[m\right]}p_{i}\delta_{i}v^{\left(i\right)}∥}^{2}\leq R^{2},$$
  which trivially holds. Now, the right-hand side of (34) becomes
  $$c^{2}R^{2}\left(1-\sqrt{2}cc_{X}\sum_{i\in [m]}p_{i}R\sqrt{n_{i}}/\sqrt{d}\right).$$
  Since $p_{i}=n_{i}/N$ and $n_{i}≍N/m$, our assumption on R gives
  $$\sqrt{2}cc_{X}\sum_{i\in \left[m\right]}p_{i}R\sqrt{n_{i}}/\sqrt{d}≲\sum_{i}\frac{n_{i}}{N}\cdot \sqrt{\frac{mn_{i}}{N}}=1.$$
  This means that we can choose c to be a small constant such that the following lower bound holds:
  $$\underset{\left\{{\hat{w}}^{\left(i\right)}\right\}}{\text{inf}}\underset{\left\{w_{⋆}^{\left(i\right)}\right\}\in 𝒫}{\text{sup}}\sum_{i\in [m]}p_{i}E_{S}{∥{\hat{w}}^{\left(i\right)}-w_{⋆}^{\left(i\right)}∥}^{2}≳R^{2}.$$

Summarizing the above two cases, we arrive at

$$\underset{\left\{{\hat{w}}^{\left(i\right)}\right\}}{\text{inf}}\underset{\left\{w_{⋆}^{\left(i\right)}\right\}\in 𝒫}{\text{sup}}\sum_{i\in [m]}p_{i}E_{S}{∥{\hat{w}}^{\left(i\right)}-w_{⋆}^{\left(i\right)}∥}^{2}≳\frac{d}{N/m}\wedge R^{2}.$$

Combining the above bound with (31), we get

$$\underset{\left\{{\hat{w}}^{\left(i\right)}\right\}}{\text{inf}}\underset{\left\{w_{⋆}^{\left(i\right)}\right\}\in 𝒫}{\text{sup}}\sum_{i\in [m]}p_{i}E_{S}{∥{\hat{w}}^{\left(i\right)}-w_{⋆}^{\left(i\right)}∥}^{2}≳\frac{d}{N/m}\wedge R^{2}+\frac{d}{N},$$

which is the desired result. ■

**Proof** [Proof of (15)] The proof is similar to the proof of (16), and we only provide a sketch here. Without loss of generality we consider the first client. By the same arguments as in the proof of (16), the left-hand side of (15) is lower bounded by a constant multiple of d/N. Now, by considering the same prior distribution on 𝒫 as in the proof of (16), we get

$$\underset{{\overset{-}{w}}^{\left(1\right)}}{\text{inf}}\underset{\left\{w^{\left(i\right)}\right\}\in 𝒫}{\text{sup}}E_{S}{∥{\overset{-}{w}}^{\left(1\right)}-w_{⋆}^{\left(1\right)}∥}^{2}≳d\delta_{1}^{2}\left(1-\delta_{1}\sqrt{n_{1}}\right),$$

where the $\delta_{i}$ ‘s should obey the following inequality:

$${∥\delta_{i}v^{\left(i\right)}-\sum_{s\in [m]}p_{s}\delta_{s}v^{\left(s\right)}∥}^{2}\leq R^{2}.$$

Choosing $\delta_{i}≍1/\sqrt{n_{i}}$ when R≥dm/N and $\delta_{i}≍R/\sqrt{d}$ otherwise, we arrive at

$$\underset{{\overset{-}{w}}^{\left(1\right)}}{\text{inf}}\underset{\left\{w^{\left(i\right)}\right\}\in 𝒫}{\text{sup}}E_{S}{∥{\overset{-}{w}}^{\left(1\right)}-w_{⋆}^{\left(1\right)}∥}^{2}≳\frac{d}{n_{1}}\wedge R^{2},$$

and the proof is concluded. ■

## B. Optimization Convergence of FedProx

This section concerns the optimization convergence of FedProx. We first introduce some notations. Let $w_{t,k}^{\left(i\right)}$ be the output of k-th step of Algorithm 2 when the initial local model is given by $w_{t}^{\left(i\right)}\equiv w_{t,0}^{\left(i\right)}\equiv w_{t-1,K}^{\left(i\right)}$, let $I_{t,k}^{\left(t\right)}$ be the corresponding minibatch taken, and denote the initial global model by $w_{t}^{\text{(global)}}$. Let ${ℱ}_{t,k}$ be the sigma algebra generated by the randomness by Algorithm 2 up to $w_{t,k}^{\left(i\right)}$, namely the randomness in $\left\{{𝒞}_{\tau },\left\{I_{\tau ,l}^{\left(i\right)}:i\in {𝒞}_{\tau },0\leq {\left.\left.l\leq K_{\tau }-1\right\}\right\}}_{\tau =0}^{t-1},{𝒞}_{t}\right.\right.$, and $\left\{I_{t,l}^{\left(i\right)}:i\in {𝒞}_{t},0\leq l\leq k-1\right\}$. For notational convenience we let ${𝒞}_{T}=[m]$ (i.e., all clients are involved in local training in Stage II of Algorithm 2). Then the sequence $\left\{w_{t,k}^{\left(i\right)}\right\}$ is adapted to the following filtration:

$${ℱ}_{0,0}\subseteq {ℱ}_{0,1}\subseteq \cdots \subseteq {ℱ}_{0,K}\subseteq {ℱ}_{1,0}\subseteq {ℱ}_{1,1}\subseteq \cdots \subseteq {ℱ}_{1,K}\subseteq \cdots \subseteq {ℱ}_{T,K}.$$

We write the optimization problem (20) as

(35)
$$\underset{w^{\left(\text{global}\right)}\in 𝒲}{\text{min}}\sum_{i\in [m]}p_{i}F_{i}\left(w^{\left(\text{global}\right)},S_{i}\right),$$

where

(36)
$$F_{i}\left(w^{\left(\text{global}\right)},S_{i}\right)=\underset{w^{\left(i\right)}\in 𝒲}{\text{min}}\left\{L_{i}\left(w^{\left(i\right)},S_{i}\right)+\frac{\lambda }{2}{∥w^{\left(\text{global}\right)}-w^{\left(i\right)}∥}^{2}\right\}.$$

To simplify notations, we introduce the proximal opertor

(37)
$${\text{Prox}}_{L_{i}/\lambda }\left(w^{\left(\text{global}\right)}\right)={\text{Prox}}_{L_{i}/\lambda }\left(w^{\left(\text{global}\right)},S_{i}\right)={\text{argmin }}_{w^{\left(i\right)}\in 𝒲}\left\{L_{i}\left(w^{\left(i\right)},S_{i}\right)+\frac{\lambda }{2}{∥w^{\left(\text{global}\right)}-w^{\left(i\right)}∥}^{2}\right\}.$$

The high-level idea of this proof is to regard $\lambda \sum_{i\in {𝒞}_{t}}\left(w_{t}^{\left(\text{global}\right)}-w_{t+1}^{\left(i\right)}\right)/B^{\left(\text{global}\right)}$ as a biased stochastic gradient of $\frac{1}{n}\sum_{i\in [m]}F_{i}\left(w_{t}^{\left(\text{global}\right)},S_{i}\right)$. This idea has appeared in various places (see, e.g., the proof of Proposition 5 in Denevi et al. (2019) and the proof of Theorem 1 in Dinh et al. (2020)). However, the implementation of this idea in our case is more complicated than the above mentioned works in that (1) we are not in an online learning setup (compared to Denevi et al. (2019)); (2) we don’t need to assume all clients are training at every round (compared to Dinh et al. (2020)); and (3) we use local SGD for the inner loop (instead of assuming the inner loop can be solved with arbitrary precision as assumed in Dinh et al. (2020)), so the gradient norm depends on λ, and could in principle be arbitrarily large, which causes extra complications.

**Lemma 14 (Convergence of the inner loop)** *Let* Assumption A(a, b) *holds. Choose* $\eta_{t,k}^{\left(i\right)}=\frac{1}{\left(\mu +\lambda )(k+1\right)}$. *Then for any* k≥0, *we have*

$$E\left[{∥w_{t,k}^{\left(i\right)}-{\text{Prox}}_{L_{i}/\lambda }\left(w_{t}^{\left(\text{global }\right)}\right)∥}^{2}|{ℱ}_{t,0},i\in {𝒞}_{t}\right]\leq \frac{8\beta^{2}D^{2}}{\mu^{2}\left(k+1\right)}.$$

**Proof** See Appendix B.1.■

**Lemma 15 (Convergence of the outer loop)** *Let the assumptions in* Lemma 14 *hold. Choose* $\eta_{t}^{\text{(global)}}=\frac{2(\mu +\lambda )}{\lambda \mu (t+1)}$ *and assume*

(38)
$$K_{\tau }+1\geq \frac{\left(4\tau +20\right)\lambda^{2}\beta^{2}D^{2}}{\mu^{2}\left(\beta^{2}D^{2}\wedge 2\lambda \|\ell {\|}_{\infty }\wedge \lambda^{2}D^{2}\right)}\;\forall 0\leq \tau \leq t-1.$$

*Then for any* t≥0*, we have*

(39)
$$E_{{𝒜}_{\text{FP}}}{∥w_{t}^{\left(\text{global}\right)}-{\tilde{w}}^{\left(\text{global}\right)}∥}^{2}\leq \frac{12(\lambda +\mu {)}^{2}m\|p{\|}^{2}\left(\beta^{2}D^{2}\wedge 2\lambda \|\ell {\|}_{\infty }\wedge \lambda^{2}D^{2}\right)}{\lambda^{2}\mu^{2}\left(t+1\right)},$$

*where the expectation is taken over the randomness in* Algorithm 2.

**Proof** See Appendix B.2.■

**Proposition 16 (Optimization error of** ${𝒜}_{FP}$) *Under the assumptions of* Lemma 14 *and* 15, *for any dataset* $S\sim {⨂}_{i}{𝒟}_{i}^{⊗n_{i}}$*, we have*

$$E_{{𝒜}_{\text{FP}}}\left[{ℰ}_{\text{OPT}}\right]\leq \frac{4(\beta +\lambda )\beta^{2}D^{2}}{\mu^{2}\left(K_{T}+1\right)}+\frac{6(\lambda +\mu {)}^{2}m\|p{\|}^{2}\left(\beta^{2}D^{2}\wedge 2\lambda \|\ell {\|}_{\infty }\wedge \lambda^{2}D^{2}\right)}{\lambda \mu^{2}(t+1)}$$

**Proof** By definition we have

$$E_{{𝒜}_{\text{FP}}}\left[{ℰ}_{\text{OPT}}\right]≔E_{{𝒜}_{\text{FP}}}\left[\sum_{i\in [m]}p_{i}\left(L_{i}\left(w_{T+1}^{\left(i\right)},S_{i}\right)+\frac{\lambda }{2}{∥w_{T}^{\left(\text{global }\right)}-w_{T+1}^{\left(i\right)}∥}^{2}\right)-\sum_{i\in [m]}p_{i}F_{i}\left({\tilde{w}}^{\left(\text{global }\right)},S_{i}\right)\right]\;\overset{(\text{ a) }}{\leq }\sum_{i\in [m]}\frac{p_{i}(\beta +\lambda )}{2}\cdot E_{{𝒜}_{\text{FP}}}{∥w_{T+1}^{\left(i\right)}-{\text{Prox}}_{L_{i}/\lambda }\left(w_{T}^{\left(\text{global }\right)}\right)∥}^{2}+E_{{𝒜}_{\text{FP}}}\left[\sum_{i\in [m]}p_{i}F_{i}\left(w_{T}^{\text{(global) }},S_{i}\right)-\sum_{i\in [m]}p_{i}F_{i}\left({\tilde{w}}^{\text{(global) }},S_{i}\right)\right]\;\overset{(\text{ b) }}{\leq }\frac{4(\beta +\lambda )\beta^{2}D^{2}}{\mu^{2}\left(K_{T}+1\right)}+\frac{\lambda }{2}E_{{𝒜}_{\text{FP}}}{∥w_{T}^{\text{(global) }}-{\tilde{w}}^{\text{(global) }}∥}^{2}\;\overset{\text{ (c) }}{\leq }\frac{4(\beta +\lambda )\beta^{2}D^{2}}{\mu^{2}\left(K_{T}+1\right)}+\frac{6(\lambda +\mu {)}^{2}m\|p{\|}^{2}\left(\beta^{2}D^{2}\wedge 2\lambda \|\ell {\|}_{\infty }\wedge \lambda^{2}D^{2}\right)}{\lambda \mu^{2}(t+1)}.$$

where (a) is by smoothness of $L_{i}$, (b) is by Lemma 14 and λ-smoothness of $\sum_{i\in [m]}p_{i}F_{i}$ (which holds by Lemma 17), and (c) is by Lemma 15.■

### B.1 Proof of Lemma 14: Convergence of the Inner Loop

The proof is an adaptation of the proof of Lemma 1 in Rakhlin et al. (2011). However, we need to deal with the extra complication that the hyperparameter λ can in principle be arbitrarily large. We start by noting that

$${∥w_{t,k+1}^{\left(i\right)}-{\text{Prox}}_{L_{i}/\lambda }\left(w_{t}^{\left(\text{global}\right)}\right)∥}^{2}\;\;={∥{𝒫}_{𝒲}\left[w_{t,k}^{\left(i\right)}-\frac{\eta_{t,k}^{\left(i\right)}}{B^{\left(i\right)}}\sum_{j\in I_{t,k}^{\left(i\right)}}\left(\nabla \ell \left(w_{t,k}^{\left(i\right)},z_{j}^{\left(i\right)}\right)+\lambda \left(w_{t,k}^{\left(i\right)}-w_{t}^{\left(\text{global}\right)}\right)\right)\right]-{\text{Prox}}_{L_{i}/\lambda }\left(w_{t}^{\left(\text{global}\right)}\right)∥}^{2}\;\leq {∥w_{t,k}^{\left(i\right)}-\frac{\eta_{t,k}^{\left(i\right)}}{B^{\left(i\right)}}\sum_{j\in I_{t,k}^{\left(i\right)}}\left(\nabla \ell \left(w_{t,k}^{\left(i\right)},z_{j}^{\left(i\right)}\right)+\lambda \left(w_{t,k}^{\left(i\right)}-w_{t}^{\left(\text{global}\right)}\right)\right)-{\text{Prox}}_{L_{i}/\lambda }\left(w_{t}^{\left(\text{global}\right)}\right)∥}^{2}\;\left.=\|w_{t,k}^{\left(i\right)}-{\text{Prox}}_{L_{i}/\lambda }\left(w_{t}^{\left(\text{global}\right)}\right)\right)∥{\;}^{2}+∥\frac{\eta_{t,k}^{\left(i\right)}}{B^{\left(i\right)}}\sum_{j\in I_{t,k}^{\left(i\right)}}\left(\nabla \ell \left(w_{t,k}^{\left(i\right)},z_{j}^{\left(i\right)}\right)+\lambda \left(w_{t,k}^{\left(i\right)}-w_{t}^{\left(\text{global}\right)}\right)\right){\|}^{2}-2\left\langle w_{t,k}^{\left(i\right)}-{\text{Prox}}_{L_{i}/\lambda }\left(w_{t}^{\left(\text{global}\right)}\right),\frac{\eta_{t,k}^{\left(i\right)}}{B^{\left(i\right)}}\sum_{j\in I_{t,k}^{\left(i\right)}}\left(\nabla \ell \left(w_{t,k}^{\left(i\right)},z_{j}^{\left(i\right)}\right)+\lambda \left(w_{t,k}^{\left(i\right)}-w_{t}^{\left(\text{global}\right)}\right)\right)\right\rangle ,$$

where the inequality is because ${\text{Prox}}_{L_{i}/\lambda }\left(w_{t}^{\left(\text{global}\right)}\right)\in 𝒲$ and ${𝒫}_{𝒲}$ is non-expansive. Now by strong convexity and unbiasedness of the stochastic gradients, we have

$$E\left[\langle w_{t,k}^{\left(i\right)}-Prox_{L_{i}/\lambda }\left(w_{t}^{\left(\text{global }\right)}\right),\frac{1}{B^{\left(i\right)}}\sum_{j\in I_{t,k}^{\left(i\right)}}\left(\nabla \ell \left(w_{t,k}^{\left(i\right)},z_{j}^{\left(i\right)}\right)+\lambda \left(w_{t,k}^{\left(i\right)}-w_{t}^{\left(\text{global }\right)}\right)\right)\rangle |\;{ℱ}_{t,k},i\in {𝒞}_{t}\right]\;\geq \left(L_{i}\left(w_{t,k}^{\left(i\right)},S_{i}\right)+\frac{\lambda }{2}\|w_{t,k}^{\left(i\right)}-w^{\left(\text{global }\right)}{\|}^{2}\right)-\left(L_{i}\left(Prox_{L_{i}/\lambda }\left(w_{t}^{\left(\text{global }\right)}\right),S_{i}\right)+\frac{\lambda }{2}\|Prox_{L_{i}/\lambda }\left(w_{t}^{\left(\text{global }\right)}\right)-w_{t}^{\left(\text{global }\right)}{\|}^{2}\right)+\frac{1}{2}\left(\mu_{i}+\frac{\lambda n}{mn_{i}}\right)\|w_{t,k}^{\left(i\right)}-Prox_{L_{i}/\lambda }\left(w_{t}^{\left(\text{global }\right)}\right){\|}^{2}\;\geq (\mu +\lambda )\|w_{t,k}^{\left(i\right)}-Prox_{L_{i}/\lambda }\left(w_{t}^{\text{(global) }}\right){\|}^{2}.$$

On the other hand, applying Lemma 19 gives

$$E\left[\left.{∥\frac{\eta_{t,k}^{\left(i\right)}}{B^{\left(i\right)}}\sum_{j\in I_{t,k}^{\left(i\right)}}\left(\nabla \ell \left(w_{t,k}^{\left(i\right)},z_{j}^{\left(i\right)}\right)+\lambda \left(w_{t,k}^{\left(i\right)}-w_{t}^{\left(\text{global }\right)}\right)\right)∥}^{2}\right|\;{ℱ}_{t,k},i\in {𝒞}_{t}\right]\;={\left(\eta_{t,k}^{\left(i\right)}\right)}^{2}\cdot \left[\frac{n_{i}/B^{\left(i\right)}-1}{n_{i}\left(n_{i}-1\right)}\sum_{j\in \left[n_{i}\right]}{∥\nabla \ell \left(w_{t,k}^{\left(i\right)},z_{j}^{\left(i\right)}\right)-\overset{-}{\nabla \ell \left(w_{t,k}^{\left(i\right)},z_{∙}^{\left(i\right)}\right)}∥}^{2}\right.\left.+{∥\frac{1}{n_{i}}\sum_{j\in \left[n_{i}\right]}\nabla \ell \left(w_{t,k}^{\left(i\right)},z_{j}^{\left(i\right)}\right)+\lambda \left(w_{t,k}^{\left(i\right)}-w_{t}^{\left(\text{global }\right)}\right)∥}^{2}\right]\;\leq 2{\left(\eta_{t,k}^{\left(i\right)}\right)}^{2}\beta^{2}D^{2}\cdot \frac{n_{i}/B^{\left(i\right)}-1}{\left(n_{i}-1\right)}+{\left(\beta +\frac{\lambda n}{mn_{i}}\right)}^{2}{∥w_{t,k}^{\left(i\right)}-{\text{Prox}}_{L_{i}/\lambda }\left(w_{t}^{\left(\text{global }\right)}\right)∥}^{2},$$

where in the second line we let $\overset{-}{\nabla \ell \left(w_{t,k}^{\left(i\right)},z_{.}^{\left(i\right)}\right)}≔\sum_{j\in \left[n_{i}\right]}\nabla \ell \left(w_{t,k}^{\left(i\right)},z_{j}^{\left(i\right)}\right)/n_{i}$, and in the last line is by the β-smoothness of ℓ(⋅,z). Thus, we get

(40)
Ewt,k+1(i)-ProxLi/λwt(global)2|ℱt,k,i∈𝒞t ≤1-2ηt,ki(μ+λ)+ηt,ki2(β+λ)2wt,ki-ProxLi/λwtglobal2 +2ηt,ki2β2D2⋅niB(i)-1ni-1.

We then proceed by induction. Note that if $k+1\leq \frac{8\beta^{2}}{\mu^{2}}$, then we have the following trivial bound:

(41)
$$E\left[{∥w_{t,k}^{\left(i\right)}-{\text{Prox}}_{L_{i}/\lambda }\left(w_{t}^{\left(\text{global}\right)}\right)∥}^{2}|{ℱ}_{t,0},i\in {𝒞}_{t}\right]\leq D^{2}\leq \frac{8\beta^{2}D^{2}}{\mu^{2}\left(k+1\right)},$$

where the first inequality is by $w_{t,k}^{\left(i\right)},{\text{Prox}}_{L_{i}/\lambda }\left(w_{t}^{\left(\text{global}\right)}\right)\in 𝒲$ and the second inequality is by our assumption on k. Thus, it suffices to show

(42)
$$E\left[{∥w_{t,k+1}^{\left(i\right)}-{\text{Prox}}_{L_{i}/\lambda }\left(w_{t}^{\left(\text{global}\right)}\right)∥}^{2}|{ℱ}_{t,0},i\in {𝒞}_{t}\right]\leq \frac{8\beta^{2}D^{2}}{\mu^{2}(k+2)}$$

based on the inductive hypothesis (41) and $k+1\geq 8\beta^{2}/\mu^{2}$. By the recursive relationship (40) and taking expectation, we have

$$E\left[{∥w_{t,k+1}^{\left(i\right)}-{\text{Prox}}_{L_{i}/\lambda }\left(w_{t}^{\left(\text{global }\right)}\right)∥}^{2}|{ℱ}_{t,0},i\in {𝒞}_{t}\right]\;\leq \left[1-2\eta_{t,k}^{\left(i\right)}(\mu +\lambda )+{\left(\eta_{t,k}^{\left(i\right)}\right)}^{2}(\beta +\lambda {)}^{2}\right]\frac{8\beta^{2}D^{2}}{k+1}+2{\left(\eta_{t,k}^{\left(i\right)}\right)}^{2}\beta^{2}D^{2}\cdot \frac{n_{i}/B^{\left(i\right)}-1}{\left(n_{i}-1\right)}.$$

Hence (42) is satisfied if

$$8\beta^{2}D^{2}\cdot \left[\frac{1}{k+2}-\frac{1}{k+1}+\frac{2\eta_{t,k}^{\left(i\right)}}{k+1}(\mu +\lambda )-\frac{{\left(\eta_{t,k}^{\left(i\right)}\right)}^{2}}{k+1}(\beta +\lambda {)}^{2}\right]\geq 2{\left(\eta_{t,k}^{\left(i\right)}\right)}^{2}\beta^{2}D^{2}\cdot \frac{n_{i}/B^{\left(i\right)}-1}{\left(n_{i}-1\right)}.$$

By our choice of $\eta_{t,k}^{\left(i\right)}$, the above display is equivalent to

$$8\beta^{2}D^{2}\cdot \left[-\frac{1}{\left(k+1)(k+2\right)}+\frac{2}{(k+1{)}^{2}}-\frac{1}{(k+1{)}^{3}}{\left(\frac{\beta +\lambda }{\mu +\lambda }\right)}^{2}\right]\geq \frac{2\beta^{2}D^{2}}{(\mu +\lambda {)}^{2}(k+1{)}^{2}}\cdot \frac{n_{i}/B^{\left(i\right)}-1}{n_{i}-1}$$

which is further equivalent to

$$8\beta^{2}D^{2}\cdot \left[-\frac{k+1}{k+2}+2-\frac{1}{k+1}{\left(\frac{\beta +\lambda }{\mu +\lambda }\right)}^{2}\right]\geq \frac{2\beta^{2}D^{2}}{(\mu +\lambda {)}^{2}}\cdot \frac{n_{i}/B^{\left(i\right)}-1}{n_{i}-1}.$$

We now claim that

$$\frac{1}{k+1}{\left(\frac{\beta +\lambda }{\mu +\lambda }\right)}^{2}\leq \frac{1}{2}.$$

Indeed, since $k+1\geq 8\beta^{2}/\mu^{2}$, (1) if λ≤β, then the left-hand side above is less than $\frac{4\beta^{2}}{\mu^{2}(k+1)}\leq \frac{1}{2}$; and (2) if λ≥β, the left-hand side above is less than $\frac{4}{k+1}\leq \frac{\mu^{2}}{2\beta^{2}}\leq \frac{1}{2}$. By the above claim, (42) would hold if

$$4\beta^{2}D^{2}\geq \frac{2\beta^{2}D^{2}}{(\mu +\lambda {)}^{2}}\cdot \frac{n_{i}/B^{\left(i\right)}-1}{n_{i}-1}.$$

We finish the proof by noting that the right-hand side above is bounded above by $\frac{2\beta^{2}D^{2}}{\mu^{2}}$.

### B.2 Proof of Lemma 15: Convergence of the Outer Loop

By construction we have

$${∥w_{t}^{\left(\text{global }\right)}-{\tilde{w}}^{\text{(global) }}∥}^{2}={∥\frac{\lambda m\eta_{t}^{\left(\text{global }\right)}}{B^{\left(\text{global }\right)}}\sum_{i\in {𝒞}_{t}}p_{i}\left(w_{t}^{\left(\text{global }\right)}-w_{t+1}^{\left(i\right)}\right)∥}^{2}\;={∥w_{t}^{\left(\text{global }\right)}-{\tilde{w}}^{\left(\text{global }\right)}∥}^{2}+{∥\frac{\lambda m\eta_{t}^{\left(\text{global }\right)}}{B^{\left(\text{global }\right)}}\sum_{i\in {𝒞}_{t}}p_{i}\left(w_{t}^{\left(\text{global }\right)}-w_{t+1}^{\left(i\right)}\right)∥}^{2}-2\left\langle w_{t}^{\left(\text{global }\right)}-{\tilde{w}}^{\left(\text{global }\right)},\frac{\lambda m\eta_{t}^{\left(\text{global }\right)}}{B^{\left(\text{global }\right)}}\sum_{i\in {𝒞}_{t}}p_{i}\left(w_{t}^{\left(\text{global }\right)}-w_{t+1}^{\left(i\right)}\right)\right\rangle \;\leq {∥w_{t}^{\left(\text{global }\right)}-{\tilde{w}}^{\text{(global) }}∥}^{2}-\underset{\text{I}}{\underbrace{2\left\langle w_{t}^{\left(\text{global }\right)}-{\tilde{w}}^{\text{(global) }},\frac{\lambda m\eta_{t}^{\text{(global) }}}{B^{\left(\text{global }\right)}}\sum_{i\in {𝒞}_{t}}p_{i}\left(w_{t}^{\left(\text{global }\right)}-{\text{Prox}}_{L_{i}/\lambda }\left(w_{t}^{\left(\text{global }\right)}\right)\right)\right\rangle }}+\underset{\text{II }}{\underbrace{2{∥\frac{\lambda m\eta_{t}^{\left(\text{global }\right)}}{B^{\text{(global) }}}\sum_{i\in {𝒞}_{t}}p_{i}\left(w_{t}^{\text{(global) }}-{\text{Prox}}_{L_{i}/\lambda }\left(w_{t}^{\text{(global) }}\right)\right)∥}^{2}}}+\underset{\text{III }}{\underbrace{2{∥\frac{\lambda m\eta_{t}^{\left(\text{global }\right)}}{B^{\text{(global) }}}\sum_{i\in {𝒞}_{t}}p_{i}\left({\text{Prox}}_{L_{i}/\lambda }\left(w_{t}^{\left(\text{global }\right)}\right)-w_{t+1}^{\left(i\right)}\right)∥}^{2}}}-\underset{\text{IV }}{\underbrace{2\left\langle w_{t}^{\left(\text{global }\right)}-{\tilde{w}}^{\left(\text{global }\right)},\frac{\lambda m\eta_{t}^{\left(\text{global }\right)}}{B^{\text{(global) }}}\sum_{i\in {𝒞}_{t}}p_{i}\left({\text{Prox}}_{L_{i}/\lambda }\left(w_{t}^{\left(\text{global }\right)}\right)\right)-w_{t+1}^{\text{(i) }}\right\rangle }}.$$

We first consider Term I. Note that $\frac{\lambda m}{B^{\text{(global)}}}\sum_{i\in {𝒞}_{t}}p_{i}\left(w_{t}^{\text{(global)}}-{\text{Prox}}_{L_{i}/\lambda }\left(w_{t}^{\text{(global)}}\right)\right)$ is an unbiased stochastic gradient of $\sum_{i}p_{i}F_{i}$, which is $\mu_{F}=\lambda \mu /(\lambda +\mu )$-strongly convex. Thus, we have

$$E\left[\text{I}|{ℱ}_{t-1,K_{t-1}}\right]\;=2\eta_{t}^{\left(\text{global }\right)}\left\langle w_{t}^{\left(\text{global }\right)}-{\tilde{w}}^{\left(\text{global }\right)},\sum_{i\in [m]}p_{i}\nabla F_{i}\left(w_{t}^{\left(\text{global }\right)},S_{i}\right)\right\rangle \;\geq 2\eta_{t}^{\left(\text{global }\right)}\mu_{F}{∥w_{t}^{\left(\text{global }\right)}-{\tilde{w}}^{\left(\text{global }\right)}∥}^{2}.$$

Now for Term II, we have

$$E\left[\text{ II }|{ℱ}_{t-1,K_{t-1}}\right]\;\leq 2{\left(\eta_{t}^{\text{(global) }}\right)}^{2}\cdot E\left[\left.{\left(\frac{1}{B^{\left(\text{global }\right)}}\sum_{i\in {𝒞}_{t}}mp_{i}\right)}^{2}\right|\;{ℱ}_{t-1,K_{t-1}}\right]\cdot \underset{i\in [m]}{\text{max}}{∥\nabla F_{i}\left(w_{t}^{\left(\text{global }\right)},S_{i}\right)∥}^{2}\;\leq 2{\left(\eta_{t}^{\left(\text{global }\right)}\right)}^{2}\cdot E\left[\left.{\left(\frac{1}{B^{\left(\text{global }\right)}}\sum_{i\in {𝒞}_{t}}mp_{i}\right)}^{2}\right|\;{ℱ}_{t-1,K_{t-1}}\right]\cdot \underset{i\in [m]}{\text{max}}\cdot \left(\beta^{2}D^{2}\wedge 2\lambda \|\ell {\|}_{\infty }\wedge \lambda^{2}D^{2}\right)\;\leq 2{\left(\eta_{t}^{\left(\text{global }\right)}\right)}^{2}\cdot \left(\frac{1}{m}\sum_{i\in [m]}{\left(mp_{i}-1\right)}^{2}+1\right)\cdot \left(\beta^{2}D^{2}\wedge 2\lambda \|\ell {\|}_{\infty }\wedge \lambda^{2}D^{2}\right)\;=2{\left(\eta_{t}^{\left(\text{global }\right)}\right)}^{2}m\|p{\|}^{2}\left(\beta^{2}D^{2}\wedge 2\lambda \|\ell {\|}_{\infty }\wedge \lambda^{2}D^{2}\right),$$

where the second line is by Lemma 18 and the third line is by Lemma 19. For Term III, we invoke Lemma 14 to get

$$E\left[\text{ III }|{ℱ}_{t-1,K_{t-1}}\right]\;\leq 2\lambda^{2}{\left(\eta_{t}^{\left(\text{global }\right)}\right)}^{2}\cdot \frac{8\beta^{2}D^{2}}{\mu^{2}\left(K_{t}+1\right)}\cdot E\left[\left.{\left(\frac{1}{B^{\text{(global) }}}\sum_{i\in {𝒞}_{t}}mp_{i}\right)}^{2}\right|\;{ℱ}_{t-1,K_{t-1}}\right]\;\leq \frac{16\lambda^{2}{\left(\eta_{t}^{\left(\text{global }\right)}\right)}^{2}\beta^{2}D^{2}m\|p{\|}^{2}}{\mu^{2}\left(K_{t}+1\right)},$$

where the last line is again by Lemma 19. For Term IV, we invoke Young’s inequality for products to get

$$E\left[-\text{IV}|{ℱ}_{t-1,K_{t-1}}\right]\;\leq \left(\eta_{t}^{\left(\text{global}\right)}\mu_{F}\right){∥w_{t}^{\left(\text{global}\right)}-{\tilde{w}}^{\left(\text{global}\right)}∥}^{2}+{\left(\eta_{t}^{\left(\text{global}\right)}\mu_{F}\right)}^{-1}\cdot E\left[\left.\frac{\text{III}}{2}\right|\;{ℱ}_{t-1,K_{t-1}}\right]\;\leq \left(\eta_{t}^{\left(\text{global}\right)}\mu_{F}\right){∥w_{t}^{\left(\text{global}\right)}-{\tilde{w}}^{\left(\text{global}\right)}∥}^{2}+{\left(\eta_{t}^{\left(\text{global}\right)}\mu_{F}\right)}^{-1}\cdot \frac{8\lambda^{2}{\left(\eta_{t}^{\left(\text{global}\right)}\right)}^{2}\beta^{2}D^{2}m\|p{\|}^{2}}{\mu^{2}\left(K_{t}+1\right)}.$$

Summarizing the above bounds on the four terms, we arrive at

$$E\left[{∥w_{t+1}^{\left(\text{global}\right)}-{\tilde{w}}^{\text{(global) }}∥}^{2}|{ℱ}_{t-1,K_{t-1}}\right]\;\leq \left(1-\eta_{t}^{\left(\text{global}\right)}\mu_{F}\right){∥w_{t}^{\left(\text{global}\right)}-{\tilde{w}}^{\left(\text{global}\right)}∥}^{2}+2\underset{\text{V}}{\underbrace{{\left(\eta_{t}^{\left(\text{global}\right)}\right)}^{2}m\|p{\|}^{2}\left(\beta^{2}D^{2}\wedge 2\lambda \|\ell {\|}_{\infty }\wedge \lambda^{2}D^{2}\right)}}\;+\underset{\text{VI}}{\underbrace{\frac{\lambda^{2}{\left(\eta_{t}^{\left(\text{global}\right)}\right)}^{2}\beta^{2}D^{2}m\|p{\|}^{2}}{\mu^{2}\left(K_{t}+1\right)}\cdot \left(16+\frac{8}{\delta_{t}^{\left(\text{global}\right)}\mu_{F}}\right)}}.$$

We claim that VI≤V. Indeed, with our choice of $\eta_{t}^{\left(\text{global}\right)}=\frac{2}{\mu_{F}(t+1)}$, with some algebra, one recognizes that this claim is equivalent to

$$\frac{20+4t}{\mu^{2}\left(K_{t}+1\right)}\leq \left(\frac{1}{\lambda^{2}}\wedge \frac{2\|\ell {\|}_{\infty }}{\lambda \beta^{2}D^{2}}\wedge \frac{1}{\beta^{2}}\right),$$

which is exactly (38). Thus, we have

(43)
$$E\left[{∥w_{t+1}^{\left(\text{global}\right)}-{\tilde{w}}^{\text{(global)}}∥}^{2}|{ℱ}_{t-1,K_{t-1}}\right]\;\leq \left(1-\eta_{t}^{\left(\text{global}\right)}\mu_{F}\right){∥w_{t}^{\left(\text{global}\right)}-{\tilde{w}}^{\text{(global)}}∥}^{2}+3\cdot \text{V}\;=\left(1-\frac{2}{t+1}\right){∥w_{t}^{\left(\text{global}\right)}-{\tilde{w}}^{\left(\text{global}\right)}∥}^{2}+\frac{12m\|p{\|}^{2}\left(\beta^{2}D^{2}\wedge 2\lambda \|\ell {\|}_{\infty }\wedge \lambda^{2}D^{2}\right)}{\mu_{F}^{2}(t+1{)}^{2}}.$$

We then proceed by induction. For the base case, we invoke the strong convexity of $\sum_{i}p_{i}F_{i}$ and Lemma 18 to get

$$\frac{\mu_{F}^{2}}{4}{∥w_{0}^{\left(\text{global }\right)}-{\tilde{w}}^{\text{(global) }}∥}^{2}\leq {∥\sum_{i\in \left[m\right]}p_{i}\nabla F_{i}\left(w_{0}^{\text{(global) }},S_{i}\right)∥}^{2}\leq \beta^{2}D^{2}\wedge 2\lambda \|\ell {\|}_{\infty }\wedge \lambda^{2}D^{2}.$$

Along with the fact that $1={\left(\sum_{i\in [m]}p_{i}\right)}^{2}\leq m\|p{\|}^{2}$, we conclude that (15) is true for t=0. Now assume (39) hold for any 0≤t≤τ. For t=τ+1, using (43) and the inductive hypothesis, we have

$$E_{{𝒜}_{\text{FP}}}{∥w_{\tau +1}^{\left(\text{global }\right)}-{\tilde{w}}^{\text{(global) }}∥}^{2}\leq \left(1-\frac{2}{\tau +1}\right)\frac{12m\|p{\|}^{2}\left(\beta^{2}D^{2}\wedge 2\lambda \|\ell {\|}_{\infty }\wedge \lambda^{2}D^{2}\right)}{(\tau +1)\mu_{F}^{2}}+\frac{12m\|p{\|}^{2}\left(\beta^{2}D^{2}\wedge 2\lambda \|\ell {\|}_{\infty }\wedge \lambda^{2}D^{2}\right)}{(\tau +1{)}^{2}\mu_{F}^{2}}\;=\left(\frac{1}{\tau +1}-\frac{1}{(\tau +1{)}^{2}}\right)\cdot \frac{12m\|p{\|}^{2}\left(\beta^{2}D^{2}\wedge 2\lambda \|\ell {\|}_{\infty }\wedge \lambda^{2}D^{2}\right)}{\mu_{F}^{2}}\;\leq \frac{12m\|p{\|}^{2}\left(\beta^{2}D^{2}\wedge 2\lambda \|\ell {\|}_{\infty }\wedge \lambda^{2}D^{2}\right)}{(\tau +2)\mu_{F}^{2}},$$

which is the desired result.

### B.3 Auxiliary lemmas

**Lemma 17 (Convexity and smoothness** $F_{i}$) Under Assumption A(b), *each* $F_{i}$ *is* λ-*smooth and* $\frac{\mu \lambda }{\mu +\lambda }$-*strongly convex*.

**Proof** The smoothness is a standard fact about the Moreau envelope. The strongly convex constant of $F_{i}$ follows from Theorem 2.2 of Lemaréchal and Sagastizábal (1997). ■

**Lemma 18 (A priori gradient norm bound)** *Under* Assumption A(a, b), *for any* w∈𝒲 *and* i∈[m], *we have*

$${∥\nabla F_{i}\left(w,S_{i}\right)∥}^{2}\leq \beta^{2}D^{2}\wedge 2\lambda \|\ell {\|}_{\infty }\wedge \lambda^{2}D^{2}.$$

**Proof** Since $\nabla F_{i}\left(w,S_{i}\right)=\lambda \left(w-{\text{Prox}}_{L_{i}/\lambda }(w)\right)$, its norm is trivially bounded by λD. Now, since ${\text{Prox}}_{L_{i}/\lambda }(w)$ achieves a lower objective value than w for the objective function $L_{i}\left(\cdot ,S_{i}\right)+\frac{\lambda }{2}\|w-\cdot {\|}^{2}$, we have

$$\frac{\lambda }{2}\|w-{\text{Prox}}_{L_{i}/\lambda }\left(w\right){\|}^{2}\leq L_{i}\left(w,S_{i}\right)-L_{i}\left({\text{Prox}}_{L_{i}/\lambda }\left(w\right),S_{i}\right)\leq \|\ell {\|}_{\infty },$$

and hence ${∥\nabla F_{i}\left(w,S_{i}\right)∥}^{2}\leq 2\lambda \|\ell {\|}_{\infty }$. Finally, by the first-order condition, we have

$$\nabla L_{i}\left({\text{Prox}}_{L_{i}/\lambda }(w),S_{i}\right)+\lambda \left({\text{Prox}}_{L_{i}/\lambda }(w)-w\right)=0.$$

Hence, we get $∥\nabla F_{i}\left(w,S_{i}\right)∥=∥\nabla L_{i}\left({\text{Prox}}_{L_{i}/\lambda }(w),S_{i}\right)∥\leq \beta D$. ■

**Lemma 19 (Variance of minibatch sampling)** *Let* ℬ⊆[n] *be a randomly sampled batch with batch size* B *and let* ${\left\{x_{i}\right\}}_{i=1}^{n}\subseteq R^{d}$ *be an arbitrary set of vectors, then*

$$E_{ℬ}{∥\frac{1}{B}\sum_{i\in ℬ}x_{i}∥}^{2}=\frac{n/B-1}{n(n-1)}\sum_{i\in [n]}{∥x_{i}-\overset{‾}{x}∥}^{2}+\|\overset{‾}{x}{\|}^{2}\leq \frac{1}{n}\sum_{i\in [n]}{∥x_{i}-\overset{‾}{x}∥}^{2}+\|\overset{‾}{x}{\|}^{2},$$

*where* $\overset{‾}{x}≔\sum_{i\in [n]}x_{i}/n$.

**Proof** Since $E_{ℬ}\sum_{i\in ℬ}x_{i}/B=\overset{‾}{x}$, we have

$$E_{ℬ}{∥\frac{1}{B}\sum_{i\in ℬ}x_{i}∥}^{2}=E_{ℬ}{∥\frac{1}{B}\sum_{i\in ℬ}x_{i}-\overset{‾}{x}∥}^{2}+\|\overset{‾}{x}{\|}^{2}\;=\frac{1}{B^{2}}\left(\sum_{i\in [n]}1\{i\in ℬ\}{∥x_{i}-\overset{‾}{x}∥}^{2}+2\sum_{i<j}1\{i,j\in ℬ\}\left\langle x_{i}-\overset{‾}{x},x_{j}-\overset{‾}{x}\right\rangle \right)+\|\overset{‾}{x}{\|}^{2}\;=\frac{1}{B^{2}}\left(\frac{B}{n}\sum_{i\in [n]}{∥x_{i}-\overset{‾}{x}∥}^{2}+\frac{2B(B-1)}{n(n-1)}\sum_{i<j}\left\langle x_{i}-\overset{‾}{x},x_{j}-\overset{‾}{x}\right\rangle \right)+\|\overset{‾}{x}{\|}^{2},$$

where the last line is by $P_{ℬ}(i\in ℬ)=B/n$ and $P_{ℬ}(i,j\in ℬ)=B(B-1)n^{-1}(n-1{)}^{-1}$ for any i≠j. Now, since $\sum_{i\in [n]}{∥x_{i}-\overset{‾}{x}∥}^{2}+2\sum_{i<j}\left\langle x_{i}-\overset{‾}{x},x_{j}-\overset{‾}{x}\right\rangle =0$, we arrive at

$$E_{ℬ}{∥\frac{1}{B}\sum_{i\in ℬ}x_{i}∥}^{2}\;=\frac{1}{B^{2}}\left(\frac{B}{n}-\frac{B\left(B-1\right)}{n\left(n-1\right)}\right)\sum_{i}{∥x_{i}-\overset{‾}{x}∥}^{2}+\|x{\|}^{2}\;=\frac{n/B-1}{n(n-1)}\sum_{i\in [n]}{∥x_{i}-\overset{‾}{x}∥}^{2}+\|\overset{‾}{x}{\|}^{2},$$

which is the desired result. ■

## C. Proofs of Upper Bounds

### C.1 Proof of Theorem 6

In this proof, we let ${\hat{w}}^{\text{(global)}}$ be the global minimizer of (8) and we write $w_{\text{avg}}^{\text{(global,p)}}\equiv w_{\text{p}}^{\text{(global)}}$ when there is no ambiguity.

**Proof** [Proof of (9)] We have

$$0=-\;\sum_{i\in [m]}p_{i}L_{i}\left({\hat{w}}^{\left(\text{global }\right)}(S),S_{i}\right)+\sum_{i\in [m]}p_{i}L_{i}\left({\hat{w}}^{\left(\text{global }\right)}(S),S_{i}\right)\;\leq -\;\sum_{i\in [m]}p_{i}L_{i}\left({\hat{w}}^{\left(\text{global }\right)}(S),S_{i}\right)+\sum_{i\in [m]}p_{i}L_{i}\left(w_{⋆}^{\left(1\right)},S_{i}\right)\;=-\;\sum_{i\in [m]}\frac{p_{i}}{n_{i}}\sum_{j\in \left[n_{i}\right]}\left(\ell \left({\hat{w}}^{\left(\text{global }\right)}\left(S^{\setminus (i,j)}\right),z_{j}^{\left(i\right)}\right)-\ell \left(w_{⋆}^{\left(1\right)},z_{j}^{\left(i\right)}\right)\right)+\sum_{i\in [m]}\frac{p_{i}}{n_{i}}\sum_{j\in \left[n_{i}\right]}\left(\ell \left({\hat{w}}^{\left(\text{global }\right)}\left(S^{\setminus (i,j)}\right),z_{j}^{\left(i\right)}\right)-\ell \left({\hat{w}}^{\left(\text{global }\right)}(S),z_{j}^{\left(i\right)}\right)\right),$$

where $S^{\setminus (i,j)}$ stands for the dataset formed by replacing $z_{j}^{\left(i\right)}$ by another $z_{i,j}^{\prime }\sim {𝒟}_{i}$, which is independent of everything else. Taking expectation in both sides, we get

$$0\leq -\sum_{i\in [m]}p_{i}\cdot E_{S,Z_{i}\sim {𝒟}_{i}}\left[\ell \left({\hat{w}}^{\left(\text{global }\right)}(S),Z_{i}\right)-\ell \left(w_{⋆}^{\left(1\right)},Z_{i}\right)\right]+\sum_{i\in [m]}\frac{p_{i}}{n_{i}}\sum_{j\in \left[n_{i}\right]}E_{S,z_{i,j}^{\prime }}\left[\ell \left({\hat{w}}^{\left(\text{global }\right)}\left(S^{\setminus (i,j)}\right),z_{j}^{\left(i\right)}\right)-\ell \left({\hat{w}}^{\left(\text{global }\right)}(S),z_{j}^{\left(i\right)}\right)\right]\;=-\;\sum_{i\in [m]}p_{i}\cdot E_{S,Z_{i}\sim {𝒟}_{i}}\left[\ell \left({\hat{w}}^{\left(\text{global }\right)}(S),Z_{i}\right)-\ell \left(w_{⋆}^{\left(i\right)},Z_{i}\right)\right]-\sum_{i\in [m]}p_{i}\cdot E_{Z_{i}\sim {𝒟}_{i}}\left[\ell \left(w_{⋆}^{\left(i\right)},Z_{i}\right)-\ell \left(w_{⋆}^{\left(1\right)},Z_{i}\right)\right]+\sum_{i\in [m]}\frac{p_{i}}{n_{i}}\sum_{j\in \left[n_{i}\right]}E_{S,z_{i,j}^{\prime }}\left[\ell \left({\hat{w}}^{\left(\text{global }\right)}\left(S^{\setminus (i,j)}\right),z_{j}^{\left(i\right)}\right)-\ell \left({\hat{w}}^{\left(\text{global }\right)}(S),z_{j}^{\left(i\right)}\right)\right].$$

Noting that $w_{⋆}^{\left(i\right)}$ is the argmin of $E_{Z_{i}\sim {𝒟}_{i}}\left[\ell \left(\cdot ,Z_{i}\right)\right]$ and invoking the β-smoothness assumption, we get

$$E_{S}\left[{\text{AER}}_{p}\left({\hat{w}}^{\left(\text{global }\right)}\right)\right]\;\leq \beta \sum_{i\in [m]}p_{i}{∥w_{⋆}^{\left(1\right)}-w_{⋆}^{\left(i\right)}∥}^{2}+\sum_{i\in [m]}\frac{p_{i}}{n_{i}}\sum_{j\in \left[n_{i}\right]}E_{S,z_{i,j}^{\prime }}\left[\ell \left({\hat{w}}^{\left(\text{global }\right)}\left(S^{\setminus (i,j)}\right),z_{j}^{\left(i\right)}\right)-\ell \left({\hat{w}}^{\left(\text{global }\right)}(S),z_{j}^{\left(i\right)}\right)\right]\;\leq 2\beta {∥w_{⋆}^{\left(1\right)}-w_{p}^{\left(\text{global }\right)}∥}^{2}+2\beta \sum_{i\in [m]}p_{i}∥w_{⋆}^{\left(i\right)}-w_{p}^{\left(\text{global }\right)}∥+\sum_{i\in [m]}\frac{p_{i}}{n_{i}}\sum_{j\in \left[n_{i}\right]}E_{S,z_{i,j}^{\prime }}\left[\ell \left({\hat{w}}^{\left(\text{global }\right)}\left(S^{\setminus (i,j)}\right),z_{j}^{\left(i\right)}\right)-\ell \left({\hat{w}}^{\left(\text{global }\right)}(S),z_{j}^{\left(i\right)}\right)\right].$$

Taking a weighted average, we arrive at

(44)
$$E_{S}\left[{\text{AER}}_{p}\left({\hat{w}}^{\left(\text{global}\right)}\right)\right]\;\leq 4\beta R^{2}+\sum_{i\in [m]}\frac{p_{i}}{n_{i}}\sum_{j\in \left[n_{i}\right]}E_{S,z_{i,j}^{\prime }}\left[\ell \left({\hat{w}}^{\left(\text{global }\right)}\left(S^{\setminus (i,j)}\right),z_{j}^{\left(i\right)}\right)-\ell \left({\hat{w}}^{\left(\text{global }\right)}(S),z_{j}^{\left(i\right)}\right)\right]$$

To bound the second term in the right-hand side above, we bound the federated stability of ${\hat{w}}^{\text{(global)}}$. Without loss of generality we consider the first client. By μ-strongly convexity of $L_{1}$, for any $j_{1}\in \left[n_{1}\right]$ we have

(45)
$$\frac{\mu }{2}{∥{\hat{w}}^{\text{(global) }}(S)-{\hat{w}}^{\text{(global) }}\left(S^{\setminus \left(1,j_{1}\right)}\right)∥}^{2}\;\leq \sum_{i\in [m]}p_{i}\left(L_{i}\left({\hat{w}}^{\text{(global) }}\left(S^{\setminus \left(1,j_{1}\right)}\right),S_{i}\right)-L_{i}\left({\hat{w}}^{\text{(global) }}(S),S_{i}\right)\right)\;=\left(\sum_{i\neq 1}p_{i}L_{i}\left({\hat{w}}^{\left(\text{global }\right)}\left(S^{\setminus \left(1,j_{1}\right)}\right),S_{i}\right)+p_{1}L_{1}\left({\hat{w}}^{\text{(global) }}\left(S^{\setminus \left(1,j_{1}\right)}\right),S_{1}^{\setminus j_{1}}\right)\right)-\left(\sum_{i\neq 1}p_{i}L_{i}\left({\hat{w}}^{\text{(global) }}(S),S_{i}\right)+p_{1}L_{1}\left({\hat{w}}^{\text{(global) }}(S),S_{1}^{\setminus j_{1}}\right)\right)+p_{1}\left(L_{1}\left({\hat{w}}^{\left(\text{global }\right)}\left(S^{\setminus \left(1,j_{1}\right)}\right),S_{1}\right)-L_{1}\left({\hat{w}}^{\text{(global) }}\left(S^{\setminus \left(1,j_{1}\right)}\right),S_{1}^{\setminus j_{1}}\right)\right)+p_{1}\left(L_{1}\left({\hat{w}}^{\text{(global) }}(S),S_{1}^{\setminus j_{1}}\right)-L_{1}\left({\hat{w}}^{\text{(global) }}(S),S_{1}\right)\right)\;\leq p_{1}\left(L_{1}\left({\hat{w}}^{\text{(global)}}\left(S^{\setminus \left(1,j_{1}\right)}\right),S_{1}\right)-L_{1}\left({\hat{w}}^{\text{(global)}}\left(S^{\setminus \left(1,j_{1}\right)}\right),S_{1}^{\setminus j_{1}}\right)\right)+p_{1}\left(L_{1}\left({\hat{w}}^{\text{(global)}}(S),S_{1}^{\setminus j_{1}}\right)-L_{1}\left({\hat{w}}^{\text{(global)}}(S),S_{1}\right)\right)\;=\frac{p_{1}}{n_{1}}\left(\ell \left({\hat{w}}^{\text{(global)}}\left(S^{\setminus \left(1,j_{1}\right)}\right),z_{j_{1}}^{\left(1\right)}\right)-\ell \left({\hat{w}}^{\text{(global)}}(S),z_{j_{1}}^{\left(1\right)}\right)\right)+\frac{p_{1}}{n_{1}}\left(\ell \left({\hat{w}}^{\text{(global)}}(S),z_{1,j_{1}}^{\prime }\right)-\ell \left({\hat{w}}^{\text{(global)}}\left(S^{\setminus \left(1,j_{1}\right)}\right),z_{1,j_{1}}^{\prime }\right)\right),$$

where the second inequality is because ${\hat{w}}^{\left(\text{global}\right)}\left(S^{\setminus \left(1,j_{1}\right)}\right)$ minimizes $L_{1}\left(\cdot ,S_{1}^{\setminus j_{1}}\right)+\sum_{i\neq 1}n_{i}L_{i}\left(\cdot ,S_{i}\right)$. By an identical argument as in the proof of Lemma 26, we have

(46)
$$\ell \left({\hat{w}}^{\text{(global)}}\left(S^{\setminus \left(1,j_{1}\right)}\right),z_{j_{1}}^{\left(1\right)}\right)-\ell \left({\hat{w}}^{\text{(global)}}(S),z_{j_{1}}^{\left(1\right)}\right)\;\leq \sqrt{2\beta \|\ell {\|}_{\infty }}\cdot ∥{\hat{w}}^{\text{(global)}}(S)-{\hat{w}}^{\text{(global)}}\left(S^{\setminus \left(1,j_{1}\right)}\right)∥+\frac{\beta }{2}{∥{\hat{w}}^{\text{(global)}}(S)-{\hat{w}}^{\text{(global)}}\left(S^{\setminus \left(1,j_{1}\right)}\right)∥}^{2}$$

The same bound also holds for $\ell \left({\hat{w}}^{\text{(global)}}(S),z_{1,j_{1}}^{\prime }\right)-\ell \left({\hat{w}}^{\left(\text{global}\right)}\left(S^{\setminus \left(1,j_{1}\right)}\right),z_{1,j_{1}}^{\prime }\right)$. Plugging these two bounds to (45) and rearranging terms, we get

$$\left(\frac{\mu }{2}-\frac{\beta p_{1}}{n_{1}}\right)∥{\hat{w}}^{\left(\text{global }\right)}(S)-{\hat{w}}^{\left(\text{global }\right)}\left(S^{\setminus \left(1,j_{1}\right)}\right)∥\leq \frac{2\sqrt{2\beta \|\ell {\|}_{\infty }}\cdot p_{1}}{n_{1}}.$$

Since $n_{1}\geq 4\beta p_{1}/\mu$, we in fact have

$$\frac{\mu }{4}∥{\hat{w}}^{\text{(global) }}(S)-{\hat{w}}^{\text{(global) }}\left(S^{\setminus \left(1,j_{1}\right)}\right)∥\leq \frac{2\sqrt{2\beta \|\ell {\|}_{\infty }}\cdot p_{1}}{n_{1}}.$$

Plugging the above display back to (46), we arrive at

$$\ell \left({\hat{w}}^{\text{(global) }}\left(S^{\setminus \left(1,j_{1}\right)}\right),z_{j_{1}}^{\left(1\right)}\right)-\ell \left({\hat{w}}^{\text{(global) }}\left(S\right),z_{j_{1}}^{\left(1\right)}\right)\leq \frac{16\beta \|\ell {\|}_{\infty }p_{1}}{\mu n_{1}}\left(1+\frac{4\beta p_{1}}{\mu n_{1}}\right)\leq \frac{32\beta \|\ell {\|}_{\infty }p_{1}}{\mu n_{1}},$$

where the last inequality is again by $n_{1}\geq 4\beta p_{1}/\mu$. The desired result follows by plugging the above inequality back to (44). ■

**Proof** [Proof of (10)] Without loss of generality we consider the first client. Since $w_{⋆}^{\left(1\right)}$ is the minimizer of $E_{Z_{1}\sim {𝒟}_{1}}\ell \left(\cdot ,Z_{1}\right)$, by β-smoothness we have

(47)
$$E_{Z_{1}\sim {𝒟}_{1}}\left[\ell \left({\hat{w}}^{\left(\text{global}\right)},Z_{1}\right)-\ell \left(w_{⋆}^{\left(1\right)},Z_{1}\right)\right]\;≲\beta \cdot E_{Z_{1}\sim {𝒟}_{1}}{∥{\hat{w}}^{\left(\text{global}\right)}-w_{⋆}^{\left(1\right)}∥}^{2}\;≲\beta \cdot E_{Z_{1}\sim {𝒟}_{1}}{∥{\hat{w}}^{\left(\text{global}\right)}-w_{p}^{\left(\text{global}\right)}∥}^{2}+\beta R^{2},$$

where the last inequality is by Part (b) of Assumption B. By optimality of ${\hat{w}}^{\text{(global)}}$ and the strong convexity of $L_{i}\prime$s, we have

$$\left\langle \sum_{i\in [m]}p_{i}\nabla L_{i}\left(w_{p}^{\left(\text{global }\right)},S_{i}\right),{\hat{w}}^{\left(\text{global }\right)}-w_{p}^{\left(\text{global }\right)}\right\rangle +\frac{\mu }{2}{∥{\hat{w}}^{\left(\text{global }\right)}-w_{p}^{\left(\text{global }\right)}∥}^{2}\leq 0.$$

If ${\hat{w}}^{\text{(global)}}-w_{p}^{\text{(global)}}=0$ then we are done. Otherwise, the above display gives

$$∥{\hat{w}}^{\text{(global) }}-w_{p}^{\left(\text{global }\right)}∥\;\leq \frac{2}{\mu }∥\sum_{i\in [m]}p_{i}\nabla L_{i}\left(w_{p}^{\left(\text{global }\right)},S_{i}\right)∥\;\leq \frac{2}{\mu }\left(∥\sum_{i\in [m]}p_{i}\nabla L_{i}\left(w_{⋆}^{\left(i\right)},S_{i}\right)∥+∥\sum_{i\in [m]}p_{i}\left(\nabla L_{i}\left(w_{p}^{\left(\text{global }\right)},S_{i}\right)-\nabla L_{i}\left(w_{⋆}^{\left(i\right)},S_{i}\right)\right)∥\right)\;\leq \frac{2}{\mu }\left(∥\sum_{i\in [m]}p_{i}\nabla L_{i}\left(w_{⋆}^{\left(i\right)},S_{i}\right)∥+\beta \sum_{i\in [m]}p_{i}∥w_{p}^{\left(\text{global }\right)}-w_{⋆}^{\left(i\right)}∥\right)\;\leq \frac{2}{\mu }\left(∥\sum_{i\in [m]}p_{i}\nabla L_{i}\left(w_{⋆}^{\left(i\right)},S_{i}\right)∥+\beta R\right).$$

Thus, we get

$${∥{\hat{w}}^{\left(\text{global }\right)}-w_{p}^{\left(\text{global }\right)}∥}^{2}\leq \frac{8}{\mu^{2}}\left({∥\sum_{i\in [m]}p_{i}\nabla L_{i}\left(w_{⋆}^{\left(i\right)},S_{i}\right)∥}^{2}+\beta^{2}R^{2}\right).$$

Taking expectation with respect to the sample S at both sides, we have

$$E_{S}{∥{\hat{w}}^{\left(\text{global }\right)}-w_{p}^{\left(\text{global }\right)}∥}^{2}\;≲\frac{1}{\mu^{2}}E_{S}{∥\sum_{i\in \left[m\right]}p_{i}\left(\nabla L_{i}\left(w_{⋆}^{\left(i\right)},S_{i}\right)-E_{S}\left[\nabla L_{i}\left(w_{⋆}^{\left(i\right)},S_{i}\right)\right]\right)∥}^{2}+\frac{\beta^{2}R^{2}}{\mu^{2}}\;\leq \frac{1}{\mu^{2}}\cdot \sum_{i\in \left[m\right]}\frac{p_{i}^{2}\sigma^{2}}{n_{i}}+\frac{\beta^{2}R^{2}}{\mu^{2}}.$$

Plugging the above inequality to (47) gives the desired result. ■

### C.2 Proof of Proposition 10

**Proof** [Proof of (21)] By the definitions of the AER and ${ℰ}_{\text{OPT}}$, we have

$${\text{AER}}_{p}={ℰ}_{\text{OPT}}\;+\frac{\lambda }{2}\sum_{i\in [m]}p_{i}\left({∥{\tilde{w}}^{\left(\text{global }\right)}(S)-{\tilde{w}}^{\left(i\right)}(S)∥}^{2}-{∥{\hat{w}}^{\left(\text{global }\right)}(S)-{\hat{w}}^{\left(i\right)}(S)∥}^{2}\right)\;+\sum_{i\in [m]}p_{i}\left(E_{Z_{i}\sim {𝒟}_{i}}\left[\ell \left({\hat{w}}^{\left(i\right)}(S),Z_{i}\right)\right]-L_{i}\left({\hat{w}}^{\left(i\right)}(S),S_{i}\right)\right)\;+\sum_{i\in [m]}p_{i}\left(L_{i}\left({\tilde{w}}^{\left(i\right)}(S),S_{i}\right)-E_{Z_{i}\sim {𝒟}_{i}}\left[\ell \left(w_{⋆}^{\left(i\right)},Z_{i}\right)\right]\right).$$

By the basic inequality (23), we can bound the AER by

$$\;{\text{AER}}_{\text{p}}\leq {ℰ}_{\text{OPT}}+\frac{\lambda }{2}\sum_{i\in [m]}p_{i}{∥w_{p}^{\left(\text{global }\right)}-w_{⋆}^{\left(i\right)}∥}^{2}+\sum_{i\in [m]}p_{i}\left(E_{Z_{i}\sim {𝒟}_{i}}\left[\ell \left({\hat{w}}^{\left(i\right)}(S),Z_{i}\right)\right]-L_{i}\left({\hat{w}}^{\left(i\right)}(S),S_{i}\right)\right)+\sum_{i\in [m]}p_{i}\left(L_{i}\left(w_{⋆}^{\left(i\right)},S_{i}\right)-E_{Z_{i}\sim {𝒟}_{i}}\left[\ell \left(w_{⋆}^{\left(i\right)},Z_{i}\right)\right]\right).$$

Now, invoking federated stability, we can further bound the AER by

$${\text{AER}}_{p}\leq {ℰ}_{\text{OPT}}\;+\frac{\lambda }{2}\sum_{i\in [m]}p_{i}{∥w_{p}^{\left(\text{global }\right)}-w_{⋆}^{\left(i\right)}∥}^{2}+2\sum_{i\in [m]}p_{i}\gamma_{i}\;+\sum_{i\in [m]}p_{i}\cdot \frac{1}{n_{i}}\sum_{j\in \left[n_{i}\right]}E_{z_{i,j}^{\prime }\sim {𝒟}_{i}}\left[E_{Z_{i}\sim {𝒟}_{i}}\left[\ell \left({\hat{w}}^{\left(i\right)}(S^{\setminus (i,j)}),Z_{i}\right)\right]-\ell \left({\hat{w}}^{\left(i\right)}\left(S^{\setminus (i,j)}\right),z_{j}^{\left(i\right)}\right)\right]\;+\sum_{i\in [m]}p_{i}\left(L_{i}\left(w_{⋆}^{\left(i\right)},S_{i}\right)-E_{Z_{i}\sim {𝒟}_{i}}\left[\ell \left(w_{⋆}^{\left(i\right)},Z_{i}\right)\right]\right),$$

where $S^{\setminus (i,j)}$ is the dataset formed by replacing $z_{j}^{\left(i\right)}$ with a new sample $z_{i,j}^{\prime }$, and here we are choosing $z_{i,j}^{\prime }$ to be an independent sample from ${𝒟}_{i}$. Note that the last two terms of the above display have mean zero under the randomness of the algorithm 𝒜, the dataset S, and $\left\{z_{i,j}^{\prime }:i\in [m],j\in \left[n_{i}\right]\right\}$. Thus, the desired result follows by taking expectation in both sides. ■

**Proof** [Proof of (22)] Without loss of generality we consider the first client. By definitions of ${\text{IER}}_{1}$ and ${ℰ}_{\text{OPT}}$, we have

$$p_{1}\cdot {\text{IER}}_{1}={ℰ}_{\text{OPT}}\;+\sum_{i\in [m]}p_{i}\left(L_{i}\left({\tilde{w}}^{\left(i\right)}(S),S_{i}\right)+\frac{\lambda }{2}{∥{\tilde{w}}^{\left(\text{global }\right)}(S)-{\tilde{w}}^{\left(i\right)}(S)∥}^{2}\right)\;-\sum_{i\in [m]}p_{i}\left(L_{i}\left({\hat{w}}^{\left(i\right)}(S),S_{i}\right)+\frac{\lambda }{2}{∥{\hat{w}}^{\left(\text{global }\right)}(S)-{\hat{w}}^{\left(i\right)}(S)∥}^{2}\right)\;+p_{1}E_{Z_{1}\sim {𝒟}_{1}}\left[\ell \left({\hat{w}}^{\left(1\right)}(S),Z_{1}\right)-\ell \left(w_{⋆}^{\left(1\right)},Z_{1}\right)\right].$$

Invoking the basic inequality (24), with some algebra, we arrive at

$$p_{1}\cdot {\text{IER}}_{1}\;\leq {ℰ}_{\text{OPT}}+\frac{p_{1}\lambda }{2}{∥{\hat{w}}^{\left(\text{global }\right)}(S)-w_{⋆}^{\left(1\right)}∥}^{2}+p_{1}\left(E_{Z_{1}\sim {𝒟}_{1}}\left[\ell \left({\hat{w}}^{\left(1\right)}(S),Z_{1}\right)\right]-L_{1}\left({\hat{w}}^{\left(1\right)}(S),S_{1}\right)\right)\;+p_{1}\left(L_{1}\left(w_{⋆}^{\left(1\right)},S_{1}\right)-E_{Z_{1}\sim {𝒟}_{1}}\left[\ell \left(w_{⋆}^{\left(1\right)},Z_{1}\right)\right]\right).$$

Now, invoking federated stability for the first client, we can bound its IER by

$$p_{1}\cdot {\text{IER}}_{1}\leq {ℰ}_{\text{OPT}}\;+\frac{p_{1}\lambda }{2}{∥{\hat{w}}^{\left(\text{global }\right)}\left(S\right)-w_{⋆}^{\left(1\right)}∥}^{2}+2p_{1}\gamma_{1}\;+\frac{p_{1}}{n_{1}}\sum_{j\in \left[n_{1}\right]}E_{z_{1,j}^{\prime }\sim {𝒟}_{1}}\left[E_{Z_{1}\sim {𝒟}_{1}}\left[\ell \left({\hat{w}}^{\left(1\right)}\left(S^{\setminus \left(1,j\right)}\right),Z_{1}\right)\right]-\ell \left({\hat{w}}^{\left(1\right)}\left(S^{\setminus \left(1,j\right)}\right),z_{j}^{\left(1\right)}\right)\right]+p_{1}\left(L_{1}\left(w_{⋆}^{\left(1\right)},S_{1}\right)-E_{Z_{1}\sim {𝒟}_{1}}\left[\ell \left(w_{⋆}^{\left(1\right)},Z_{1}\right)\right]\right),$$

where we recall that $S^{\setminus (1,j)}$ is the dataset formed by replacing $z_{j}^{\left(1\right)}$ with a new sample $z_{1,j}^{\prime }$, and here we are choosing $z_{1,j}^{\prime }$ to be an independent sample from ${𝒟}_{1}$. We finish the proof by taking the expectation with respect to $𝒜,S,\left\{z_{1,j}^{\prime }:j\in \left[n_{1}\right]\right\}$ at both sides. ■

### C.3 Proof of Theorem 11

In this proof, we let $𝒜=\left({\hat{w}}^{\text{(global)}},\left\{{\hat{w}}^{\left(i\right)}\right\}\right.$ be a generic algorithm that tries to minimize (20). For notiontional simplicity, we use $a_{n}{≲}_{\beta }b_{n}$ (resp. $a_{n}{≳}_{\beta }b_{n}$) to denote that $a_{n}\leq C_{\beta }b_{n}$ (resp. $a_{n}\geq C_{\beta }b_{n}$) for large n, where $C_{\beta }$ has explicit dependence on a parameter β.

Recall that $\left({\tilde{w}}^{\text{(global)}},\left\{{\tilde{w}}^{\left(i\right)}\right\}\right)$ is the global minimizer of (20), and recall the notations in (35)–(37). We start by bounding the federated stability of approximate minimizers of (20). We need the following definition.

**Definition 20 (Approximate minimizers)** *We say an algorithm* $𝒜=\left({\hat{w}}^{\left(\text{global}\right)},{\left\{{\hat{w}}^{\left(i\right)}\right\}}_{1}^{m}\right)$ *produces an* $\left(\varepsilon^{\left(\text{global}\right)},{\left\{\varepsilon^{\left(i\right)}\right\}}_{1}^{m}\right)$-*minimizer of the objective function* (20) *on the dataset* S *if the following two conditions hold*:

1. *there exist a positive constant* $\varepsilon^{\text{(global)}}$ *such that* $∥{\hat{w}}^{\text{(global)}}-{\tilde{w}}^{\text{(global)}}∥\leq \varepsilon^{\text{(global)}}$;
2. *for any* i∈[m], *there exist a positive constant* $\varepsilon^{\left(i\right)}$ *such that* $∥{\hat{w}}^{\left(i\right)}-{\text{Prox}}_{L_{i}/\lambda }\left({\hat{w}}^{\left(\text{global}\right)}\right)∥\leq \varepsilon^{\left(i\right)}$.

The stability bound is as follows.

**Proposition 21 (Federated stability of approximate minimizers)** *Let* Assumption A(b) *holds, and consider an algorithm* $𝒜=\left({\hat{w}}^{\left(\text{global}\right)},{\left\{{\hat{w}}^{\left(i\right)}\right\}}_{1}^{m}\right)$ *that produces an* $\left(\varepsilon^{\left(\text{global}\right)},{\left\{\varepsilon^{\left(i\right)}\right\}}_{1}^{m}\right)$-*minimizer of the objective function* (20) *on the dataset* S. *Assume in addition that*

(48)
$$n_{i}\geq \frac{4\beta }{\mu },\;p_{i}\lambda \leq \frac{\mu }{16}\;\forall i\in \left[m\right].$$

*Then* 𝒜 *has federated stability*

$$\gamma_{i}\leq \frac{160\beta \|\ell {\|}_{\infty }}{n_{i}\left(\mu +\lambda \right)}+{\text{Err}}_{i},$$

*Where*

$${\text{Err}}_{i}≔2\sqrt{2\beta \|\ell {\|}_{\infty }}\left[4\varepsilon^{\text{(global) }}\left(\frac{\beta +\lambda }{\mu +\lambda }+\frac{3\lambda }{\mu }\right)+\varepsilon^{\left(i\right)}\left(\sqrt{\frac{\beta +\lambda }{\mu +\lambda }}+\frac{16p_{i}\lambda }{\mu }\right)\right]\;+8\beta^{2}\left[16{\left(\varepsilon^{\text{(global) }}\right)}^{2}{\left(\frac{\beta +\lambda }{\mu +\lambda }+\frac{3\lambda }{\mu }\right)}^{2}+{\left(\varepsilon^{\left(i\right)}\right)}^{2}{\left(\sqrt{\frac{\beta +\lambda }{\mu +\lambda }}+\frac{16p_{i}\lambda }{\mu }\right)}^{2}\right]$$

*is the error term due to not exactly minimizing the soft weight sharing objective* (20).

**Proof** See Appendix C.3.1. ■

Taking the optimization error into account, we have the following result.

**Proposition 22 (Federated stability of** ${𝒜}_{FP}$) *Let* Assumption A(a, b) *and* (48) *hold. Run* ${𝒜}_{\text{FP}}$ *with hyperparameters chosen as in* Lemma 14 *and* 15. *Then, as long as*

(49)
$$T\geq C_{1}\cdot \lambda^{2}(\lambda \vee 1{)}^{2}m\|p{\|}^{2}n_{i}^{2},\;K_{T}\geq C_{2}\cdot \lambda^{2}(\lambda \vee 1{)}^{2}p_{i}^{2}n_{i}^{2}\;\forall i\in \left[m\right],$$

*the algorithm* ${𝒜}_{\text{FP}}$ *have expected federated stability*

$$E_{{𝒜}_{\text{FP}}}\left[\gamma_{i}\right]\leq C\cdot \frac{\beta \|\ell {\|}_{\infty }}{n_{i}\left(\mu +\lambda \right)},$$

*where* $C_{1},\;C_{2}$ *are two constants only depending on* $\left(\mu ,\;\beta ,\;\|\ell {\|}_{\infty },\;D\right)$, *and* C *is an absolute constant*.

**Proof** By Proposition 21, it suffices to upper bound the error term ${\text{Err}}_{i}$ by a constant multiple of $\frac{\beta \|\ell {\|}_{\infty }}{n_{i}(\mu +\lambda )}$. Invoking Lemma 15, we have

$${\left(E_{{𝒜}_{\text{FP}}}\left[\varepsilon^{\left(\text{global }\right)}\right]\right)}^{2}\leq E_{{𝒜}_{\text{FP}}}\left[{\left(\varepsilon^{\left(\text{global }\right)}\right)}^{2}\right]\leq \frac{12(\lambda +\mu {)}^{2}m\|p{\|}^{2}\left(\beta^{2}D^{2}\wedge 2\lambda \|\ell {\|}_{\infty }\wedge \lambda^{2}D^{2}\right)}{\lambda^{2}\mu^{2}\left(T+1\right)}.$$

This gives

(50)
$$E_{{𝒜}_{\text{FP}}}\left[{\left(\varepsilon^{\left(\text{global}\right)}\right)}^{2}\right]{≲}_{\left(\mu ,\beta ,\|\mu {\|}_{\infty },D\right)}\frac{(\lambda \vee 1{)}^{2}m\|p{\|}^{2}\left(1\wedge \lambda \wedge \lambda^{2}\right)}{\lambda^{2}\left(T+1\right)}≲\frac{m\|p{\|}^{2}}{T+1},$$

where we recall that ${a_{n}≲}_{\left(\mu ,\beta ,\|\mu {\|}_{\infty },D\right)}b_{n}$ means $\left|a_{n}\right|\leq Cb_{n}$ for a constant C that only depending on $\left(\mu ,\beta ,\|\mu {\|}_{\infty },D\right)$, and the last inequality follows from $(\lambda \vee 1{)}^{2}\left(1\wedge \lambda \wedge \lambda^{2}\right)\leq \lambda^{2}$ regardless λ≥1 or λ≤1. Meanwhile, by Lemma 14, we have

$${\left.\left(E_{{𝒜}_{\text{FP}}}\left[\varepsilon^{\left(i\right)}\right)\right]\right)}^{2}\leq E_{{𝒜}_{\text{FP}}}\left[{\left(\varepsilon^{\left(i\right)}\right)}^{2}\right]\leq \frac{8\beta^{2}D^{2}}{\mu^{2}\left(K_{T}+1\right)}≲\left(\beta ,D\right)\frac{1}{K_{T}+1}.$$

Recalling the definition of ${\text{Err}}_{i}$, we have

$$E_{{𝒜}_{\text{FP}}}\left[{\text{Err}}_{i}\right]\;{≲}_{\left(\mu ,\beta ,\|\mu {\|}_{\infty },D\right)}\lambda E_{{𝒜}_{\text{FP}}}\left[\varepsilon^{\left(\text{global }\right)}\right]+\lambda p_{i}E_{{𝒜}_{\text{FP}}}\left[\varepsilon^{\left(i\right)}\right]+\lambda^{2}E_{{𝒜}_{\text{FP}}}\left[{\left(\varepsilon^{\left(\text{global }\right)}\right)}^{2}\right]+p_{i}^{2}\lambda^{2}E_{{𝒜}_{\text{FP}}}\left[{\left(\varepsilon^{\left(i\right)}\right)}^{2}\right]\;{≲}_{\left(\mu ,\beta ,\|\mu {\|}_{\infty },D\right)}\frac{\lambda \sqrt{m}\|p\|}{\sqrt{T+1}}+\frac{\lambda p_{i}}{\sqrt{K_{T}+1}}+\frac{\lambda^{2}m\|p{\|}^{2}}{T+1}+\frac{p_{i}^{2}\lambda^{2}}{K_{T}+1}.$$

Thus, it suffices to require

$$\sqrt{T}≳{\;}_{\left(\mu ,\beta ,\|\mu {\|}_{\infty },D\right)}\lambda \sqrt{m}\|p\|n_{i}(\mu +\lambda ),\;T≳{\;}_{\left(\mu ,\beta ,\|\mu {\|}_{\infty },D\right)}\lambda^{2}m\|p{\|}^{2}n_{i}(\mu +\lambda ),\;\sqrt{K_{T}}≳{\;}_{\left(\mu ,\beta ,\|\mu {\|}_{\infty },D\right)}\lambda p_{i}n_{i}(\mu +\lambda ),\;K_{T}≳{\;}_{\left(\mu ,\beta ,\|\mu {\|}_{\infty },D\right)}p_{i}^{2}\lambda^{2}n_{i}(\mu +\lambda ),$$

which is equivalent to

$$T{≳}_{\left(\mu ,\beta ,\|\mu {\|}_{\infty },D\right)}\text{max}\left\{\lambda^{2}m\|p{\|}^{2}n_{i}^{2}(\lambda \vee 1{)}^{2},\lambda^{2}m\|p{\|}^{2}n_{i}(\lambda \vee 1)\right\}=\lambda^{2}m\|p{\|}^{2}n_{i}^{2}(\lambda \vee 1{)}^{2}$$

$$K_{T}≳\left(\mu ,\beta ,\|\mu {\|}_{\infty },D\right)\text{max}\left\{\lambda^{2}p_{i}^{2}n_{i}^{2}(\mu \vee 1{)}^{2},p_{i}^{2}\lambda^{2}n_{i}(\mu \vee 1)\right\}=\lambda^{2}p_{i}^{2}n_{i}^{2}(\lambda \vee 1{)}^{2},$$

which is exactly (49). ■

Combining the above proposition with Proposition 10, we get the following result.

**Proposition 23 (λ-dependent bound on the AER)** *Let* Assumption A (a, b) *and* (48) *hold. Run* ${𝒜}_{\text{FP}}$ *with hyperparameters chosen as in* Lemma 14 *and* 15. *Then, as long as*

(51)
$$T\;\geq C_{1}\cdot \lambda (\lambda \vee 1)m\|p{\|}^{2}\cdot \left({\left[\sum_{s\in [m]}p_{s}/n_{s}\right]}^{-1}\vee \left[\lambda (\lambda \vee 1)n_{i}^{2}\right]\right),\;K_{T}\;\geq C_{2}\cdot (\lambda +1{)}^{2}\cdot \left({\left[\sum_{s\in [m]}p_{s}/n_{s}\right]}^{-1}\vee \left[\lambda^{2}p_{i}^{2}n_{i}^{2}\right]\right),$$

*for any* i∈[m], *the algorithm* ${𝒜}_{\text{FP}}$ *satisfies*

$$E_{{𝒜}_{\text{FP}},S}\left[{\text{AER}}_{p}\left({𝒜}_{\text{FP}}\right)\right]\leq C\cdot \frac{\beta \|\ell {\|}_{\infty }}{\mu +\lambda }\sum_{i\in \left[m\right]}\frac{p_{i}}{n_{i}}+\frac{\lambda }{2}\sum_{i\in \left[m\right]}p_{i}{∥w_{p}^{\left(\text{global }\right)}-w_{⋆}^{\left(i\right)}∥}^{2},$$

*where* $C_{1},\;C_{2}$ *are two constants only depending on* $\left(\mu ,\beta ,\|\ell {\|}_{\infty },D\right)$, *and* C *is an absolute constant*.

**Proof** In view of Propositions 10 and 22, it suffices to set $T,\;K_{T}$ such that (1) (49) is satisfied; and (2) $E_{{𝒜}_{\text{FP}}}\left[{ℰ}_{\text{OPT}}\right]$ is upper bounded by a constant multiple of $\frac{\beta \|\ell {\|}_{\infty }}{\mu +\lambda }\sum_{i\in [m]}\frac{p_{i}}{n_{i}}$. To achive the second goal, note that by Proposition 16, the optimization error is bounded by

(52)
$$E_{{𝒜}_{\text{FP}}}\left[{ℰ}_{\text{OPT}}\right]{≲}_{\left(\mu ,\beta ,\|\mu {\|}_{\infty },D\right)}\frac{\lambda \vee 1}{K_{T}+1}+\frac{\lambda m\|p{\|}^{2}}{T+1}.$$

Thus, it suffices to require ${T≳}_{\left(\mu ,\beta ,\|\mu {\|}_{\infty },D\right)}\frac{\lambda (\lambda \vee 1)m\|p{\|}^{2}}{\sum_{i\in [m]}p_{i}/n_{i}}$ and ${K_{T}≳}_{\left(\mu ,\beta ,\|\mu {\|}_{\infty },D\right)}\frac{(\lambda \vee 1{)}^{2}}{\sum_{i\in [m]}p_{i}/n_{i}}$. This requirement, combined with (49), is exactly (51). ■

With the above proposition at hand, we are ready to give our proof of Theorem 11.

**Proof** [Proof of Theorem 11] We first define the following three events:

$$\text{A}≔\left\{R\geq \sqrt{\sum_{i\in [m]}\frac{p_{i}}{n_{i}}}\right\},\text{B}≔\left\{\frac{\sum_{i\in [m]}p_{i}^{2}/n_{i}}{\sqrt{\sum_{i\in [m]}p_{i}/n_{i}}}\leq R\leq \sqrt{\sum_{i\in [m]}\frac{p_{i}}{n_{i}}}\right\},\text{C}≔\left\{R\leq \frac{\sum_{i\in [m]}p_{i}^{2}/n_{i}}{\sqrt{\sum_{i\in [m]}p_{i}/n_{i}}}\right\}.$$

We then choose λ to be

$$\lambda =\frac{\mu }{16R^{2}}\sum_{i\in [m]}\frac{p_{i}}{n_{i}}\cdot 1_{\text{A}}+\frac{\mu }{16C_{p}R}\sqrt{\sum_{i\in [m]}\frac{p_{i}}{n_{i}}}\cdot 1_{\text{B}}+\frac{\mu }{16C_{p}\sum_{i\in [m]}p_{i}^{2}/n_{i}}\sum_{i\in [m]}\frac{p_{i}}{n_{i}}\cdot 1_{\text{C}}.$$

We now consider the three events separately.

1. If A holds, then $p_{i}\lambda =\frac{p_{i}\mu }{16R^{2}}\sum_{i\in [m]}\frac{p_{i}}{\mu_{i}}\leq \frac{p_{i}\mu }{16}\leq \frac{\mu }{16}$. Thus we can invoke Proposition 23 to get
  $$E_{{𝒜}_{\text{FP}},S}\left[{\text{AR}}_{p}\right]\leq \left(\frac{C\beta ∥\ell {∥}_{\infty }}{\mu }+\frac{\mu }{32}\right)\sum_{i\in \left[m\right]}\frac{p_{i}}{n_{i}}≲\text{right-hand side of }(28).$$
2. If B holds, then $p_{i}\lambda =\frac{p_{i}\mu }{16C_{p}R}\sqrt{\sum_{i\in [m]}\frac{p_{i}}{n_{i}}}\leq \frac{p_{\text{max}}\mu N}{16C_{p}\sum_{i\in [m]}p_{i}^{2}/n_{i}}\sum_{i\in [m]}\frac{p_{i}}{n_{i}}\leq \frac{\mu }{16}$, where the last inequality is by the definition of $C_{p}$. Hence, by Proposition 23, we have
  $$E_{{𝒜}_{\text{FP}},S}\left[{\text{AER}}_{p}\right]\leq \left(\frac{16CC_{p}\beta ∥\ell {∥}_{\infty }}{\mu }+\frac{\mu }{32C_{p}}\right)R\sqrt{\sum_{i\in [m]}\frac{p_{i}}{n_{i}}}≲\text{right-hand side of }(28).$$
3. If C holds, then $p_{i}\lambda =\frac{p_{i}\mu }{16C_{p}\sum_{i\in [m]}p_{i}^{2}/n_{i}}\sum_{i\in [m]}\frac{p_{i}}{n_{i}}\leq \frac{\mu }{16}$, and thus Proposition 23 gives
  $$E_{{𝒜}_{\text{FP}},S}\left[{\text{AER}}_{p}\right]\leq \left(\frac{16CC_{p}\beta ∥\ell {∥}_{\infty }}{\mu }+\frac{\mu }{32C_{p}}\right)\sum_{i\in [m]}\frac{p_{i}^{2}}{n_{i}}≲\text{right-hand side of }(28).$$

The desired result follows by combining the above three cases together. ■

#### C.3.1 Proof of Proposition 21: Stability of Approximate Minimizers

We first present two lemmas, from which Proposition 21 will follow.

**Lemma 24 (Federated stability of approximate minimizers, Part I)** *Let* Assumption A(b) *holds, and consider an algorithm* $𝒜=\left({\hat{w}}^{\text{(global)}},{\left\{{\hat{w}}^{\left(i\right)}\right\}}_{1}^{m}\right)$ *that satisfies the following conditions*:

1. there exist positive constants $\delta^{\text{(global)}},\;\zeta^{\text{(global)}}$ such that
  (53)
  $$\sum_{i\in [m]}p_{i}F_{i}\left({\hat{w}}^{\left(\text{global}\right)},S_{i}\right)\leq \delta^{\left(\text{global}\right)}+\sum_{i\in [m]}p_{i}F_{i}\left({\tilde{w}}^{\text{(global)}},S_{i}\right),$$
  (54)
  $$∥\sum_{i\in [m]}p_{i}\nabla F_{i}\left({\hat{w}}^{\text{(global)}},S_{i}\right)∥\leq \zeta^{\text{(global)}}.$$
2. *for any* i∈[m]*, there exist positive constants* ${\left\{\delta^{\left(i\right)},\zeta^{\left(i\right)},\varepsilon^{\left(i\right)}\right\}}_{i=1}^{m}$ *such that*
  (55)
  $$L_{i}\left({\hat{w}}^{\left(i\right)},S_{i}\right)+\frac{\lambda }{2}{∥{\hat{w}}^{\left(\text{global}\right)}-{\hat{w}}^{\left(i\right)}∥}^{2}\leq \delta^{\left(i\right)}+F_{i}\left({\hat{w}}^{\left(\text{global}\right)},S_{i}\right),$$
  (56)
  $$∥\nabla L_{i}\left({\hat{w}}^{\left(i\right)},S_{i}\right)+\lambda \left({\hat{w}}^{\left(i\right)}-{\hat{w}}^{\left(\text{global}\right)}\right)∥\leq \zeta^{\left(i\right)},$$
  (57)
  $$∥{\hat{w}}^{\left(i\right)}-{\text{Prox}}_{L_{i}/\lambda }\left({\hat{w}}^{\left(\text{global}\right)}\right)∥\leq \varepsilon^{\left(i\right)}.$$

*Assume in addition that* (48) *holds. Then* 𝒜 *has federated stability*

(58)
$$\gamma_{i}\leq \frac{160\beta \|\ell {\|}_{\infty }}{n_{i}\left(\mu +\lambda \right)}+\sqrt{2\beta \|\ell {\|}_{\infty }}\cdot {ℰ}_{\lambda ,i}+\beta {ℰ}_{\lambda ,i}^{2},$$

*where*

(59)
$${ℰ}_{\lambda ,i}≔\frac{8\zeta^{\left(i\right)}}{\mu +\lambda }+\sqrt{\frac{8\delta^{\left(i\right)}}{\mu +\lambda }}+8\mu^{-1}\left(2\zeta^{\left(\text{global}\right)}+4p_{i}\lambda \varepsilon^{\left(i\right)}+\sqrt{\frac{2\mu \lambda \delta^{\left(\text{global}\right)}}{\mu +\lambda }}\right)$$

*is the error term due to not exactly minimizing* (20).

**Lemma 25 (Federated stability of approximate minimizers, Part II)** Let Assumption A(b) holds and consider an algorithm $𝒜=\left({\hat{w}}^{\left(\text{global}\right)},{\left\{{\hat{w}}^{\left(i\right)}\right\}}_{1}^{m}\right)$ *that produces an* $\left(\varepsilon^{\left(\text{global}\right)},{\left\{\varepsilon^{\left(i\right)}\right\}}_{1}^{m}\right)$-*minimizer in the sense of* Definition 20. *Then* 𝒜 *also satisfies* Equations (53)–(57) *with*

$$\delta^{\left(\text{global }\right)}=\frac{\lambda }{2}\varepsilon^{\left(\text{global }\right)},\;\zeta^{\left(\text{global }\right)}=\lambda \varepsilon^{\left(\text{global }\right)},\;\delta^{\left(i\right)}=\frac{\beta +\lambda }{2}\varepsilon^{\left(i\right)},\;\zeta^{\left(i\right)}=(\beta +\lambda )\varepsilon^{\left(i\right)}.$$

**Proof** These correspondences are consequences of λ-smoothness of $F_{i}$ and (β+λ)-smoothness of $L_{i}\left(\cdot ,S_{i}\right)+\frac{\lambda }{2}{∥{\hat{w}}^{\text{(global)}}-\cdot ∥}^{2}$. We omit the details. ■

With the above two lemmas at hand, the proof of Proposition 21 is purely computational:

**Proof** [Proof of Proposition 21 given Lemma 24 and 25] Invoking 25, the error term ${ℰ}_{\lambda ,i}$ defined in Equation 59 can be bounded above by

$${ℰ}_{\lambda ,i}\;\leq \frac{8(\beta +\lambda )}{\mu +\lambda }\cdot \varepsilon^{\left(\text{global }\right)}+\sqrt{\frac{4(\beta +\lambda )}{\mu +\lambda }}\cdot \varepsilon^{\left(i\right)}+\frac{8}{\mu }\left(2\lambda \varepsilon^{\left(\text{global }\right)}+4p_{i}\lambda \varepsilon^{\left(i\right)}+\sqrt{\frac{\mu \lambda^{2}}{\mu +\lambda }}\right)\;=8\varepsilon^{\left(\text{global }\right)}\left(\frac{\beta +\lambda }{\mu +\lambda }+\frac{2\lambda }{\mu }+\frac{\lambda }{\sqrt{\mu (\mu +\lambda )}}\right)+2\varepsilon^{\left(i\right)}\left(\sqrt{\frac{\beta +\lambda }{\mu +\lambda }}+\frac{16p_{i}\lambda }{\mu }\right)\;\leq 8\varepsilon^{\left(\text{global }\right)}\left(\frac{\beta +\lambda }{\mu +\lambda }+\frac{3\lambda }{\mu }\right)+2\varepsilon^{\left(i\right)}\left(\sqrt{\frac{\beta +\lambda }{\mu +\lambda }}+\frac{16p_{i}\lambda }{\mu }\right).$$

This gives

$${ℰ}_{\lambda ,i}^{2}\leq 128{\left(\varepsilon^{\left(\text{global }\right)}\right)}^{2}{\left(\frac{\beta +\lambda }{\mu +\lambda }+\frac{3\lambda }{\mu }\right)}^{2}+8{\left(\varepsilon^{\left(i\right)}\right)}^{2}{\left(\sqrt{\frac{\beta +\lambda }{\mu +\lambda }}+\frac{16p_{i}\lambda }{\mu }\right)}^{2}.$$

Plugging the above two displays to (58) gives the desired result. ■

We now present our proof of Lemma 24. We start by stating and proving several useful lemmas.

**Lemma 26 (From loss stability to parameter stability)** *Let* Assumption A(b) *holds. Then the algorithm* $𝒜=\left({\hat{w}}^{\text{(global)}},\left\{{\hat{w}}^{\left(i\right)}\right\}\right)$ *has federated stability*

$$\gamma_{i}\leq \sqrt{2\beta \|\ell {\|}_{\infty }}\cdot ∥{\hat{w}}^{\left(i\right)}\left(S\right)-{\hat{w}}^{\left(i\right)}\left(S^{\setminus \left(i,j_{i}\right)}\right)∥+\frac{\beta }{2}{∥{\hat{w}}^{\left(i\right)}\left(S\right)-{\hat{w}}^{\left(i\right)}\left(S^{\setminus \left(i,j_{i}\right)}\right)∥}^{2}.$$

**Proof** This lemma has implicitly appeared in the proofs of many stability-based generalization bounds (see, e.g., Section 13.3.2 of Shalev-Shwartz and Ben-David (2014)), and we provide a proof for completeness. By β-smoothness, for an arbitrary z∈𝒵 we have

$$\ell \left({\hat{w}}^{\left(i\right)}(S),z\right)-\ell \left({\hat{w}}^{\left(i\right)}\left(S^{\setminus \left(i,j_{i}\right)}\right),z\right)\;\leq \left\langle \nabla \ell \left({\hat{w}}^{\left(i\right)}\left(S^{\setminus \left(i,j_{i}\right)}\right),z\right),{\hat{w}}^{\left(i\right)}(S)-{\hat{w}}^{\left(i\right)}\left(S^{\setminus \left(i,j_{i}\right)}\right)\right\rangle +\frac{\beta }{2}{∥{\hat{w}}^{\left(i\right)}(S)-{\hat{w}}^{\left(i\right)}\left(S^{\setminus \left(i,j_{i}\right)}\right)∥}^{2}\;\leq ∥\nabla \ell \left({\hat{w}}^{\left(i\right)}\left(S^{\setminus \left(i,j_{i}\right)}\right),z\right)∥\cdot ∥{\hat{w}}^{\left(i\right)}(S)-{\hat{w}}^{\left(i\right)}\left(S^{\setminus \left(i,j_{i}\right)}\right)∥+\frac{\beta }{2}{∥{\hat{w}}^{\left(i\right)}(S)-{\hat{w}}^{\left(i\right)}\left(S^{\setminus \left(i,j_{i}\right)}\right)∥}^{2}\;\leq \sqrt{2\beta \left(\ell \left({\hat{w}}^{\left(i\right)}\left(S^{\setminus \left(i,j_{i}\right)}\right),z\right)-\underset{w^{\left(i\right)}\in 𝒲}{\text{min}}\ell \left(w^{\left(i\right)},z\right)\right)}\cdot ∥{\hat{w}}^{\left(i\right)}(S)-{\hat{w}}^{\left(i\right)}\left(S^{\setminus \left(i,j_{i}\right)}\right)∥+\frac{\beta }{2}{∥{\hat{w}}^{\left(i\right)}(S)-{\hat{w}}^{\left(i\right)}\left(S^{\setminus \left(i,j_{i}\right)}\right)∥}^{2}\;\leq \sqrt{2\beta \|\ell {\|}_{\infty }}\cdot ∥{\hat{w}}^{\left(i\right)}(S)-{\hat{w}}^{\left(i\right)}\left(S^{\setminus \left(i,j_{i}\right)}\right)∥+\frac{\beta }{2}{∥{\hat{w}}^{\left(i\right)}(S)-{\hat{w}}^{\left(i\right)}\left(S^{\setminus \left(i,j_{i}\right)}\right)∥}^{2},$$

where the last inequality follows from boundedness of ℓ. By a nearly identical argument, the above upper bound also holds for $-\ell \left({\hat{w}}^{\left(i\right)}(S),z\right)+\ell \left({\hat{w}}^{\left(i\right)}\left(S^{\setminus \left(i,j_{i}\right)}\right),z\right)$, and the desired result follows. ■

**Lemma 27 (Local stability implies global stability)** *Assume* Assumption A(b) *holds and consider an algorithm* $𝒜=\left({\hat{w}}^{\text{(global)}},{\left\{{\hat{w}}^{\left(i\right)}\right\}}_{1}^{m}\right)$ *that satisfies* Equations (53), (54) *and* (57). *Then for any* i∈[m], $j_{i}\in \left[n_{i}\right]$, *we have*

(60)
$$∥{\hat{w}}^{\text{(global)}}\left(S^{\setminus \left(i,j_{i}\right)}\right)-{\hat{w}}^{\text{(global)}}(S)∥\;\leq \frac{\lambda +\mu }{\lambda \mu }\left(2\zeta^{\left(\text{global}\right)}+\sqrt{\frac{2\lambda \mu \delta^{\left(\text{global}\right)}}{\lambda +\mu }}+4p_{i}\lambda \varepsilon^{\left(i\right)}+2p_{i}\lambda ∥{\hat{w}}^{\left(i\right)}\left(S^{\setminus \left(i,j_{1}\right)}\right)-{\hat{w}}^{\left(i\right)}\left(S\right)∥\right).$$

**Proof** Without loss of generality we consider the first client. Let $\mu_{F}$ be the strongly convex constant of $\sum_{i}p_{i}F_{i}$, which, by Lemma 17, is equal to $\sum_{i}p_{i}\cdot \frac{\mu \lambda }{\mu +\lambda }=\lambda \mu /(\lambda +\mu )$. Now, by strong convexity, we have

$$\frac{\mu_{F}}{2}{∥{\hat{w}}^{\left(\text{global }\right)}(S)-{\hat{w}}^{\text{(global) }}\left(S^{\setminus \left(1,j_{1}\right)}\right)∥}^{2}\;\leq \sum_{i\in [m]}p_{i}\left(F_{i}\left({\hat{w}}^{\left(\text{global }\right)}\left(S^{\setminus \left(1,j_{1}\right)}\right),S_{i}\right)-F_{i}\left({\hat{w}}^{\text{(global) }}(S),S_{i}\right)\right)+\left\langle \sum_{i\in [m]}p_{i}\nabla F_{i}\left({\hat{w}}^{\text{(global) }}(S),S_{i}\right),{\hat{w}}^{\text{(global) }}\left(S^{\setminus \left(1,j_{1}\right)}-{\hat{w}}^{\text{(global) }}(S)\right)\right\rangle \;\overset{\left(54\right)}{\leq }\sum_{i\in [m]}p_{i}\left(F_{i}\left({\hat{w}}^{\text{(global) }}\left(S^{\setminus \left(1,j_{1}\right)}\right),S_{i}\right)-F_{i}\left({\hat{w}}^{\text{(global) }}(S),S_{i}\right)\right)+\zeta^{\left(\text{global }\right)}∥{\hat{w}}^{\left(\text{global }\right)}\left(S^{\setminus \left(1,j_{1}\right)}\right)-{\hat{w}}^{\left(\text{global }\right)}(S)∥\;=\left(p_{1}F_{1}\left({\hat{w}}^{\text{(global) }}\left(S^{\setminus \left(1,j_{1}\right)}\right),S_{1}^{\setminus j_{1}}\right)+\sum_{i\neq 1}p_{i}F_{i}\left({\hat{w}}^{\text{(global) }}\left(S^{\setminus \left(1,j_{1}\right)}\right),S_{i}\right)\right)-\left(p_{1}F_{1}\left({\hat{w}}^{\text{(global) }}\left(S,S_{1}^{\setminus j_{1}}\right)+\sum_{i\neq 1}p_{i}F_{i}\left({\hat{w}}^{\text{(global) }}\left(S,S_{i}\right)\right)\right.\right.+p_{1}\left(F_{1}\left({\hat{w}}^{\text{(global) }}\left(S^{\setminus \left(1,j_{1}\right)}\right),S_{1}\right)-F_{1}\left({\hat{w}}^{\text{(global) }}\left(S^{\setminus \left(1,j_{1}\right)}\right),S_{1}^{\setminus j_{1}}\right)\right.\left.+F_{1}\left({\hat{w}}^{\text{(global) }}(S),S_{1}^{\setminus j_{1}}\right)-F_{1}\left({\hat{w}}^{\text{(global) }}(S),S_{1}\right)\right)+\zeta^{\text{(global) }}∥{\hat{w}}^{\text{(global) }}\left(S^{\setminus \left(1,j_{1}\right)}\right)-{\hat{w}}^{\text{(global) }}(S)∥\;\overset{\left(53\right)}{\leq }\delta^{\left(\text{global }\right)}+\zeta^{\text{(global) })}∥{\hat{w}}^{\text{(global) }}\left(S^{\setminus \left(1,j_{1}\right)}\right)-{\hat{w}}^{\text{(global) }}(S)∥+p_{1}\left(F_{1}\left({\hat{w}}^{\text{(global) }}\left(S^{\setminus \left(1,j_{1}\right)}\right),S_{1}\right)-F_{1}\left({\hat{w}}^{\text{(global) }}(S),S_{1}\right)\right.\left.\;+F_{1}\left({\hat{w}}^{\text{(global) }}(S),S_{1}^{\setminus j_{1}}\right)-F_{1}\left({\hat{w}}^{\text{(global) })}\left(S^{\setminus \left(1,j_{1}\right)}\right),S_{1}^{\setminus j_{1}}\right)\right).$$

Since $F_{1}$ is λ-smooth by Lemma 17, we can proceed by

$$\frac{\mu_{F}}{2}{∥{\hat{w}}^{\text{(global) }}(S)-{\hat{w}}^{\text{(global) }}\left(S^{\setminus \left(1,j_{1}\right)}\right)∥}^{2}\;\leq \delta^{\text{(global) }}+\zeta^{\text{(global) }}∥{\hat{w}}^{\text{(global) }}\left(S^{\setminus \left(1,j_{1}\right)}\right)-{\hat{w}}^{\text{(global) }}(S)∥+p_{1}\lambda {∥{\hat{w}}^{\text{(global) }}\left(S^{\setminus \left(1,j_{1}\right)}\right)-{\hat{w}}^{\text{(global) }}(S)∥}^{2}+p_{1}\left\langle \nabla F_{1}\left({\hat{w}}^{\text{(global) }}(S),S_{1}\right)-\nabla F_{1}\left({\hat{w}}^{\text{(global) }}\left(S^{\setminus \left(1,j_{1}\right)}\right),S_{1}^{\setminus j_{1}}\right),{\hat{w}}^{\text{(global) }}\left(S^{\setminus \left(1,j_{1}\right)}\right)-{\hat{w}}^{\text{(global) }}(S)\right\rangle .$$

Since $\nabla F_{1}\left(w^{\left(\text{global}\right)},S_{1}\right)=\lambda \left(w^{\text{(global)}}-{\text{Prox}}_{L_{1}/\lambda }\left(w^{\text{(global)}},S_{1}\right)\right)$, with some algebra, the right-hand side above is in fact equal to

$$\delta^{\text{(global) }}+\zeta^{\text{(global) }}∥{\hat{w}}^{\text{(global) }}\left(S^{\setminus \left(1,j_{1}\right)}\right)-{\hat{w}}^{\text{(global) }}(S)∥+p_{1}\lambda \left\langle {\hat{w}}^{\left(1\right)}(S)-{\text{Prox}}_{L_{1}/\lambda }\left({\hat{w}}^{\left(\text{global }\right)}(S),S_{1}\right),{\hat{w}}^{\left(\text{global }\right)}\left(S^{\setminus \left(1,j_{1}\right)}\right)-{\hat{w}}^{\left(\text{global }\right)}(S)\right\rangle +p_{1}\lambda \left\langle {\text{Prox}}_{L_{1}/\lambda }\left({\hat{w}}^{\left(\text{global }\right)}\left(S^{\setminus \left(1,j_{1}\right)}\right),S_{1}^{\setminus j_{1}}\right)-{\hat{w}}^{\left(1\right)}\left(S^{\setminus \left(1,j_{1}\right)},{\hat{w}}^{\left(\text{global }\right)}\left(S^{\setminus \left(1,j_{1}\right)}\right)-{\hat{w}}^{\left(\text{global }\right)}(S)\right\rangle \right.+p_{1}\lambda \left\langle {\hat{w}}^{\left(1\right)}\left(S^{\setminus \left(1,j_{1}\right)}\right)-{\hat{w}}^{\left(1\right)}(S),{\hat{w}}^{\text{(global) }}\left(S^{\setminus \left(1,j_{1}\right)}\right)-{\hat{w}}^{\text{(global) }}(S)\right\rangle \;\overset{\text{ (57) }}{\leq }\delta^{\text{(global) }}+\left(\zeta^{\text{(global) }}+2p_{1}\lambda \right)∥{\hat{w}}^{\text{(global) }}\left(S^{\setminus \left(1,j_{1}\right)}\right)-{\hat{w}}^{\text{(global) }}(S)∥+p_{1}\lambda ∥{\hat{w}}^{\left(i\right)}\left(S^{\setminus \left(1,j_{1}\right)}\right)-{\hat{w}}^{\left(i\right)}(S)∥∥{\hat{w}}^{\left(\text{global }\right)}\left(S^{\setminus \left(1,j_{1}\right)}\right)-{\hat{w}}^{\text{(global) }}(S)∥.$$

The above bound gives a quadratic inequality: if we let $s_{G}≔∥{\hat{w}}^{\text{(global)}}\left(S^{\setminus \left(1,j_{1}\right)}\right)-{\hat{w}}^{\left(\text{global}\right)}(S)∥$ and $s_{1}≔∥{\hat{w}}^{\left(i\right)}\left(S^{\setminus \left(1,j_{1}\right)}\right)-{\hat{w}}^{\left(i\right)}(S)∥$, then the above bound can be written as

$$\frac{\mu_{F}}{2}\cdot {\text{s}}_{G}^{2}-\left(\zeta^{\left(\text{global }\right)}+2p_{1}\lambda \varepsilon^{\left(1\right)}+p_{1}\lambda {\text{s}}_{1}\right)\cdot {\text{s}}_{G}-\delta^{\left(\text{global }\right)}\leq 0.$$

Solving this inequality gives

$${\text{s}}_{G}\;\leq \frac{1}{\mu_{F}}\cdot \left[\zeta^{\left(\text{global}\right)}+2p_{1}\lambda \varepsilon^{\left(1\right)}+p_{1}\lambda {\text{s}}_{1}+\sqrt{{\left(\zeta^{\text{(global)}}+2p_{1}\lambda \varepsilon^{\left(1\right)}+p_{1}\lambda {\text{s}}_{1}\right)}^{2}+2\mu_{F}\delta^{\text{(global)}}}\right]\;\leq \frac{1}{\mu_{F}}\left(2\zeta^{\text{(global)}}+4p_{1}\lambda \varepsilon^{\left(1\right)}+2p_{1}\lambda {\text{s}}_{1}+\sqrt{2\mu_{F}\delta^{\left(\text{global}\right)}}\right),$$

which is exactly (60). ■

**Lemma 28 (Parameter stability)** *Under the same assumptions as* Proposition 21, *for any* i∈[m], $j_{i}\in \left[n_{i}\right]$, *we have*

$$∥{\hat{w}}^{\left(i\right)}\left(S^{\setminus \left(i,j_{i}\right)}\right)-{\hat{w}}^{\left(i\right)}(S)∥\leq \frac{16\sqrt{2\beta \|\ell {\|}_{\infty }}}{n_{i}\left(\mu +\lambda \right)}+{ℰ}_{\lambda ,i}.$$

**Proof** Without loss of generality we consider the first client. Since $L_{1}\left(\cdot ,S_{1}\right)+\frac{\lambda }{2}\|{\hat{w}}^{\text{(global)}}(S)-\cdot {\|}^{2}$ is (μ+λ)-strongly convex, we have

$$\frac{1}{2}(\mu +\lambda ){∥{\hat{w}}^{\left(1\right)}(S)-{\hat{w}}^{\left(1\right)}\left(S^{\setminus \left(1,j_{1}\right)}\right)∥}^{2}\;\leq \left(L_{1}\left({\hat{w}}^{\left(1\right)}\left(S^{\setminus \left(1,j_{1}\right)}\right),S_{1}\right)+\frac{\lambda }{2}{∥{\hat{w}}^{\left(\text{global }\right)}\left(S\right)-{\hat{w}}^{\left(1\right)}\left(S^{\setminus \left(1,j_{1}\right)}\right)∥}^{2}\right)-\left(L_{1}\left({\hat{w}}^{\left(1\right)}\left(S\right),S_{1}\right)+\frac{\lambda }{2}{∥{\hat{w}}^{\left(\text{global }\right)}\left(S\right)-{\hat{w}}^{\left(1\right)}\left(S\right)∥}^{2}\right)+\left\langle \nabla L_{1}\left({\hat{w}}^{\left(1\right)}\left(S\right),S_{1}\right)+\lambda \left({\hat{w}}^{\left(1\right)}\left(S\right)-{\hat{w}}^{\left(\text{global }\right)}\left(S\right)\right),{\hat{w}}^{\left(1\right)}\left(S^{\setminus \left(1,j_{1}\right)}\right)-{\hat{w}}^{\left(1\right)}\left(S\right)\right\rangle \;\overset{\left(56\right)}{\leq }\left(L_{1}\left({\hat{w}}^{\left(1\right)}\left(S^{\setminus \left(1,j_{1}\right)}\right),S_{1}^{\setminus j_{1}}\right)+\frac{\lambda }{2}{∥{\hat{w}}^{\left(\text{global }\right)}\left(S^{\setminus \left(1,j_{1}\right)}\right)-{\hat{w}}^{\left(1\right)}\left(S^{\setminus \left(1,j_{1}\right)}\right)∥}^{2}\right)-\left(L_{1}\left({\hat{w}}^{\left(1\right)}(S),S_{1}^{\setminus j_{1}}\right)+\frac{\lambda }{2}{∥{\hat{w}}^{\left(\text{global }\right)}\left(S^{\setminus \left(1,j_{1}\right)}\right)-{\hat{w}}^{\left(1\right)}(S)∥}^{2}\right)-\frac{1}{n_{1}}\ell \left({\hat{w}}^{\left(1\right)}\left(S^{\setminus \left(1,j_{1}\right)}\right),z_{1,j_{1}}^{\prime }\right)+\frac{1}{n_{1}}\ell \left({\hat{w}}^{\left(1\right)}\left(S^{\setminus \left(1,j_{1}\right)}\right),z_{j_{1}}^{\left(1\right)}\right)+\frac{1}{n}\ell \left({\hat{w}}^{\left(1\right)}(S),z_{1,j_{1}}^{\prime }\right)-\frac{1}{n_{1}}\ell \left({\hat{w}}^{\left(S\right)},z_{j_{1}}^{\left(1\right)}\right)-\frac{\lambda }{2}{∥{\hat{w}}^{\left(\text{global }\right)}\left(S^{\setminus \left(1,j_{1}\right)}\right)-{\hat{w}}^{\left(1\right)}\left(S^{\setminus \left(1,j_{1}\right)}\right)∥}^{2}+\frac{\lambda }{2}{∥{\hat{w}}^{\left(\text{global }\right)}(S)-{\hat{w}}^{\left(1\right)}\left(S^{\setminus \left(1,j_{1}\right)}\right)∥}^{2}+\frac{\lambda }{2}{∥{\hat{w}}^{\left(\text{global }\right)}\left(S^{\setminus \left(1,j_{1}\right)}\right)-{\hat{w}}^{\left(1\right)}(S)∥}^{2}-\frac{\lambda }{2}{∥{\hat{w}}^{\left(\text{global }\right)}(S)-{\hat{w}}^{\left(1\right)}(S)∥}^{2}+\zeta^{\left(1\right)}∥{\hat{w}}^{\left(1\right)}\left(S^{\left(1,j_{1}\right)}\right)-{\hat{w}}^{\left(1\right)}(S)∥\;\overset{\left(55\right)}{\leq }\delta^{\left(1\right)}+\zeta^{\left(1\right)}∥{\hat{w}}^{\left(1\right)}\left(S^{\setminus \left(1,j_{1}\right)}\right)-{\hat{w}}^{\left(1\right)}(S)∥+\lambda \left\langle {\hat{w}}^{\left(\text{global }\right)}(S)-{\hat{w}}^{\left(\text{global }\right)}\left(S^{\setminus \left(1,j_{1}\right)}\right),{\hat{w}}^{\left(S\right)}-{\hat{w}}^{\left(1\right)}\left(S^{\setminus \left(1,j_{1}\right)}\right)\right\rangle +\frac{1}{n_{1}}\left(\ell \left({\hat{w}}^{\left(1\right)}(S),z_{1,j_{1}}^{\prime }\right)-\ell \left({\hat{w}}^{\left(1\right)}\left(S^{\setminus \left(1,j_{1}\right)}\right),z_{1,j_{1}}^{\prime }\right)+\ell \left({\hat{w}}^{\left(1\right)}\left(S^{\setminus \left(1,j_{1}\right)}\right),z_{j_{1}}^{\left(1\right)}\right)-\ell \left({\hat{w}}^{\left(1\right)}(S),z_{j_{1}}^{\left(1\right)}\right)\right)\;\leq \delta^{\left(1\right)}\;+\zeta^{\left(1\right)}∥{\hat{w}}^{\left(1\right)}\left(S^{\setminus \left(1,j_{1}\right)}\right)-{\hat{w}}^{\left(1\right)}(S)∥+\frac{2}{n_{1}}\left(\sqrt{2\beta \|\ell {\|}_{\infty }}∥{\hat{w}}^{\left(1\right)}(S)-{\hat{w}}^{\left(1\right)}\left(S^{\setminus \left(1,j_{1}\right)}\right)∥+\frac{\beta }{2}{∥{\hat{w}}^{\left(1\right)}(S)-{\hat{w}}^{\left(1\right)}\left(S^{\setminus \left(1,j_{1}\right)}\right)∥}^{2}\right)+\frac{\lambda +\mu }{\mu }\left(2\zeta^{\left(\text{global }\right)}+\sqrt{\frac{2\lambda \mu \delta \text{ (global) }}{\lambda +\mu }}+4p_{i}\lambda \varepsilon^{\left(i\right)}+2p_{i}\lambda ∥{\hat{w}}^{\left(i\right)}\left(S^{\setminus \left(i,j_{1}\right)}\right)-{\hat{w}}^{\left(i\right)}(S)∥\right)\times ∥{\hat{w}}^{\left(1\right)}(S)-{\hat{w}}^{\left(1\right)}\left(S^{\setminus \left(1,j_{1}\right)}\right)∥,$$

where the last inequality is by Lemma 26 and Lemma 27. Denoting $s_{1}≔\|{\hat{w}}^{\left(1\right)}(S)-{\hat{w}}^{\left(1\right)}\left(S^{\left.\setminus 1,j_{1}\right)}\right)\|$, the above inequality can be written as

(61)
$$C_{\lambda ,1}{\text{s}}_{1}^{2}-\left[\frac{2\sqrt{2\beta \|\ell {\|}_{\infty }}}{n_{1}}+\zeta^{\left(1\right)}+\frac{\lambda +\mu }{\mu }\left(2\zeta^{\text{(global)}}+4p_{1}\lambda \varepsilon^{\left(1\right)}+\sqrt{\frac{2\lambda \mu \delta^{\text{(global)}}}{\lambda +\mu }}\right)\right]{\text{s}}_{1}-\delta^{\left(1\right)}\leq 0$$

where

$$C_{\lambda ,1}≔\frac{1}{2}\left(\mu +\lambda \right)-\frac{\beta }{n_{1}}-\frac{2p_{1}\lambda \left(\lambda +\mu \right)}{\mu }.$$

By (48), we have

$$C_{\lambda ,1}\geq \frac{\mu +\lambda }{2}-\frac{\mu }{4}-\frac{2p_{1}\lambda (\lambda +\mu )}{\mu }\geq \frac{\lambda +\mu }{4}-\frac{2p_{1}\lambda (\lambda +\mu )}{\mu }=\frac{\lambda +\mu }{4}\cdot \left(1-\frac{8p_{1}\lambda }{\mu }\right)\geq \frac{\lambda +\mu }{8}.$$

In particular, $C_{\lambda ,1}>0$, and thus we can solve the quadratic inequality (61) (similar to the proof of Lemma 27) to get

$$s_{1}\leq \frac{2\sqrt{2\beta \|\ell {\|}_{\infty }}}{C_{\lambda ,1}n_{1}}+\frac{\zeta^{\left(1\right)}+\frac{\lambda +\mu }{\mu }\left(2\zeta^{\left(\text{global }\right)}+4p_{1}\lambda \varepsilon^{\left(1\right)}+\sqrt{\left.\frac{2\lambda \mu \delta^{\left(\text{global }\right)}}{\lambda +\mu }\right)}\right.}{C_{\lambda ,1}}+\sqrt{\frac{\delta^{\left(1\right)}}{C_{\lambda ,1}}}.$$

Plugging in $C_{\lambda ,1}\geq (\lambda +\mu )/8$ to the above inequality gives the desired result. ■

We are finally ready to present a proof of Lemma 24:

**Proof** [Proof of Lemma 24] Invoking Lemma 26, we have

$$\gamma_{i}\leq \sqrt{2\beta \|\ell {\|}_{\infty }}\cdot \left(\frac{16\sqrt{2\beta \|\ell {\|}_{\infty }}}{n_{i}\left(\mu +\lambda \right)}+{ℰ}_{\lambda ,i}\right)+\frac{\beta }{2}{\left(\frac{16\sqrt{2\beta \|\ell {\|}_{\infty }}}{n_{i}\left(\mu +\lambda \right)}+{ℰ}_{\lambda ,i}\right)}^{2}\;\leq \frac{32\beta \|\ell {\|}_{\infty }}{n_{i}\left(\mu +\lambda \right)}\cdot \left(1+\frac{\beta }{n_{i}\left(\mu +\lambda \right)}\right)+\sqrt{2\beta \|\ell {\|}_{\infty }}\cdot {ℰ}_{\lambda ,i}+\beta {ℰ}_{\lambda ,i}^{2},$$

where in the last line we have used $(a+b{)}^{2}\leq 2a^{2}+2b^{2}$. We finish the proof by noting that $\frac{\beta }{n_{i}(\mu +\lambda )}\leq \frac{\beta }{n_{i}\mu }\leq 4$, where the last inequality is by (48). ■

### C.4 Proof of Theorem 12

Compared to the proof of Theorem 11, we need to additionally control the estimation error of the global model.

**Proposition 29 (Estimation error of the global model)** *Let* Assumptions A(b) and B(b) *hold. Then*

$$E_{S}{∥{\tilde{w}}^{\text{(global) }}-w_{p}^{\left(\text{global }\right)}∥}^{2}\leq \frac{48\beta^{2}\sigma^{2}}{\mu^{2}\lambda^{2}}{\left(\sum_{i\in \left[m\right]}\frac{p_{i}}{\sqrt{n_{i}}}\right)}^{2}+\frac{48\beta^{2}R^{2}}{\mu^{2}}+\frac{12(\mu +\lambda {)}^{2}\sigma^{2}}{\mu^{2}\lambda^{2}}\sum_{i\in \left[m\right]}\frac{p_{i}^{2}}{n_{i}}.$$

**Proof** See Appendix C.4.1 ■

With the above proposition, the following result is a counterpart of Proposition 23.

**Proposition 30 (λ-dependent bound on the IER)** *Let* Assumptions A(a, b), B(b) *and Equation* (48) *hold. Run* ${𝒜}_{\text{FP}}$ *with hyperparameters chosen as in* Lemma 14 and 15. *Then, for any* i∈[m], *as long as*

(62)
$$T\;\geq C_{1}\lambda (\lambda \vee 1)m\|p{\|}^{2}n_{i}\cdot \left(p_{i}^{-1}\vee \left[\lambda \left(\lambda \vee 1\right)n_{i}\right]\right),\;\;K_{T}\;\geq C_{2}(\lambda +1{)}^{2}n_{i}\left(p_{i}^{-1}\vee \lambda^{2}p_{i}^{2}n_{i}\right),$$

*the algorithm* ${𝒜}_{\text{FP}}$ *satisfies both*

(63)
$$E_{{𝒜}_{\text{FP}},S}\left[{\text{IER}}_{i}\left({𝒜}_{\text{FP}}\right)\right]\left.\leq \frac{C}{\lambda n_{i}}\left[\beta \|\ell {\|}_{\infty }+\frac{\sigma^{2}\beta^{2}n_{i}}{\mu^{2}}{\left(\sum_{i\in [m]}\frac{p_{i}}{\sqrt{n_{i}}}\right)}^{2}+\sigma^{2}n_{i}\sum_{i\in [m]}\frac{p_{i}^{2}}{n_{i}}\right]+C\lambda \left[\left(1+\frac{\beta^{2}}{\mu^{2}}\right)R^{2}+\frac{\sigma^{2}}{\mu^{2}}\sum_{i\in [m]}\frac{p_{i}^{2}}{n_{i}}\right)\right],$$

*and*

(64)
$$E_{{𝒜}_{\text{FP}},S}\left[{\text{IER}}_{i}\left({𝒜}_{\text{FP}}\right)\right]\leq C\left(\frac{\beta \|\ell {\|}_{\infty }}{\mu n_{i}}+\lambda D^{2}\right),$$

*where* $C_{1},\;C_{2}$ *are two constants only depending on* $\left(\mu ,\beta ,\|\ell {\|}_{\infty },D\right)$, *and* C *is an absolute constant*.

**Proof** Without loss of generality we consider the first client. Our assumptions allow us to invoke Propositions 10 and 22 to get

$$E_{{𝒜}_{\text{FP}},S}\left[{\text{IER}}_{1}\right]\;\leq E_{{𝒜}_{\text{FP}},S}\left[\frac{{ℰ}_{\text{OPT}}}{p_{1}}+\frac{3\lambda }{2}{\left(\varepsilon^{\left(\text{global}\right)}\right)}^{2}+\frac{3\lambda }{2}{∥{\tilde{w}}^{\text{(global)}}-w_{p}^{\left(\text{global}\right)}∥}^{2}+\frac{3\lambda }{2}R^{2}+\frac{320\beta \|\ell {\|}_{\infty }}{n_{1}(\mu +\lambda )}\right]$$

We first show that the expected value of ${ℰ}_{\text{OPT}}/p_{1}$ and $\lambda {\left(\varepsilon^{\text{(global)}}\right)}^{2}$ are both bounded above by a constant multiple of $\frac{\beta \|\ell {\|}_{\infty }}{n_{1}(\mu +\lambda )}$. Indeed, by the estimates we have established in Equations (50) and (52), it suffices to require

$${T≳}_{\left(\mu ,\beta ,\|\mu {\|}_{\infty },D\right)}\lambda \left(\lambda \vee 1\right)m||P||2_{n1},$$

and

$${K_{T}≳}_{\left(\mu ,\beta ,\|\mu {\|}_{\infty },D\right)}\frac{(\lambda \vee 1{)}^{2}n_{1}}{p_{1}},\;{T≳}_{\left(\mu ,\beta ,\|\mu {\|}_{\infty },D\right)}\frac{\lambda \left(\lambda \vee 1\right)m\|p{\|}^{2}n_{1}}{p_{1}},$$

respectively. And the above two displays, combined with (49), is exactly (62). (64) then follows from the compactness of 𝒲. To prove (63), we invoke Proposition 29 to get

$$E_{{𝒜}_{\text{FP}},S}\left[{\text{IER}}_{1}\right]≲\frac{\beta \|\ell {\|}_{\infty }}{n_{1}\left(\mu +\lambda \right)}+\lambda \left(1+\frac{\beta^{2}}{\mu^{2}}\right)R^{2}+\frac{\beta^{2}\sigma^{2}}{\mu^{2}\lambda }{\left(\sum_{i\in \left[m\right]}\frac{p_{i}}{\sqrt{n_{i}}}\right)}^{2}+\frac{(\mu +\lambda {)}^{2}\sigma^{2}}{\mu^{2}\lambda }\sum_{i\in \left[m\right]}\frac{p_{i}^{2}}{n_{i}}\;≲\frac{\beta \|\ell {\|}_{\infty }}{n_{1}\lambda }+\lambda \left(1+\frac{\beta^{2}}{\mu^{2}}\right)R^{2}+\frac{\beta^{2}\sigma^{2}}{\mu^{2}\lambda }{\left(\sum_{i\in \left[m\right]}\frac{p_{i}}{\sqrt{n_{i}}}\right)}^{2}+\left(\frac{\sigma^{2}}{\lambda }+\frac{\lambda \sigma^{2}}{\mu^{2}}\right)\sum_{i\in \left[m\right]}\frac{p_{i}^{2}}{n_{i}},$$

and (64) follows by rearranging terms. ■

We now present our proof of Theorem 12

**Proof** [Proof of Theorem 12| Without loss of generality we consider the first client. Since all $n_{i}\prime$s are of the same order, it suffices to show

(65)
$$E\left[{\text{IER}}_{i}\left({𝒜}_{\text{FP}}\right)\right]≲\left[\left(\mu +\mu^{-1}\right)\left(\beta \|\ell {\|}_{\infty }+\frac{\sigma^{2}\beta^{2}+\beta^{2}+\sigma^{2}}{\mu^{2}}\right)+\mu D^{2}\right]\cdot \left(\frac{R}{\sqrt{N/m}}\wedge \frac{1}{N/m}+\frac{\sqrt{m}}{N}\right)$$

We define the following two events:

$$\text{A}≔\left\{R\geq \sqrt{\frac{m}{N}}\right\},\text{ B}≔{\text{A}}^{c}=\left\{R<\sqrt{\frac{m}{N}}\right\},$$

and we set

$$\lambda =\frac{c_{\text{A}}m}{D^{2}N}\cdot 1_{\text{A}}+c_{\text{B}}\sqrt{\frac{m}{R^{2}N+1}}\cdot 1_{\text{B}},$$

where $c_{\text{A}},\;c_{\text{B}}$ are two constants to be specified later. We consider two cases:

1. If A holds, then from (64) we have $E_{{𝒜}_{\text{FP}},S}\left[{\text{IER}}_{1}\right]≲\left(\frac{\beta \|\ell {\|}_{\infty }}{\mu }+c_{\text{A}}\right)\cdot \frac{1}{N/m}$, provided $\lambda p_{\text{max}}\leq \mu /16$. Note that $\lambda p_{\text{max}}≍\frac{c_{\text{A}}}{D^{2}N}\leq c_{\text{A}}/D^{2}$. So we can choose $\lambda ≍\mu D^{2}$, which gives
  $$E_{{𝒜}_{\text{FP}},S}\left[{\text{IER}}_{1}\right]≲\left(\frac{\beta \|\ell {\|}_{\infty }}{\mu }+\mu D^{2}\right)\cdot \frac{1}{N/m}\leq \text{right-hand side of }(65).$$
2. If **B** holds, and if $\lambda p_{\text{max}}\leq \mu /16$ holds, then from (63) we have
  $$\;E_{{𝒜}_{\text{FP}},S}\left[{\text{IER}}_{1}\right]\;≲\frac{1}{\lambda N/m}\left(\beta \|\ell {\|}_{\infty }+\frac{\sigma^{2}\beta^{2}}{\mu^{2}}+\sigma^{2}\right)+\lambda \left[\left(1+\frac{\beta^{2}}{\mu^{2}}\right)R^{2}+\frac{\sigma^{2}}{\mu^{2}N}\right]\;≲\left(\beta \|\ell {\|}_{\infty }+\frac{\sigma^{2}\beta^{2}+\beta^{2}+\sigma^{2}}{\mu^{2}}\right)\cdot \left(\frac{1}{\lambda N/m}+\lambda \left(R^{2}+N^{-1}\right)\right)\;=\left(\beta \|\ell {\|}_{\infty }+\frac{\sigma^{2}\beta^{2}+\beta^{2}+\sigma^{2}}{\mu^{2}}\right)\cdot \left(\frac{\sqrt{R^{2}N+1}}{c_{\text{B}}N/\sqrt{m}}+\frac{c_{\text{B}}}{N}\sqrt{m\left(R^{2}N+1\right)}\right)\;\leq \left(\beta \|\ell {\|}_{\infty }+\frac{\sigma^{2}\beta^{2}+\beta^{2}+\sigma^{2}}{\mu^{2}}\right)\cdot \left(\frac{R}{c_{\text{B}}\sqrt{N/m}}+\frac{1}{c_{\text{B}}N/\sqrt{m}}+\frac{c_{\text{B}}\sqrt{m}R}{\sqrt{N}}+\frac{c_{\text{B}}\sqrt{m}}{N}\right)\;=\left(\beta \|\ell {\|}_{\infty }+\frac{\sigma^{2}\beta^{2}+\beta^{2}+\sigma^{2}}{\mu^{2}}\right)\cdot \left(c_{\text{B}}+c_{\text{B}}^{-1}\right)\left(\frac{R}{\sqrt{N/m}}+\frac{\sqrt{m}}{N}\right).$$

Note that $p_{\text{max}}\lambda \leq c_{\text{B}}p_{\text{max}}\sqrt{m}≍c_{\text{B}}/\sqrt{m}\leq c_{\text{B}}$. So to satisfy $p_{\text{max}}\lambda \leq \mu /16$, we can choose $c_{\text{B}}≍\mu$. This gives

$$E_{{𝒜}_{\text{FP}},S}\left[{\text{IER}}_{1}\right]\;≲\left(\beta \|\ell {\|}_{\infty }+\frac{\sigma^{2}\beta^{2}+\beta^{2}+\sigma^{2}}{\mu^{2}}\right)\left(\mu +\mu^{-1}\right)\cdot \left(\frac{R}{\sqrt{\frac{N}{m}}}+\frac{\sqrt{m}}{N}\right)\;\leq \text{right-hand side of }(65).$$

The desired result follows by combining the above two cases together. ■

#### C.4.1 Proof of Proposition 29: Estimation Error of the Global Model

We begin by proving a useful lemma.

Lemma 31 (Estimating $w_{⋆}^{\left(i\right)}$ given the knowledge of $w_{p}^{\left(\text{global}\right)}$) *Let* Assumption A(b) *hold. Then for any* i∈[m]*, we have*

$$∥w_{⋆}^{\left(i\right)}-{\text{Prox}}_{L_{i}/\lambda }\left(w_{p}^{\left(\text{global }\right)}\right)∥\leq \frac{2}{\mu +\lambda }∥\nabla L_{i}\left(w_{⋆}^{\left(i\right)},S_{i}\right)+\lambda \left(w_{⋆}^{\left(i\right)}-w_{p}^{\left(\text{global }\right)}\right)∥.$$

**Proof** This follows from an adaptation of the arguments in Theorem 7 of Foster et al. (2019). By strong convexity, we have

$$\left\langle \nabla L_{i}\left(w_{⋆}^{\left(i\right)},S_{i}\right)+\lambda \left(w_{⋆}^{\left(i\right)}-w_{p}^{\left(\text{global }\right)}\right),{\text{Prox}}_{L_{i}/\lambda }\left(w_{p}^{\left(\text{global }\right)}\right)-w_{⋆}^{\left(i\right)}\right\rangle +\frac{\mu +\lambda }{2}{∥w_{⋆}^{\left(i\right)}-{\text{Prox}}_{L_{i}/\lambda }\left(w_{p}^{\left(\text{global }\right)}\right)∥}^{2}\;\leq L_{i}\left(w_{⋆}^{\left(i\right)},S_{i}\right)+\frac{\lambda }{2}{∥w^{\left(\text{global }\right)}-w_{⋆}^{\left(i\right)}∥}^{2}-L_{i}\left({\text{Prox}}_{\frac{L_{i}}{\lambda }}\left(w_{p}^{\left(\text{global }\right)}\right),S_{i}\right)-\frac{\lambda }{2}{∥w_{p}^{\left(\text{global }\right)}-{\text{Prox}}_{\frac{L_{i}}{\lambda }}\left(w_{p}^{\left(\text{global }\right)}\right)∥}^{2}\;\leq 0.$$

If $∥w^{\left(i\right)}-{\text{Prox}}_{L_{i}/\lambda }\left(w_{p}^{\left(\text{global}\right)}\right)∥=0$ we are done. Otherwise, Cauchy-Schwartz inequality applied to the above display gives the desired result. ■

Now, since $\sum_{i\in [m]}p_{i}F_{i}$ is $\mu_{F}=\mu \lambda /(\mu +\lambda )$-strongly convex, we have

$$\left\langle \sum_{i\in [m]}p_{i}\nabla F_{i}\left(w_{p}^{\left(\text{global }\right)}\right),{\tilde{w}}^{\left(\text{global }\right)}-w_{p}^{\left(\text{global }\right)}\right\rangle +\frac{\mu_{F}}{2}{∥{\tilde{w}}^{\left(\text{global }\right)}-w_{p}^{\left(\text{global }\right)}∥}^{2}\;\leq \sum_{i\in [m]}p_{i}F_{i}\left({\tilde{w}}^{\text{(global) })}-\sum_{i\in [m]}p_{i}F_{i}\left(w_{p}^{\left(\text{global }\right)}\right)\right.\;\leq 0$$

If $∥{\tilde{w}}^{\text{(global)}}-w_{p}^{\text{(global)}}∥=0$ we are done. Otherwise, by Cauchy-Schwartz inequality, we get

$$∥{\tilde{w}}^{\left(\text{global }\right)}-w_{p}^{\left(\text{global }\right)}∥\;\leq \frac{2}{\mu_{F}}∥\sum_{i\in \left[m\right]}p_{i}\nabla F_{i}\left(w_{p}^{\left(\text{global }\right)}\right)∥\;=\frac{2}{\mu_{F}}∥\sum_{i\in \left[m\right]}p_{i}\nabla L_{i}\left({\text{Prox}}_{\frac{L_{i}}{\lambda }}\left(w_{p}^{\left(\text{global }\right)}\right),S_{i}\right)∥\;\leq \frac{2}{\mu_{F}}∥\sum_{i\in \left[m\right]}p_{i}\left(\nabla L_{i}\left({\text{Prox}}_{\frac{L_{i}}{\lambda }}\left(w_{p}^{\left(\text{global }\right)}\right),S_{i}\right)-\nabla L_{i}\left(w_{⋆}^{\left(i\right)},S_{i}\right)\right)∥+\frac{2}{\mu_{F}}∥\sum_{i\in \left[m\right]}p_{i}\nabla L_{i}\left(w_{⋆}^{\left(i\right)},S_{i}\right)∥\;\overset{\left(*\right)}{\leq }\frac{2\beta }{\mu_{F}}\sum_{i\in \left[m\right]}p_{i}∥{\text{Prox}}_{\frac{L_{i}}{\lambda }}\left(w_{p}^{\left(\text{global }\right)}\right)-w_{⋆}^{\left(i\right)}∥+\frac{2}{\mu_{F}}∥\sum_{i\in \left[m\right]}p_{i}\nabla L_{i}\left(w_{⋆}^{\left(i\right)},S_{i}\right)∥\;\overset{\left(**\right)}{\leq }\frac{4\beta }{\mu_{F}\left(\mu +\lambda \right)}\sum_{i\in \left[m\right]}p_{i}∥\nabla L_{i}\left(w_{⋆}^{\left(i\right)},S_{i}\right)+\lambda \left(w_{⋆}^{\left(i\right)}-w_{p}^{\left(\text{global }\right)}\right)∥+\frac{2}{\mu_{F}}∥\sum_{i\in \left[m\right]}p_{i}\nabla L_{i}\left(w_{⋆}^{\left(i\right)},S_{i}\right)∥\;\leq \frac{4\beta }{\mu \lambda }\sum_{i\in [m]}p_{i}∥\nabla L_{i}\left(w_{⋆}^{\left(i\right)},S_{i}\right)∥+\frac{4\beta R}{\mu }+\frac{2(\mu +\lambda )}{\mu \lambda }∥\sum_{i\in [m]}p_{i}\nabla L_{i}\left(w_{⋆}^{\left(i\right)},S_{i}\right)∥,$$

where (*) is by smoothness of $L_{i}$ and (**) is by Lemma 31. Thus, we have

(66)
$${∥{\tilde{w}}^{\text{(global)}}-w_{p}^{\text{(global)}}∥}^{2}\;\leq \frac{48\beta^{2}}{\mu^{2}\lambda^{2}}{\left(\sum_{i\in [m]}p_{i}∥\nabla L_{i}\left(w_{⋆}^{\left(i\right)},S_{i}\right)∥\right)}^{2}+\frac{48\beta^{2}R^{2}}{\mu^{2}}+\frac{12(\mu +\lambda {)}^{2}}{\mu^{2}\lambda^{2}}{∥\sum_{i\in [m]}p_{i}\nabla L_{i}\left(w_{⋆}^{\left(i\right)},S_{i}\right)∥}^{2}$$

Note that

$${\left(\sum_{i\in [m]}p_{i}∥\nabla L_{i}\left(w_{⋆}^{\left(i\right)},S_{i}\right)∥\right)}^{2}=\sum_{i\in [m]}p_{i}^{2}{∥\nabla L_{i}\left(w_{⋆}^{\left(i\right)},S_{i}\right)∥}^{2}+\sum_{i\neq s}p_{i}p_{s}∥\nabla L_{i}\left(w_{⋆}^{\left(i\right)},S_{i}\right)∥∥\nabla L_{s}\left(w_{⋆}^{\left(s\right)},S_{s}\right)∥.$$

Taking expectation at both sides, we arrive at

$$E_{S}\left[{\left(\sum_{i\in [m]}p_{i}∥\nabla L_{i}\left(w_{⋆}^{\left(i\right)},S_{i}\right)∥\right)}^{2}\right]\leq \sum_{i\in \left[m\right]}\frac{p_{i}^{2}\sigma^{2}}{n_{i}}+\sum_{i\neq s}\frac{p_{i}p_{s}\sigma_{i}\sigma_{s}}{\sqrt{n_{i}n_{s}}}=\sigma^{2}{\left(\sum_{i\in \left[m\right]}\frac{p_{i}}{\sqrt{n_{i}}}\right)}^{2}.$$

Meanwhile, we have

$$E_{S}{∥\sum_{i\in [m]}p_{i}\nabla L_{i}\left(w_{⋆}^{\left(i\right)},S_{i}\right)∥}^{2}\leq \sum_{i\in \left[m\right]}\frac{p_{i}^{2}\sigma^{2}}{n_{i}}.$$

The desired result follows by plugging the previous two displays to (66).

## D. Details on Experiments

In each round (among 100 rounds) of simulation, we first generate $w_{⋆}\in R^{100}$ with i.i.d. standard Gaussian entries, and we set each local model $w_{⋆}^{\left(i\right)}=w_{⋆}+R\cdot v_{i}$, where $v_{i}\in R^{100}$ is a random unit vector that has negative correlation with $w_{⋆}$ and we vary R from 0 to 20. The dataset for the i-th client is then generated by a logistic regression model. We apply FedAvg (Algorithm 1), PurelocalTraining, and FedAvg followed by fine tuning, as well as FedProx (Algorithm 2) to this collection of datasets.

For FedAvg, we assume full participation (i.e., ${𝒞}_{t}=[m]$) and we set the number of communication rounds T=20 and global step size $\eta_{t}=0.8$. In its local training stage, we run SGD for 5 epochs with step size 0.2. For PureLocalTraining, we run SGD with step size 0.2 for 20 ⋅ 5=100 epochs. For the fine tuning strategy, we first run FedAvg (with the same hyperparameter as the previous case) and then for each client run SGD for 15 epochs with step size 0.2. For FedProx, we again assume full participation, and we set the number of communication rounds T=20, global step size $\eta_{t}^{\text{global}}=0.8$, local rounds $K_{t}=5$, and local step size $\eta_{t,k}^{\left(i\right)}=0.2$. In all the experiments, the batch size is set to 16.
